# Chaperone Hsp70 helps *Salmonella* survive infection-relevant stress by reducing protein synthesis

**DOI:** 10.1371/journal.pbio.3002560

**Published:** 2024-04-04

**Authors:** Carissa Chan, Eduardo A. Groisman

**Affiliations:** Department of Microbial Pathogenesis, Yale School of Medicine, New Haven, Connecticut, United States of America; Brigham and Women’s Hospital, UNITED STATES

## Abstract

In all domains of life, Hsp70 chaperones preserve protein homeostasis by promoting protein folding and degradation and preventing protein aggregation. We now report that the Hsp70 from the bacterial pathogen *Salmonella enterica* serovar Typhimurium—termed DnaK—independently reduces protein synthesis in vitro and in *S*. Typhimurium facing cytoplasmic Mg^2+^ starvation, a condition encountered during infection. This reduction reflects a 3-fold increase in ribosome association with DnaK and a 30-fold decrease in ribosome association with trigger factor, the chaperone normally associated with translating ribosomes. Surprisingly, this reduction does not involve J-domain cochaperones, unlike previously known functions of DnaK. Removing the 74 C-terminal amino acids of the 638-residue long DnaK impeded DnaK association with ribosomes and reduction of protein synthesis, rendering *S*. Typhimurium defective in protein homeostasis during cytoplasmic Mg^2+^ starvation. DnaK-dependent reduction in protein synthesis is critical for survival against Mg^2+^ starvation because inhibiting protein synthesis in a *dnaK*-independent manner overcame the 10,000-fold loss in viability resulting from DnaK truncation. Our results indicate that DnaK protects bacteria from infection-relevant stresses by coordinating protein synthesis with protein folding capacity.

## Abstract 2

Hsp70 chaperones preserve protein synthesis by promoting protein folding and preventing its aggregation. This study reports a new role of Hsp70 in Salmonella enterica survival during low Mg2+ conditions by binding to the ribosome and reducing protein synthesis.

## Introduction

Molecular chaperones play a vital role in all cell types. Chaperones increase the amounts of working proteins by promoting [1] and maintaining [2] their active conformations as well as helping them reach their subcellular locations [3], and by refolding [4] or eliminating [5] misfolded proteins, which can be highly toxic [6]. The ubiquitously distributed chaperones known as 70-kDa heat shock proteins (Hsp70s) function both with 40-kDa heat shock protein (Hsp40; J-domain) cochaperones, which deliver protein substrates to Hsp70s and stimulate their ATPase activity >1,000-fold [7], and with nucleotide exchange factors, which facilitate the exchange of adenosine diphosphate (ADP) for adenosine triphosphate (ATP), thereby allowing successive rounds of ATP hydrolysis [8]. Hsp70 defects are implicated in several diseases, reflecting Hsp70s’ roles in critical cellular functions [9–11]. The bacterial Hsp70 (i.e., DnaK) participates in multiple physiological processes, including cell division [12], virulence [13], and heat shock response [14], reflecting DnaK’s proposed function as a central hub in the bacterial chaperone network [15]. Here, we reveal that DnaK reduces protein synthesis and that this reduction is needed for bacterial survival against cytoplasmic Mg^2+^ starvation, a stress some pathogens face inside mammalian cells [16].

**Fig 1:**
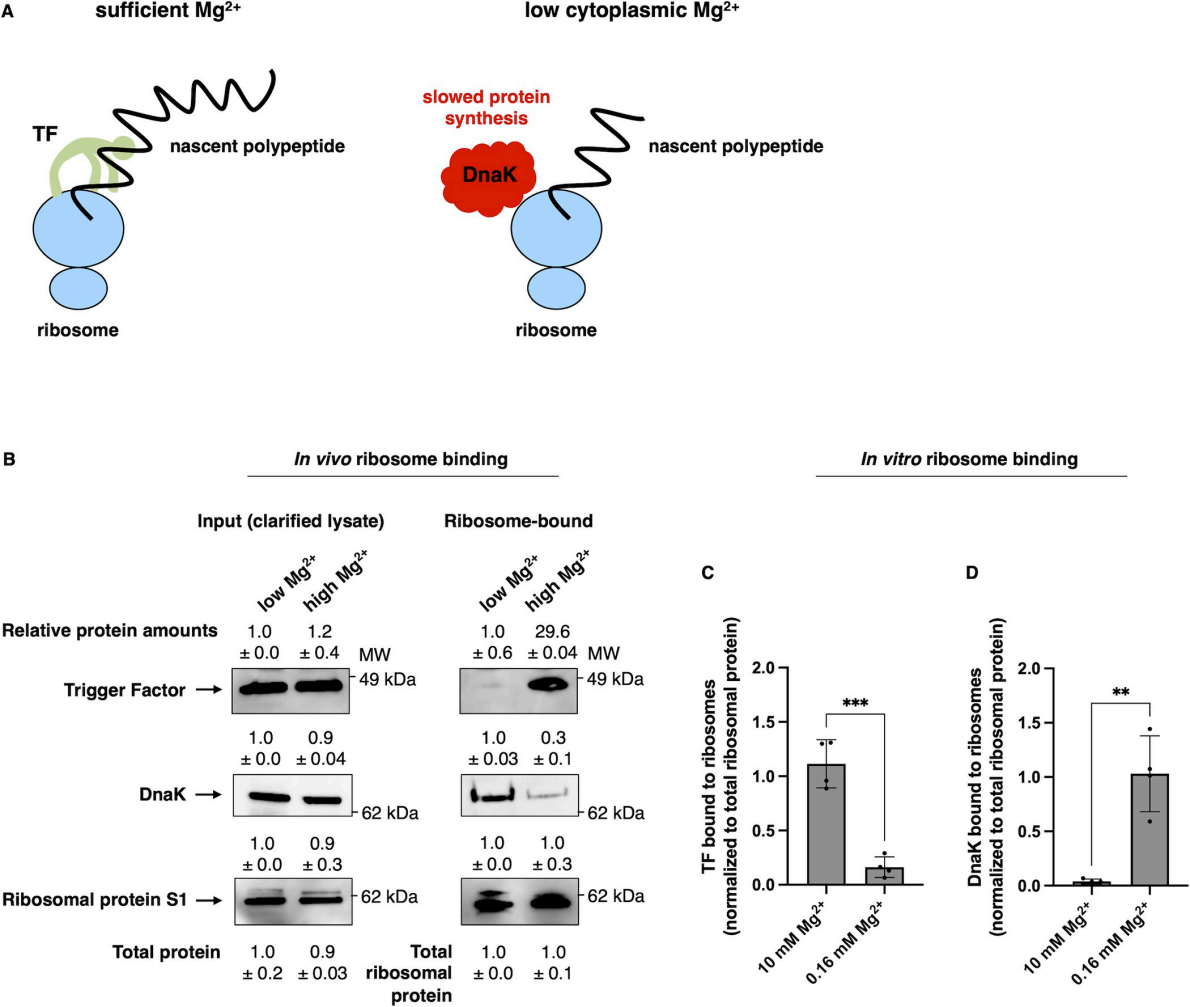
Decreased trigger factor association and increased DnaK association with ribosomes furthers protein homeostasis during Mg^2+^ starvation. **(A)** Schematic depicting how a shift in ribosome-associated chaperones in *S*. Typhimurium promotes protein homeostasis during stress. (Left) When cytoplasmic Mg^2+^ is abundant, protein synthesis proceeds at a normal rate and the ribosome-associated chaperone TF interacts with nascent polypeptides. (Right) When cytoplasmic Mg^2+^ is low, chaperone DnaK associates with ribosomes in place of TF and represses protein synthesis. **(B)** Western blot analysis of clarified cell lysates (left) and ribosome-bound fractions (right) of wild-type *S*. Typhimurium (14028s) following 5 h growth in low (10 μM) or 4.5 h of growth in high (10 mM) Mg^2+^ using antibodies recognizing DnaK, TF, or the ribosomal protein control S1. Total protein amounts in samples were quantified to verify equivalent sample loading. **(C, D)** Quantification of purified TF **(C)** or purified DnaK **(D)** bound to translating ribosomes in vitro in buffer containing 10 mM or 0.16 mM Mg^2+^. Shown in **(B)** are the representatives of 3 assays. Numerical values represent mean ± SD. MW = molecular weight. Data in **(C and D)** represent mean ± SD of 4 independent assays. Statistical analysis was performed using two-tailed Student’s *t* test comparing the indicated sample groups. The numerical values underlying this figure can be found in S1 Data.

When bacteria experience nutrient-rich conditions, the highly abundant chaperone trigger factor (TF) [17] binds to translating ribosomes, folding polypeptides cotranslationally as they emerge from the exit tunnel of the ribosome [18] (Fig 1A). A polypeptide not fully folded by TF is typically accessed by the downstream DnaK/DnaJ/GrpE chaperone system, consisting of DnaK, the Hsp40 DnaJ, and the nucleotide exchange factor GrpE [19]. Proteins requiring additional folding are subsequently transferred to the GroEL/GroES chaperone system [20].

The DnaK/DnaJ/GrpE system operates primarily posttranslationally [4] but can function cotranslationally, both in cooperation with TF [1] and in its place in a mutant lacking the TF-encoding *tig* gene [21]. The latter notion is supported by significant overlap between the proteins bound by DnaK and TF [22,23]. In addition, *tig* inactivation increases both DnaK abundance [23] and the amount of nascent polypeptides interacting with DnaK [22], and a *dnaK tig* double mutant cannot grow above 30°C even though the corresponding single mutants can [21]. While these findings suggest that DnaK and TF are partially redundant, an alternative conclusion is that these chaperones carry out similar (not identical) activities under different conditions.

The pathogen *Salmonella enterica* serovar Typhimurium (*S*. Typhimurium) increases DnaK amounts [24] and requires DnaK for survival [13] inside mammalian macrophages, an environment that provokes cytoplasmic Mg^2+^ starvation in this facultative intracellular pathogen [16]. Because low Mg^2+^ limits ATP-dependent proteolysis [25], ATP-dependent chaperoning [26], and ATP-dependent protein solubilization [27], and because DnaK aids adaptation to other stresses that disrupt proteostasis [13,28,29], we hypothesized that *S*. Typhimurium uses DnaK to maintain protein homeostasis when facing cytoplasmic Mg^2+^ starvation. We now report that DnaK associates with ribosomes and reduces protein synthesis while TF association with ribosomes dramatically decreases during cytoplasmic Mg^2+^ starvation. Reduced protein synthesis is critical for survival against cytoplasmic Mg^2+^ starvation because inhibiting protein synthesis independently of DnaK overcame the 10,000-fold loss in viability resulting from *dnaK* disruption. Unexpectedly, the novel DnaK properties are independent of J-domain cochaperones. Our results identify Mg^2+^ starvation as an environment that specifically requires DnaK to adjust protein synthesis to protein folding capacity.

## Results

### Trigger factor association with ribosomes decreases while DnaK association increases during cytoplasmic Mg^2+^ starvation

We used sedimentation over a sucrose cushion to examine the association of chaperones TF and DnaK with ribosomes in wild-type *S*. Typhimurium facing either cytoplasmic Mg^2+^ starvation (5 h in 10 μM Mg^2+^)—a condition that triggers expression of 2 Mg^2+^ importers and decreases ATP amounts [30,31]—or Mg^2+^ abundance (4.5 h in 10 mM Mg^2+^). The amount of TF bound to ribosomes was approximately 30 times lower in the former condition than in the latter condition (Fig 1B). By contrast, 3 times more DnaK was bound to ribosomes during cytoplasmic Mg^2+^ starvation than under Mg^2+^-abundant conditions (Fig 1B). (Similar results were obtained when ribosome association of TF and DnaK was investigated by polysome profiling over a sucrose gradient (S1A and S1B Fig), revealing that DnaK associates with both monosomes and polysomes.)

We determined that the molar ratio of ribosome-bound DnaK to ribosomes is 2.94 (±0.72) in bacteria experiencing cytoplasmic Mg^2+^ starvation but only 1.13 (±0.38) in bacteria experiencing Mg^2+^ abundance (mean ± SD). Control experiments showed no DnaK association with ribosomes that were fully dismantled due to treatment with the ion chelator ethylenediaminetetraacetic acid (S1C and S1D Fig).

To investigate how Mg^2+^ availability directly impacts chaperone binding to ribosomes, we incubated purified DnaK or TF with ribosomes from the PURExpress coupled transcription-translation system [32] (see later section) and diluted the reactions to 10 mM or 0.16 mM Mg^2+^. In agreement with findings obtained in vivo (Fig 1B), approximately 30 times more DnaK sedimented with ribosomes in low Mg^2+^ samples than in Mg^2+^-abundant samples (Fig 1C). By contrast, approximately 10 times less TF associated with ribosomes in low Mg^2+^ than in Mg^2+^-abundant samples (Fig 1D).

The Mg^2+^-regulated changes in the chaperone associated with ribosomes raised the question: What is the physiological consequence(s) of DnaK binding to ribosomes in lieu of TF?

### DnaK reduces protein synthesis in bacteria facing low cytoplasmic Mg^2+^

To determine whether DnaK binding to ribosomes in place of TF alters protein synthesis in vivo, we compared the behavior of isogenic wild-type and *dnaK* mutant strains. To avoid the general growth defects (i.e., not specific to cytoplasmic Mg^2+^ starvation conditions) that result from deletion of the full *dnaK* coding region [33], we used a mutant isolated in our laboratory that harbors a transposon Tn*10*dCm insertion between nucleotides 1691 and 1692 of the 1917 nucleotide-long *dnaK* coding region. Designated *dnaK14*, this *dnaK* allele specifies a truncated DnaK protein (S2A Fig) that shares 100% amino acid identity in the first 564 residues with the 638 residue-long wild-type DnaK but lacks the 74 C-terminal residues because of a stop codon originating from the transposon Tn*10*dCm in frame with the *dnaK* coding region (S2B Fig). The truncated DnaK retains the full nucleotide-binding domain (NBD) and most of the substrate-binding domain (SBD) but is missing the 42 C-terminal residues within the a-helical lid subdomain of the SBD and the C-terminal 32 residues of the protein, which are predicted to be disordered and whose function is not well understood [34] (S2C Fig). Because the *dnaK14* mutant grew like wild-type *S*. Typhimurium in LB broth and LB agar media, it was used for the in vivo experiments described below.

**Fig 2:**
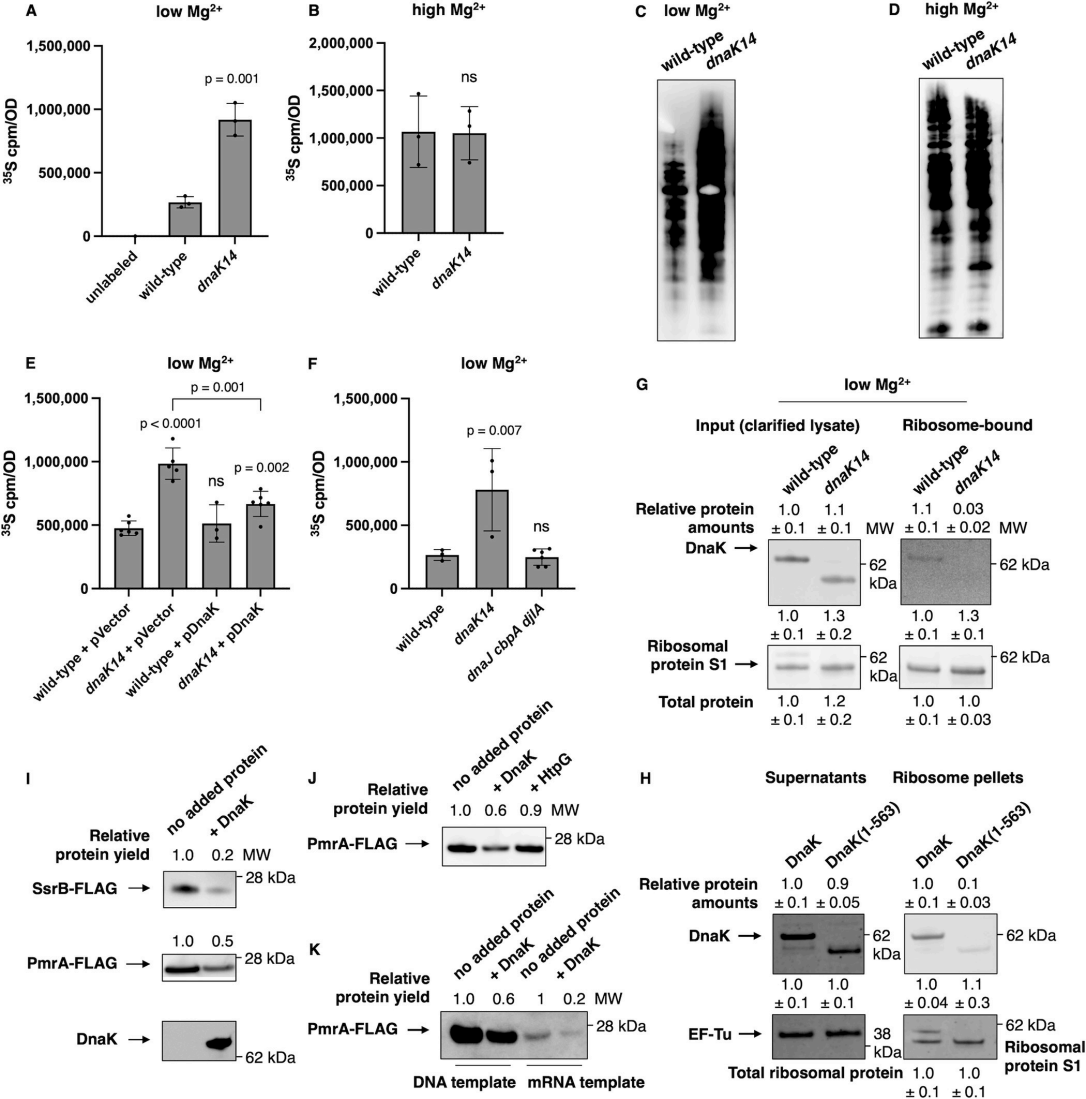
DnaK inhibits protein synthesis in vivo and in vitro. **(A, B)** ^35^S-methionine labeling of wild-type (14028s) and *dnaK14* (CC186) *S*. Typhimurium following 5 h of growth in low (10 μM) Mg^2+^ **(A)** or 4 h of growth in high (10 mM) Mg^2+^ **(B)**. **(C, D)** L-azidohomoalanine (AHA) labeling of wild-type (14028s) and *dnaK14* (CC186) *S*. Typhimurium following 5.5 h of growth in low (10 μM) Mg^2+^ **(C)** or 4.5 h of growth in high (10 mM) Mg^2+^**(D)**. **(E, F)** ^35^S-methionine labeling of wild-type (14028s) and *dnaK14* (CC186) *S*. Typhimurium harboring the plasmid vector (pUHE-21-2-*lacI*^*q*^) or the wild-type *dnaK*-expressing plasmid (pDnaK) following 5 h of growth in low (10 μM) Mg^2+^ **(E)** or of wild-type (14028s), *dnaK14* (CC186), and *dnaJ cbpA djlA* (CC656) *S*. Typhimurium following 5 h of growth in low (10 μM) Mg^2+^ **(F)**. **(G)** Western blot analysis of clarified cell lysates (left) and ribosome-bound fractions (right) of wild-type (14028s) and *dnaK14* (CC186) *S*. Typhimurium following 5 h of growth in low (10 μM) Mg^2+^ using antibodies recognizing DnaK or the ribosomal protein control S1. **(H)** Western blot analysis of purified DnaK protein or truncated DnaK (1–563) (5 μM) bound to translating ribosomes in vitro. Blot was developed with antibodies directed to the DnaK protein (ribosome-bound DnaK) or stained for total protein (supernatant fractions and ribosome-bound S1 protein; band above S1 represents the DnaK protein). **(I)** Western blot analysis of in vitro synthesized SsrB-FLAG and PmrA-FLAG proteins in the presence or absence of purified DnaK protein (5 μM). Blots were developed with antibodies directed to the FLAG tag or the DnaK protein. **(J)** Western blot analysis of in vitro synthesized PmrA-FLAG protein in the presence or absence of purified DnaK (5 μM) or purified HtpG (5 μM) proteins. **(K)** Western blot analysis of in vitro synthesized PmrA-FLAG protein in the presence or absence of purified DnaK (5 μM) using linear DNA or mRNA templates. Data in **(A–B** and **E–F)** represent mean ± SD of at least 3 independent biological replicates. Shown in **(C and D)** are the representatives of 3 independent biological replicates. Shown in **(G)** is the representative of 2 independent biological replicates. Total protein amounts in samples were quantified to verify equivalent sample loading. Numerical values represent mean ± SD. Shown in **(H)** is the representative of 4 assays. Numerical values represent mean ± SD. Shown in **(I–K)** are the representatives of 2 to 4 assays performed in buffer containing 9 mM Mg^2+^. Statistical analysis was performed using two-tailed Student’s *t* test comparing the indicated sample group to the wild-type sample group or comparing the bracketed sample groups (ns = not significant). MW = molecular weight. The numerical values underlying this figure can be found in S1 Data.

Pulse labeling of nascent proteins with ^35^S-methionine followed by quantification of the radioactivity in the protein precipitate revealed that the *dnaK14* mutant exhibited approximately 3-fold higher ^35^S-methionine incorporation than the wild-type strain (Fig 2A) in bacteria experiencing cytoplasmic Mg^2+^ starvation, indicating that the *dnaK14* mutant synthesized more protein than the wild-type strain. This novel phenotype is specific to cytoplasmic Mg^2+^ starvation conditions because wild-type and *dnaK14* strains exhibited similar ^35^S-methionine incorporation when Mg^2+^ was abundant (Fig 2B). Independent support for these findings was obtained by labeling nascent proteins with the click chemistry-compatible methionine analog L-azidohomoalanine: the *dnaK14* mutant incorporated more methionine analog than wild-type *S*. Typhimurium in low Mg^2+^ (Fig 2C), but not when Mg^2+^ was abundant (Fig 2D).

The increased protein synthesis exhibited by the *dnaK14* mutant is due to the disruption of the *dnaK* gene rather than polarity on the downstream *dnaJ* gene [35,36] because plasmid pDnaK, which expresses a wild-type copy of the *dnaK* gene from a heterologous promoter, largely complemented the mutant, whereas the plasmid vector control did not (Fig 2E). Moreover, a *S*. Typhimurium triple mutant lacking the *cbpA*, *djlA*, and *dnaJ* genes, which encode the 3 J-domain cochaperones of DnaK characterized in the closely related species *Escherichia coli* [37], exhibited similar ^35^S-methionine incorporation to the wild-type strain (Fig 2F), unlike the increased ^35^S-methionine incorporation of the *dnaK14* mutant (Fig 2A, 2E and 2F).

DnaK association with ribosomes (Fig 1B and 1C) appears to be responsible for the decreased protein synthesis exhibited by wild-type *S*. Typhimurium experiencing cytoplasmic Mg^2+^ starvation (Fig 2A–2D) because the truncated DnaK produced by the *dnaK14* strain did not associate with ribosomes (Fig 2G) in vivo. Furthermore, purified truncated DnaK (consisting of the first 563 amino acids specified by native codons) bound translating ribosomes from the PURExpress coupled transcription-translation system [32] (see next section) approximately 10 times less than purified full-length DnaK (Fig 2H).

Because the remaining C-terminal tail portion of some truncated forms of DnaK can interact with its own SBD [38], we explored whether the lack of association between DnaK(1–563) and ribosomes was caused by the truncated DnaK occupying its own SBD binding pocket. Control experiments established that the basal in vitro ATPase activity (without J-domain cochaperones or nucleotide exchange factor) of purified truncated DnaK is similar to that of full-length DnaK (S2D Fig). These results indicate that the C-terminal portion remaining in the truncated DnaK does not mimic a peptide substrate and that truncated DnaK does not exist in a tail-bound state [38], arguing against the possibility that truncated DnaK fails to bind ribosomes due to a self-occupied substrate binding site. (Note that the ATPase activity of the truncated DnaK was stimulated less by J-domain cochaperones and nucleotide exchange factor than the ATPase activity of the full-length wild-type DnaK [S2D Fig].)

Cumulatively, the results in this section show that *S*. Typhimurium responds to cytoplasmic Mg^2+^ starvation by increasing DnaK association with ribosomes, which reduces protein synthesis.

### Purified DnaK reduces protein synthesis in vitro

To establish whether DnaK is directly responsible for the reduction in protein synthesis observed in vivo (Fig 2A), we examined the effect of the purified DnaK protein on protein synthesis in vitro. We used the PURExpress in vitro protein synthesis system [32], which consists of His-tagged proteins necessary for coupled transcription/translation, untagged ribosomes, no added chaperones, and 9 mM Mg^2+^ [32], which is necessary for transcription and translation to take place, and verified that DnaK preparations lacked contaminating proteins (S3A Fig), DNase activity (S3B and S3C Fig), or RNase activity (S3D and S3E Fig). Reactions were programmed with DNA templates specifying C-terminally FLAG-tagged reporter proteins, selected because they are expressed by *S*. Typhimurium during low Mg^2+^ [39,40]. Purified DnaK decreased the amounts of synthesized proteins up to 5-fold relative to controls consisting of the buffer used to resuspend DnaK (Fig 2I) or the protein chaperone HtpG (Fig 2J), known as the bacterial Hsp90 [41]. The yield of synthesized proteins decreased correspondingly with increasing DnaK concentrations (S3F Fig).

DnaK also decreased the amounts of synthesized proteins when mRNA was used instead of DNA (Fig 2K), indicating that DnaK inhibits translation and arguing against the possibility that the phenotypes obtained with DNA templates resulted from DnaK inhibiting gene transcription of the coupled transcription/translation PURExpress system [32]. That the DnaK preparations appear to be free of DnaJ (S3A Fig) and that the truncated DnaK retains DnaJ’s binding domain [42] (S2A–S2C Fig) argue that DnaK’s inhibition of protein synthesis does not require cochaperone DnaJ.

### Trigger factor interferes with DnaK’s reduction of protein synthesis

**Fig 3:**
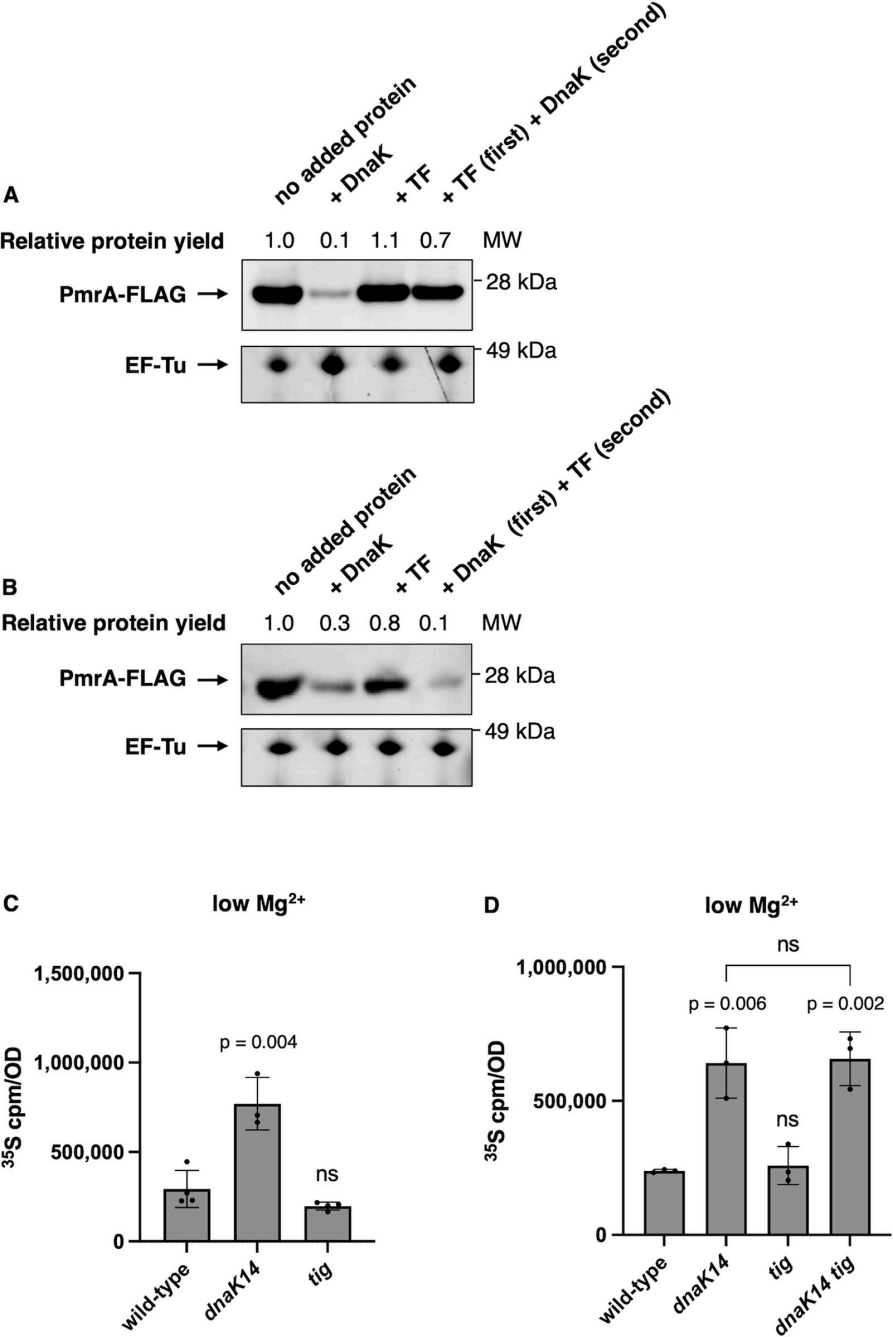
Trigger factor hinders DnaK-mediated reduction of protein synthesis. **(A)** Western blot analysis of in vitro synthesized PmrA-FLAG protein in the absence or presence of purified DnaK, purified TF, or purified TF added before purified DnaK addition (5 μM each protein). Blot was developed with antibodies directed to the FLAG tag. Total protein staining was performed to visualize translation factor EF-Tu as a loading control. **(B)** Western blot analysis of in vitro synthesized PmrA-FLAG protein in the absence or presence of purified DnaK, purified TF, or purified DnaK added before purified TF (5 μM each protein). Blot was developed with antibodies directed to the FLAG tag. Total protein staining was performed to visualize translation factor EF-Tu as a loading control. **(C)** ^35^S-methionine labeling of wild-type (14028s) *dnaK14* (CC186) and *tig* (CC361) *S*. Typhimurium following 5 h of growth in low (10 μM) Mg^2+^. **(D)** ^35^S-methionine labeling of wild-type (14028s) *dnaK14* (CC186), *tig* (CC361), and *dnaK tig* (CC362) *S*. Typhimurium following 6 h of growth in low (10 μM) Mg^2+^ at 30°C. Shown in **(A and B)** are the representatives of 2 to 3 assays performed in buffer containing 9 mM Mg^2+^. MW = molecular weight. Data in **(C and D)** represent mean ± SD of at least 3 independent biological replicates. Statistical analysis was performed using two-tailed Student’s *t* test comparing the indicated sample group to the wild-type sample group or comparing the bracketed sample groups (ns = not significant). The numerical values underlying this figure can be found in S1 Data.

When the protein synthesis machinery was incubated with purified TF prior to addition of DnaK, the yield of the in vitro protein synthesis reaction was higher than when DnaK was added alone (Fig 3A). By contrast, TF had no effect when DnaK was added to the reaction prior to TF (Fig 3B). Though these results suggest the possibility of DnaK and TF sharing binding sites in the translation machinery, an alternative interpretation is that binding of one of these chaperones to the translation machinery results in conformational changes that hinder the ability of the other chaperone to reduce protein synthesis. Control experiments demonstrated that TF neither increases nor decreases protein synthesis when added alone (i.e., in the absence of DnaK) to the PURExpress system (Fig 3A and 3B).

A mutant lacking the *tig* coding region displayed wild-type protein synthesis during cytoplasmic Mg^2+^ starvation (Fig 3C), in agreement with the in vitro results discussed above (Fig 3A and 3B). By contrast, when the *tig* deletion was combined with the *dnaK14* mutation, the resulting double mutant had the same elevated protein synthesis as the *dnaK14* single mutant (Fig 3D). (The latter experiment was performed at 30°C because a *tig dnaK* mutant is not viable at higher temperatures [21].) Cumulatively, these results likely reflect that little TF associates with ribosomes in bacteria experiencing cytoplasmic Mg^2+^ starvation (Fig 1B and 1D) to interfere with the reduction of protein synthesis mediated by DnaK.

### DnaK’s ATPase activity is stimulated by ribosomes and required for reduction of protein synthesis

**Fig 4:**
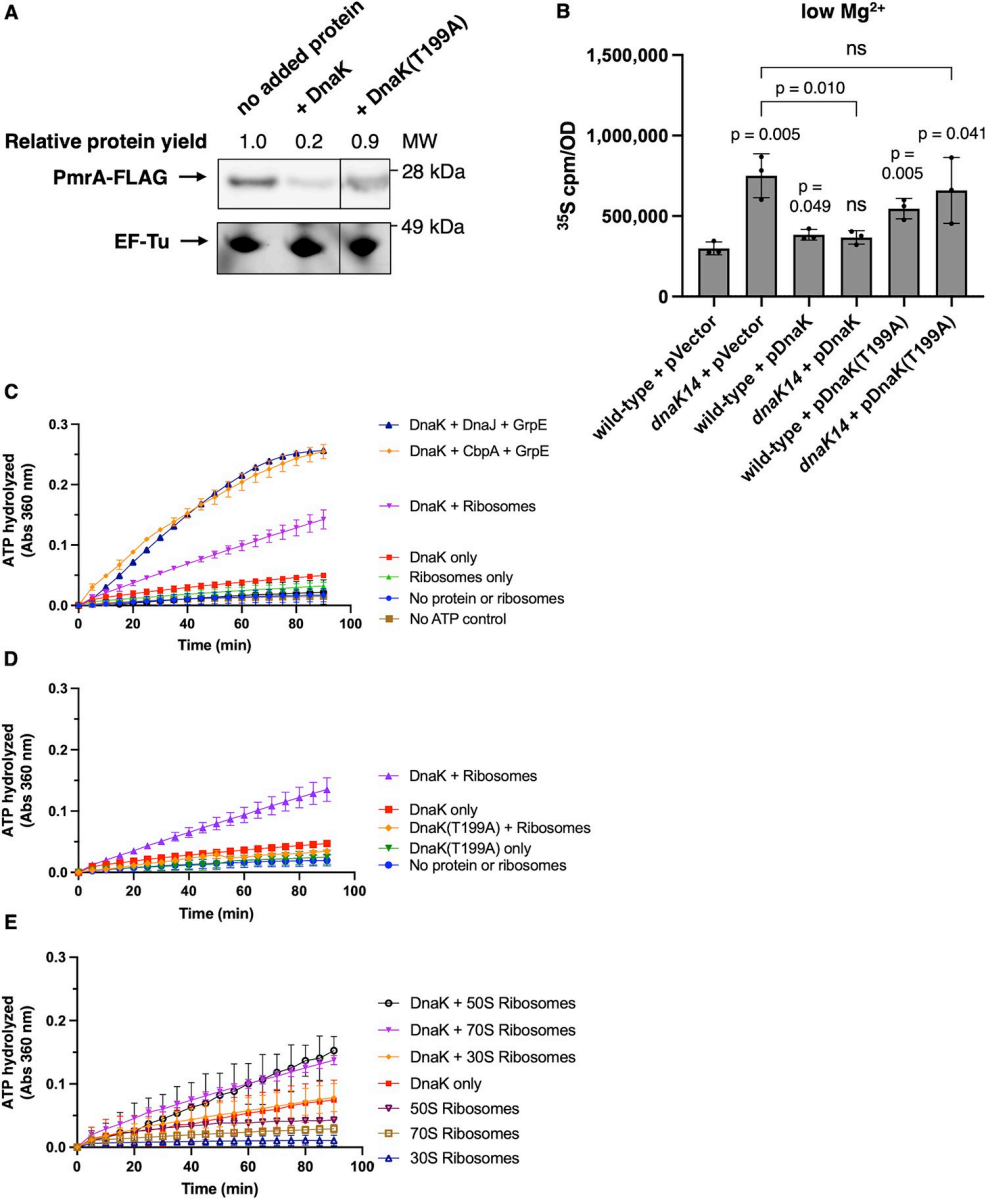
DnaK’s ATPase activity is stimulated by ribosomes and required to reduce protein synthesis. **(A)** Western blot analysis of in vitro synthesized PmrA-FLAG protein in the presence or absence of purified DnaK or DnaK(T199A) (5 μM). Blot was developed with antibodies directed to the FLAG tag. Total protein staining was performed to visualize translation factor EF-Tu as a loading control. **(B)** ^35^S-methionine labeling of wild-type (14028s) and *dnaK14* (CC186) *S*. Typhimurium harboring the plasmid vector (pUHE-21-2-*lacI*^*q*^), wild-type *dnaK*-expressing plasmid (pDnaK), or mutant *dnaK*(T199A)-expressing plasmid (pDnaKT199A) following 5 h of growth in low (10 μM) Mg^2+^. **(C–E)** ATP hydrolysis in the presence or absence of purified DnaK (2 μM) alone or in combination with cochaperones (DnaJ [0.4 μM] or CbpA [0.4 μM] and GrpE [0.2 μM]) or 70S ribosomes (0.5 μM) **(C)**, in the presence or absence of purified DnaK(T199A) (2 μM) alone or in combination with 70S ribosomes (0.5 μM) **(D)** or in the presence or absence of purified DnaK (2 μM) alone or in combination with 30S ribosomal subunit (0.5 μM), 50S ribosomal subunit (0.5 μM), or 70S ribosomes (0.5 μM) **(E)**. Shown in **(A)** is the representative of 3 assays. MW = molecular weight. Data in **(B)** represents mean ± SD of 3 independent biological replicates. Data in **(C–E)** represent mean ± SD of 2 to 4 independent assays performed in buffer containing 20 mM Mg^2+^. Absorbance at 360 nm reflects the production of 2-amino-6-mercapto-7-methylpurine stimulated by the presence of inorganic Pi, the product of the ATPase reaction. Statistical analysis in **(B)** was performed using two-tailed Student’s *t* test comparing the indicated sample group to the wild-type sample group or comparing the bracketed sample groups (ns = not significant). The numerical values underlying this figure can be found in S1 Data.

DnaK requires ATP hydrolysis for substrate binding and release (reviewed in [43,44]). J-domain cochaperones stimulate DnaK’s ATPase activity [7]. We have now determined that a purified variant of full-length DnaK unable to hydrolyze ATP [45,46]—DnaK(T199A)—failed to reduce protein synthesis in vitro, unlike wild-type DnaK (Fig 4A). In vivo, a DnaK(T199A)-expressing plasmid failed to fully correct the abnormally high protein synthesis of the *dnaK14* mutant, behaving like the vector control and unlike the isogenic plasmid pDnaK (Fig 4B). Thus, DnaK’s ability to reduce protein synthesis requires: its ability both to associate with ribosomes, which is dependent on its C-terminal 74 residues (Fig 2G and 2H), and to hydrolyze ATP (Fig 4A), which is conferred by the N-terminal domain of the protein [47].

We established that purified bacterial ribosomes (New England Biolabs) stimulate DnaK’s ATPase activity in the absence of J-domain cochaperones (Fig 4C), albeit approximately 45% less than J-domain cochaperones and GrpE (Fig 4C). ATP hydrolysis was not detected when ribosomes were incubated with the catalytic-defective mutant DnaK(T199A) [45] (Fig 4D), arguing against the possibility of wild-type DnaK stimulating a hypothetical ATPase contaminating the ribosome preparation. The large (50S) subunit of the ribosome, which harbors TF’s binding site [18], stimulated ATP hydrolysis as much as intact 70S ribosomes (Fig 4E), whereas the small (30S) subunit had no effect on DnaK’s ATPase activity (Fig 4E). (These assays were conducted in buffer containing 20 mM Mg^2+^.)

Control experiments indicate that the ribosome-stimulated ATP hydrolysis by DnaK is not due to sample contamination with a J-domain cochaperone because DnaK alone failed to refold heat-denatured luciferase in the presence or absence of ribosomes (S4 Fig), behaving like the no protein control (S4 Fig), and unlike the positive control consisting of DnaK, J-domain cochaperone CbpA, and GrpE, which efficiently refolded luciferase (S4 Fig).

Taken together, the results in this section demonstrate that ribosomes stimulate ATP hydrolysis by DnaK; that this stimulation is independent of J-domain chaperones; and that DnaK’s ATPase activity is required for reduction of protein synthesis.

### DnaK prevents protein aggregation during cytoplasmic Mg^2+^ starvation

Cells must adjust their rate of protein synthesis to their protein folding capacity or face the toxic effects of accumulating misfolded protein aggregates [48–50]. We reasoned that DnaK coordinates these 2 activities when bacteria face cytoplasmic Mg^2+^ starvation because this stressor limits protein homeostatic capacity by decreasing the concentration of ATP [51], thereby reducing ATP-dependent proteolysis [25], ATP-dependent chaperoning [26], and ATP-dependent protein solubilization [27]. Moreover, DnaK reduces protein synthesis in vitro (Fig 2I) and during cytoplasmic Mg^2+^ starvation in vivo (Fig 2A and 2B). These findings suggested that DnaK helps maintain protein homeostasis in bacteria facing cytoplasmic Mg^2+^ starvation.

**Fig 5:**
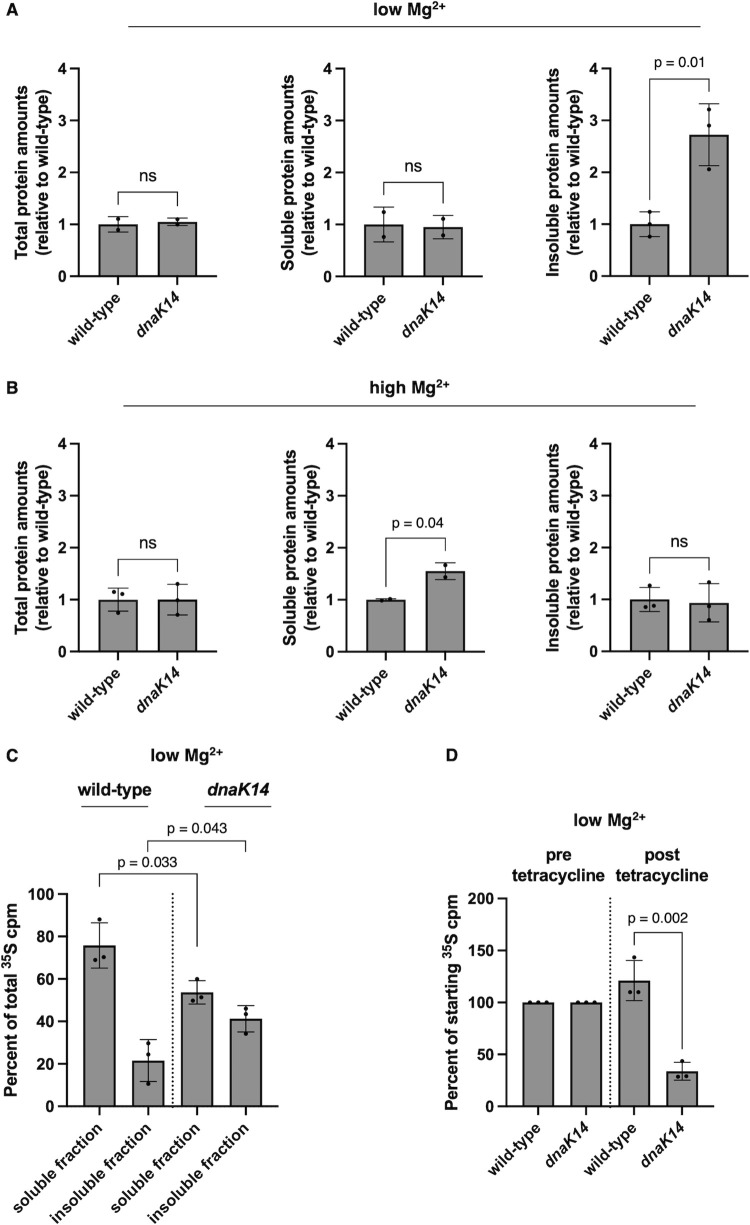
*S*. Typhimurium coordinates protein synthesis with protein folding capacity. **(A, B)** Quantification of total, soluble, and insoluble protein amounts in wild-type (14028s) and *dnaK14* (CC186) *S*. Typhimurium following 6 h of growth in low (10 μM) Mg^2+^ **(A)** or 4.5 h of growth in high (10 mM) Mg^2+^ **(B)**. **(C)** Percent of ^35^S-methionine-labeled proteins in soluble and insoluble fractions isolated from wild-type (14028s) and *dnaK14* (CC186) *S*. Typhimurium following 5 h of growth in low (10 μM) Mg^2+^. **(D)** Percent of ^35^S-methionine-labeled proteins remaining in wild-type (14028s) and *dnaK14* (CC186) *S*. Typhimurium following 18 h of treatment with 62.5 μg/ml tetracycline to halt translation. Values were calculated as the amount of ^35^S-methionine signal remaining divided by the ^35^S-methionine signal of a sample collected immediately prior to tetracycline addition. Data in **(A)** and (**B)** represent mean ± SD of at least 2 independent biological replicates. Data in **(C)** and (**D)** represent mean ± SD of 3 independent biological replicates. Statistical analysis was performed using two-tailed Student’s *t* test comparing the bracketed sample groups (ns = not significant). The numerical values underlying this figure can be found in S1 Data.

As hypothesized, the *dnaK14* mutant had more protein aggregates than the wild-type strain following 6 h in low Mg^2+^ medium (Fig 5A), when bacteria face cytoplasmic Mg^2+^ starvation [51,52]. By contrast, protein aggregate amounts in Mg^2+^-abundant conditions were not elevated in the *dnaK14* mutant (Fig 5B). Moreover, the fraction of newly synthesized proteins (determined by ^35^S-methionine-labeling) present in the insoluble fraction of bacteria facing cytoplasmic Mg^2+^ starvation was twice as high in the *dnaK14* mutant as in the wild-type strain (Fig 5C).

We then monitored the fate of ^35^S-methionine-labeled proteins in bacteria grown in low (10 μM) Mg^2+^ medium before and after treatment with the protein synthesis inhibitor tetracycline (62.5 μg/ml). The amounts of radiolabeled proteins remained stable in the wild-type strain 18 h after tetracycline treatment but decreased by approximately 60% in the *dnaK* mutant relative to starting values (Fig 5D), indicating decreased global protein stability.

These results indicate that DnaK’s reduction in protein synthesis during cytoplasmic Mg^2+^ starvation aids protein homeostasis by preventing protein synthesis from exceeding protein folding capacity.

### DnaK is necessary for survival against cytoplasmic Mg^2+^ starvation and hyperosmotic stress

**Fig 6:**
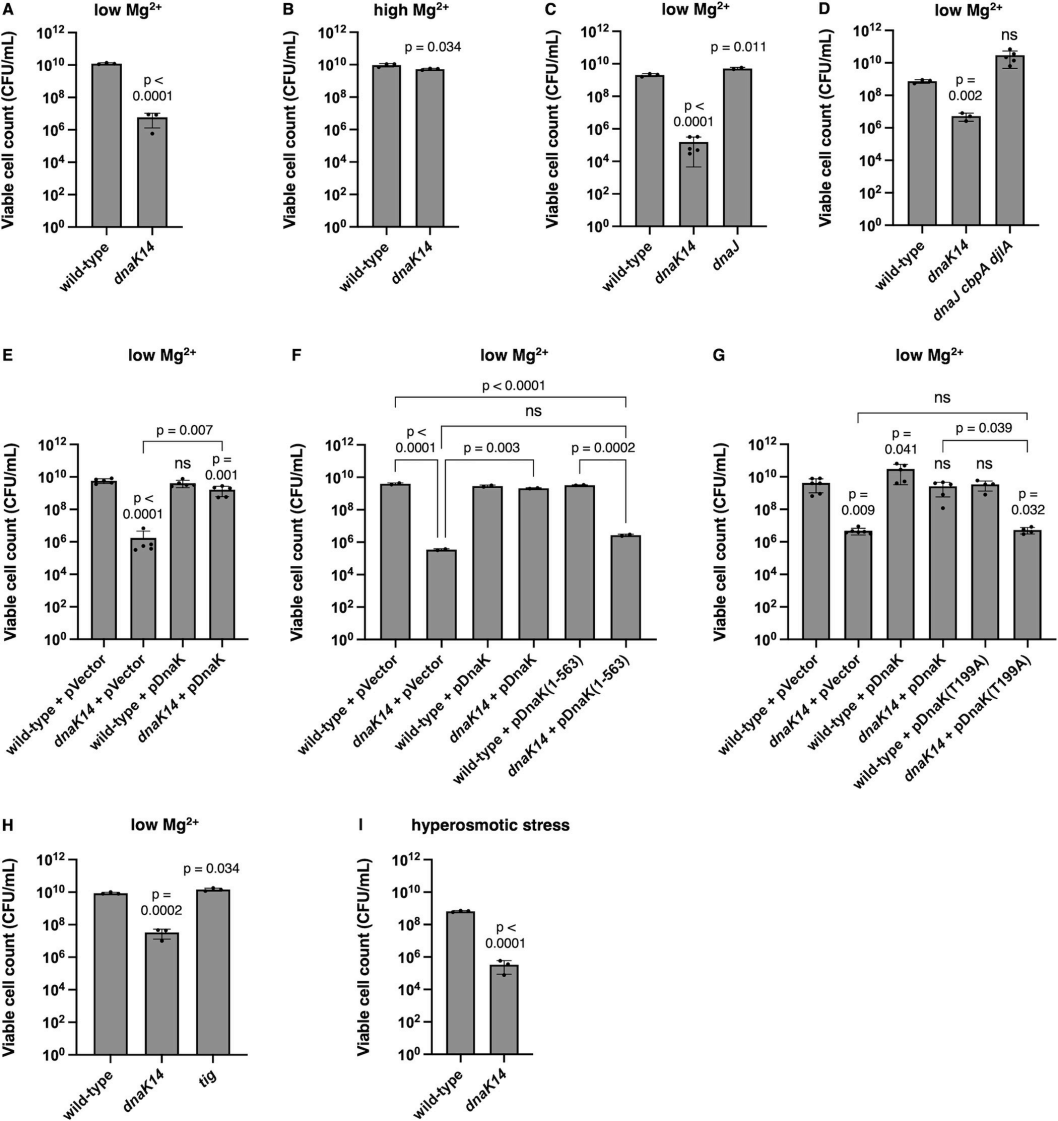
The *dnaK* gene is required for bacterial survival against Mg^2+^ starvation. **(A, B)** Survival of wild-type (14028s) and *dnaK14* (CC186) *S*. Typhimurium following 24 h in low (10 μM) Mg^2+^ **(A)** or 24 h in high (10 mM) Mg^2+^ **(B)**. **(C)** Survival of wild-type (14028s), *dnaK14* (CC186), and *dnaJ* (EG16309) *S*. Typhimurium following 24 h in low (10 μM) Mg^2+^. **(D)** Survival of wild-type (14028s), *dnaK14* (CC186), and *dnaJ cbpA djlA* (CC656) *S*. Typhimurium following 24 h in low (10 μM) Mg^2+^. **(E–H)** Survival of wild-type (14028s) and *dnaK14* (CC186) *S*. Typhimurium harboring the plasmid vector (pUHE-21-2-*lacI*^*q*^) or the *dnaK-*expressing plasmid (pDnaK) **(E)**, of wild-type (14028s) and *dnaK14* (CC186) *S*. Typhimurium harboring the plasmid vector (pUHE-21-2-*lacI*^*q*^), wild-type *dnaK-*expressing plasmid (pDnaK), or *dnaK*(nucleotides 1–1691)-expressing plasmid following 24 h in low (10 μM) Mg^2+^ **(F)**, of wild-type (14028s) and *dnaK14* (CC186) *S*. Typhimurium harboring the plasmid vector (pUHE-21-2-*lacI*^*q*^), wild-type *dnaK-*expressing plasmid (pDnaK), or *dnaK*(T199A)-expressing plasmid (pDnaKT199A) **(G)**, or of wild-type (14028s), *dnaK14* (CC186), and *tig* (CC361) *S*. Typhimurium **(H)** following 24 h in low (10 μM) Mg^2+^. **(I)** Survival of wild-type (14028s) and *dnaK14* (CC186) *S*. Typhimurium following 24 h in hyperosmotic (1 M NaCl) conditions. Data in **(A–I)** represent mean ± SD of at least 3 independent biological replicates. Statistical analysis was performed using two-tailed Student’s *t* test comparing the indicated sample group to the wild-type sample group or comparing the bracketed sample groups (ns = not significant). The numerical values underlying this figure can be found in S1 Data.

DnaK is required for survival against cytoplasmic Mg^2+^ starvation because the *dnaK14* mutant was approximately 10,000-fold less viable than wild-type *S*. Typhimurium following a 24 h incubation in low (10 μM) Mg^2+^ medium (Fig 6A). This survival defect is specific to low Mg^2+^ because the mutant exhibited wild-type survival in medium containing high (10 mM) Mg^2+^ (Fig 6B).

The survival defect of the *dnaK14* mutant is not due to polar effects on *dnaJ* because: first, a *dnaJ* mutant exhibited wild-type survival in low Mg^2+^ conditions (Fig 6C), as did the *dnaJ cbpA djlA* triple mutant (Fig 6D); and second, plasmid pDnaK rescued the *dnaK14* mutant (Fig 6E). By contrast, neither the plasmid vector control nor plasmid pDnaK(1–563) carrying the native codons corresponding to the first 563 residues of DnaK, thus specifying the truncated DnaK, rescued the *dnaK14* mutant for survival in low Mg^2+^ (Fig 6E and 6F). (Wild-type *S*. Typhimurium exhibited similar survival when harboring pDnaK, the vector control, or pDnaK(1–563), indicating that the truncated DnaK protein is not dominant negative on wild-type DnaK [Fig 6E]. In addition, wild-type and *dnaK14* mutant *S*. Typhimurium harboring the plasmid vector or plasmid pDnaK exhibited similar survival during growth in high Mg^2+^. [S5A Fig])

DnaK’s ability to hydrolyze ATP is required for bacterial survival against cytoplasmic Mg^2+^ starvation because plasmid pDnaK(T199A) failed to restore wild-type survival to the *dnaK14* mutant, behaving like the vector control and unlike pDnaK (Fig 6G). While DnaK is required for bacterial survival against cytoplasmic Mg^2+^ starvation (Fig 6A), TF is dispensable because the *tig* mutant behaved like the wild-type strain (Fig 6H). In agreement with the results of others [21], TF was also not required for growth in nutrient abundant conditions (S5B Fig).

Hyperosmotic stress triggers transient cytoplasmic Mg^2+^ starvation even when Mg^2+^ in the environment is plentiful [53]. This is because bacteria respond to hyperosmotic stress by importing large amounts of the osmoprotective ion K^+^ [54], which is countered by Mg^2+^ efflux from the cytoplasm. We therefore hypothesized that a functional *dnaK* gene would be required to survive hyperosmotic stress. As expected, viability of the *dnaK14* mutant was approximately 100-fold lower than that of the wild-type strain following 1 day incubation in medium containing 1 M NaCl and 1 mM Mg^2+^ (which corresponds to the physiological Mg^2+^ concentration in the bacterial cytoplasm) [55] (Fig 6I).

Given the increased protein synthesis of the *dnaK14* mutant (Fig 2A–2D) and wild-type protein synthesis of the *tig* mutant (Fig 3C and 3D), the results in this section suggest that bacterial survival against cytoplasmic Mg^2+^ starvation requires DnaK’s ability to reduce protein synthesis.

### DnaK confers survival against cytoplasmic Mg^2+^ starvation by decreasing protein synthesis

**Fig 7:**
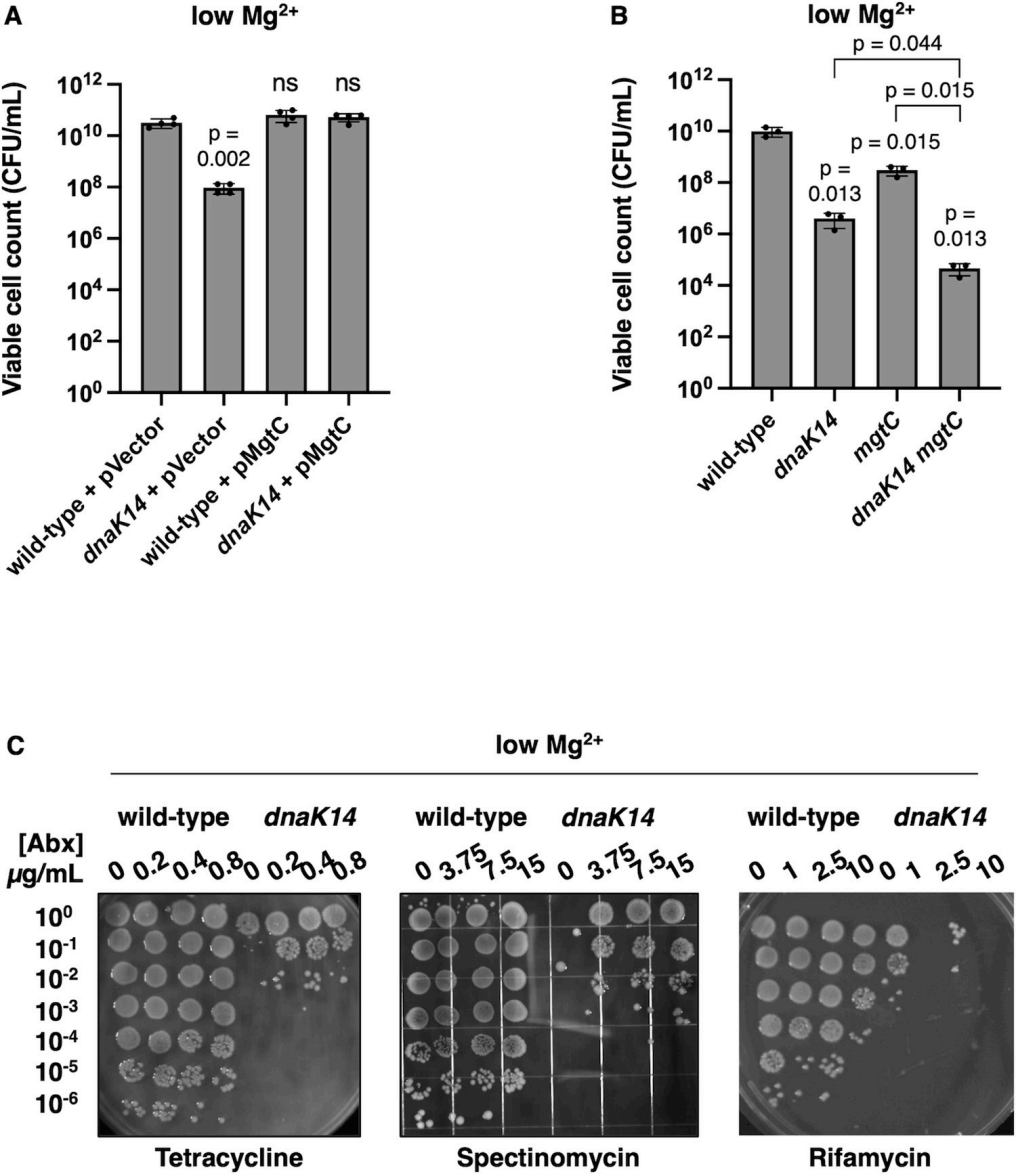
A reduction in protein synthesis furthers bacterial survival against Mg^2+^ starvation. **(A)** Survival of wild-type (14028s) and *dnaK14* (CC186) *S*. Typhimurium harboring the plasmid vector (pUHE-21-2-*lacI*^*q*^) or the *mgtC-*expressing plasmid (pMgtC) following 24 h of growth in low (10 μM) Mg^2+^. **(B)** Survival of wild-type (14028s), *dnaK14* (CC186), *mgtC* (EL1), and *dnaK mgtC* (CC241) *S*. Typhimurium following 24 h of growth in low (10 μM) Mg^2+^. **(C)** Survival of wild-type (14028s) and *dnaK14* (CC186) *S*. Typhimurium in the presence or absence of sublethal amounts of the protein synthesis inhibitors tetracycline or spectinomycin or the RNA polymerase inhibitor rifamycin following 24 h of growth in low (10 μM) Mg^2+^. Data in **(A and B)** represent mean ± SD of at least 3 independent biological replicates. Statistical analysis was performed using two-tailed Student’s *t* test comparing the indicated sample group to the wild-type sample group or comparing the bracketed sample groups (ns = not significant). Shown in **(C)** are the representatives of 3 assays. The numerical values underlying this figure can be found in S1 Data.

If DnaK confers survival against cytoplasmic Mg^2+^ starvation by decreasing protein synthesis, a *dnaK*-independent decrease in protein synthesis should rescue the *dnaK14* mutant. The results of 3 independent experimental approaches support this notion. First, we used plasmid pMgtC, which harbors a wild-type copy of the *mgtC* gene transcribed from a heterologous promoter. *mgtC* expression lowers ATP amounts and promotes accumulation of the second messenger (p)ppGpp (guanosine tetraphosphate), which decreases rRNA synthesis, reduces the number of ribosomes, and results in lower overall cellular protein synthesis [51]. When introduced into the *dnaK14* mutant, plasmid pMgtC, but not the plasmid vector, decreased protein synthesis (S6 Fig). Remarkably, pMgtC restored wild-type viability to the *dnaK14* mutant facing cytoplasmic Mg^2+^ starvation (Fig 7A), whereas the plasmid vector control did not (Fig 7A).

Second, inactivation of the *mgtC* gene in the *dnaK14* mutant background further decreased survival against cytoplasmic Mg^2+^ starvation (Fig 7B). This result likely reflects that MgtC and DnaK reduce protein synthesis by 2 distinct mechanisms: decreasing ribosome amounts [51] and activity (Figs 2I–2K, 3A, 3B, and 4A), respectively. And third, the bacteriostatic protein synthesis inhibitors tetracycline and spectinomycin increased survival of the *dnaK14* mutant when added at subinhibitory concentrations (Fig 7C). By contrast, a subinhibitory concentration of the RNA polymerase inhibitor rifamycin failed to rescue the mutant (Fig 7C), suggesting that survival is specifically restored by protein synthesis inhibitors.

## Discussion

We have now revealed novel biochemical activities and physiological functions for DnaK, the bacterial homolog of the highly conserved Hsp70 chaperone [56]. We established that DnaK associates with ribosomes and reduces protein synthesis (Figs 1 and 2), that this reduction is necessary for bacterial survival against cytoplasmic Mg^2+^ starvation (Fig 7), and that these uncovered DnaK properties are independent of J-domain cochaperones (Figs 2 and 6). By contrast, chaperone TF, which is bound to ribosomes under nutrient-abundant conditions, neither reduces protein synthesis (Fig 3) nor is required for survival (Fig 6G) in bacteria facing cytoplasmic Mg^2+^ starvation. Our findings indicate that TF and DnaK play distinct cellular roles even though both associate with ribosomes, albeit under different conditions.

During nutrient abundance, TF binds near the exit tunnel of actively translating ribosomes [17,57] and cooperates with the downstream DnaK/DnaJ/GrpE system to fold nascent polypeptides [1,21]. TF folds proteins cotranslationally in an ATP-independent fashion [17] and does not reduce protein synthesis (Fig 3). However, when bacteria experience cytoplasmic Mg^2+^ starvation, ribosome association with TF decreases approximately 30-fold while association with DnaK increases 3-fold (Fig 1B), raising the possibility that DnaK instead of TF aids folding of nascent polypeptides cotranslationally (Fig 1A–1D). This scenario is supported by the shared ability of TF and DnaK to interact with most cellular proteins [20] and by the consequences of *tig* and *dnaK* inactivation: single mutants survive 37°C, but the *tig dnaK* double mutant does not [21]. Alternatively or additionally, DnaK may promote protein folding by slowing down translation, manifested in our assays by decreased protein synthesis over a given time window (Fig 2), which aids chaperone-independent folding efficiency [58]. DnaK may also favor protein folding by increasing the ribosome’s ability to fold nascent polypeptides that remain tethered to the ribosome surface throughout synthesis [59].

It may seem paradoxical that DnaK requires ATP hydrolysis to reduce protein synthesis during cytoplasmic Mg^2+^ starvation (Fig 4) when cellular ATP concentration decreases significantly [31,51]. However, the relatively small ATP amount spent to reduce protein synthesis is lower than that required to degrade proteins by ATP-dependent proteases [60]. Moreover, hydrolyzing ATP to reduce protein synthesis most likely saves energy overall because protein synthesis is the most energy-expensive cellular process [61]. Furthermore, synthesizing proteins in the absence of adequate folding capacity necessarily leads to degradation of misfolded proteins and cellular toxicity [48,50]. In support of this notion, the *dnaK14* mutant unable to reduce protein synthesis (Fig 2A–2F) due to the lack of the 74 C-terminal amino acids of DnaK (S2 Fig) had more protein aggregates (Fig 5A and 5C) and less global protein stability (Fig 5D) than the wild-type strain when facing cytoplasmic Mg^2+^ starvation.

Hsp70s consist of a 45 kDa N-terminal NBD and a 30 kDa C-terminal SBD connected by a hydrophobic linker [34]. Curiously, the C-terminal 10% of the protein (residues 604–638 in DnaK) is more divergent than the rest (17% versus 53% conservation for the whole protein among a collection of 730 Hsp70s) and is predicted to be structurally disordered [62]. DnaK’s C-terminal 34 amino acids are required for *E*. *coli* survival at 43°C but dispensable for phage λ propagation at 30°C, peptide substrate binding, and co-chaperone interaction [62]. Because the truncated DnaK variant we investigated is missing the 74 C-terminal residues, it also lacks the 42 C-terminal residues within the α-helical lid subdomain of the SBD (S1B Fig), which may impact not only substrate binding to the SBD [62] but also association with ribosomes (Fig 2G and 2H), reduction of protein synthesis (Fig 2A and 2C), and survival against low Mg^2+^ (Fig 6).

DnaK is uniquely positioned to coordinate protein synthesis with folding capacity when cytoplasmic Mg^2+^ is limited because, unlike other protein chaperones, DnaK mediates cotranslational [1] as well as posttranslational protein folding [21] and prevents protein aggregation [15,63]. This strategy would adjust the number of proteins being synthesized to the likelihood of them folding properly [48], thereby avoiding accumulation of misfolded proteins that can result in toxic protein aggregates [64].

DnaK’s association with ribosomes in bacteria facing cytoplasmic Mg^2+^ starvation is reminiscent of Hsp70’s behavior in eukaryotes, which lack TF. That is, the ribosome-associated Hsp70 Ssb—which, like DnaK, interacts with ribosomes via its C-terminus [65]—is responsible for both de novo protein folding [66] and feedback on translation [67,68] in eukaryotes. Ssb interacts with its cognate J-domain cochaperone zuotin and the Hsp70 Ssz; the latter 2 proteins form a conserved ribosome-associated complex (RAC) that binds the large 60S ribosomal subunit near the exit tunnel, recruits Ssb to nascent polypeptide chains, and stimulates ATP hydrolysis by Ssb, thus poising the complex to coordinate protein synthesis with protein folding [69,70]. By contrast, TF is largely responsible for cotranslational protein folding in bacteria except under particular stress conditions (e.g., cytoplasmic Mg^2+^ starvation) in which DnaK performs this function and is essential for survival (Fig 6).

Finally, the ribosome modulates folding of polypeptides tethered to its surface [71,72]. The ribosome–polypeptide interactions are mediated by the negative charge conferred by ribosomal RNA and negatively charged surface-exposed amino acids of ribosomal proteins [73] that are neutralized by Mg^2+^ ions [74]. The ribosome surface recognizes similar motifs in nascent polypeptide chains as TF [75]. Therefore, conditions that favor ribosome association with DnaK over TF, such as cytoplasmic Mg^2+^ limitation or inactivation of the *tig* gene, may aid folding of those proteins synthesized in response to a decrease in cytoplasmic Mg^2+^ or hyperosmotic stress, which *S*. Typhimurium experiences during infection [16,76].

## Materials and methods

### Bacterial strains and growth conditions

Bacterial strains, plasmids, and oligonucleotides used in this study are presented in S1 and S2 Tables. *S*. *enterica* serovar Typhimurium strains are derived from wild-type strain 14028s. Strains were constructed using λ Red-mediated recombination [77] and P22-mediated phage transduction. To minimize the appearance of spontaneous mutations in strains harboring mutations in the *dnaK*, *dnaJ*, *cbpA*, *djlA*, or *tig* genes, the mutations (genetically marked with an antibiotic resistance marker) were regularly transduced into a fresh wild-type 14028s background and propagated at 30°C during strain construction.

*S*. Typhimurium strains were grown in N-minimal medium (pH 7.7) supplemented with 0.1% casamino acids, 38 mM glycerol, and the indicated concentrations of MgCl_2_. Unless otherwise indicated, bacteria were grown at 37°C in a water bath with 250 rpm shaking.

Plasmids were generated by Gibson assembly, confirmed by Sanger sequencing, and introduced by electroporation into *S*. Typhimurium strains. IPTG concentrations used for plasmid induction in physiology experiments were 100 μM IPTG for pDnaK, pDnaK(T199A), and pDnaK(1–563), and 250 μM for pMgtC. Ampicillin for plasmid maintenance was used at 50 μg/ml in *S*. Typhimurium and 100 μg/ml in *E*. *coli*.

### Investigating in vivo association of DnaK and TF with ribosomes

Bacterial cultures were grown overnight in 10 mM Mg^2+^, washed 3 times in medium lacking Mg^2+^, and subcultured 1:50 in 200 ml of N-minimal medium containing 10 μM or 10 mM Mg^2+^ at 37°C with 250 rpm shaking. At the indicated times, the cultures were rapidly chilled by pouring into polypropylene-copolymer centrifuge bottles (Nalgene) containing ice slurry. Cells were pelleted for 20 min (12,000 rpm, 4°C) in a Evolution RC Superspeed centrifuge (Sorvall) using a SLA-3000 fixed angle rotor (Sorvall), washed 1 time with 25 ml wash buffer (20 mM Tris (pH 7.5), 100 mM NaCl, 10 mM MgCl_2_), and resuspended in 1 ml of ice-cold Buffer A (20 mM Tris (pH 7.5), 300 mM NH_4_Cl, 10 mM MgCl_2_, 0.5 mM EDTA, 6 mM β-mercaptoethanol) supplemented with 0.4 mg/ml lysozyme, 10 U/ml DNase I (New England Biolabs), and 10 U/ml SUPERase In RNAse inhibitor (Thermo Fisher Scientific). Following incubation on ice for 30 min, cells were further lysed by sonication at 4°C. The lysate was centrifuged at 4°C at 30,000×g for 30 min to obtain the clarified S30 fraction. The absorbance at 260 nm of the S30 fraction was measured with a Nanodrop using a 1:100 dilution. An equivalent of 60 A260 units in a total volume of 4 ml was layered on a 4-ml 38% sucrose cushion made with Tight-Couples Buffer (20 mM Tris-HCl (pH 7.5), 50 mM MgOAc, 100 mM NH_4_Cl, 1 mm DTT, 0.5 mM EDTA). The preparation was ultracentrifuged for 16 h (37,500 rpm, 4°C) using a 90 Ti rotor (Beckman Coulter). Following ultracentrifugation, the supernatant was removed, and the ribosomal pellet was resuspended in 100 μl of Tight-Couples Buffer and shaken into solution overnight at 4°C. The absorbance at 260 nm of the ribosomal fraction was measured using a Nanodrop instrument (NanoDrop Machines).

To confirm the amount of input protein present in clarified cell lysates, an aliquot of each S30 extract was normalized for equivalent RNA content (5.25 mg/ml RNA = 5 μl extract used) and probed by western blot. To determine the presence of ribosome-associated DnaK and TF in ribosomal fractions, an aliquot of each fraction was normalized for equivalent RNA content (20 mg/ml RNA = 15 μl fraction used), brought up to 15 μl in Tight-Couples Buffer, and combined with 5 μl of 4× Laemmli sample buffer. The samples were analyzed by western blotting using anti-DnaK, anti-TF, and anti-S1 antibodies. Total ribosomal protein staining was performed with SYPRO Ruby Protein Gel Stain (Thermo Fisher Scientific).

Clarified lysates for polysome profiling were obtained as described above. An equivalent of 60 A260 units in a total volume of 400 μl was layered on 10 ml 10% to 40% linear sucrose gradients made with Buffer A and ultracentrifuged for 3 h and 15 min (40,000 rpm, 4°C) using an SW40 Ti rotor (Beckman Coulter). Following ultracentrifugation, the tubes were mounted onto a density gradient fractionator (Brandel) and fractionation was performed using a Minipuls peristaltic pump (Gilson). The absorbance at 260 nm of each fraction 300 μl fraction was measured using a Nanodrop instrument (NanoDrop Machines) and plotted to obtain the in vivo polysome profile.

To determine the presence of ribosome-associated DnaK in sucrose fractions, 16.5 μl of each fraction was combined with 5.5 μl of 4× Laemmli sample buffer. The samples were analyzed by western blotting using anti-DnaK and anti-TF antibodies.

Dismantling of ribosomes was performed by sedimenting the lysate through a sucrose cushion or gradient supplemented with 10 mM EDTA.

The DnaK to ribosome ratio was estimated as follows: First, the number of molecules of DnaK in samples was compared to a standard curve of purified protein. Second, the number of ribosomes in the same samples was estimated based on RNA concentration measured by Nanodrop (Nanodrop Machines). Approximately 85% of total RNA was assumed to be ribosomal RNA (rRNA) [78]. Each ribosome was assumed to be 2.7 mDa in mass [79], and 60% of ribosomal mass was assumed to be made up of rRNA [79].

### Investigating in vivo protein synthesis

For ^35^S-methionine labeling, cells were grown overnight in 10 mM Mg^2+^, washed 3 times in medium lacking Mg^2+^, and subcultured 1:50 in 2 ml of N-minimal medium with 10 μM or 10 mM Mg^2+^. At the indicated times (5 h for low Mg^2+^ and 4 h for high Mg^2+^), 1 ml of the culture was transferred to a new tube for labeling. Each 1 ml of culture was labeled with 4.33 μCi of ^35^S-methionine (PerkinElmer). Following 1 h of ^35^S-methionine incorporation, 1 ml aliquots of each culture were added to microcentrifuge tubes pre-aliquoted with 200 μl ice-cold 50% trichloroacetic acid (TCA). (This moderately longer pulse labeling time was used to eliminate the need for growing bacteria in medium lacking methionine, which is necessary for short labeling times and would have caused cells to experience both Mg^2+^ starvation and methionine starvation as opposed to solely Mg^2+^ starvation as achieved in this study.) Proteins were precipitated overnight at 4°C.

Precipitated protein mixtures were collected by vacuum filtration on 25 mm glass microfiber filters, grade GF/C (Whatman) prewashed with 5 ml ice-cold 5% TCA. Filters were washed 2 times with 5 ml ice-cold 5% TCA and 1 time with 5 ml ice-cold ethanol. Filters were then removed and dried for at least 2 h at room temperature. Dried filters were soaked in 5 ml Ultima Gold scintillation fluid (PerkinElmer) for 15 min. Signal from each filter was measured using an LS 6500 liquid scintillation counter (Beckman Coulter). Scintillation values from a blank filter, which harbored minimal scintillation signal, were subtracted from sample readings. An unlabeled control with bacterial culture not pulse labeled with ^35^S-methionine was included in every experiment.

L-AHA labeling was performed as described [51]. Briefly, cells were grown in 2 ml N-minimal medium lacking casamino acids and instead supplemented with all individual amino acids except methionine. After 5.5 h growth, 2 μl of 40 mM L-AHA (Click Chemistry Tools) dissolved in DMSO was added to 2 ml cultures. Cultures were returned to the water bath and L-AHA incorporation was allowed to proceed for 30 min. Subsequently, cultures were rapidly chilled on ice, pelleted, and washed 3 times with an equivalent volume of ice-cold phosphate-buffered saline (PBS). Cell pellets were frozen and stored at −80°C. The pellets were thawed, resuspended in a buffer of 50 mM Tris (pH 8.0) with 0.5% sodium dodecyl sulfate (SDS), and lysed by sonication. Lysates were centrifuged for 10 min (30,000×*g*, 4°C) to separate insoluble components. The soluble lysate was transferred to a clean microcentrifuge tube and covalent attachment of tetramethylrhodamine azide (TAMRA) (Click Chemistry Tools) was carried out using the Click-&-Go Protein Reaction Buffer Kit (Click Chemistry Tools). Total protein was isolated by methanol–chloroform extraction and the protein pellet was washed 3 times with 450 μl methanol to remove unattached TAMRA. The washed pellet was resuspended in 200 μl of Laemmli sample buffer lacking β-mercaptoethanol. The total protein content of the solution was measured using a Micro BCA Protein Assay Kit (Thermo Fisher Scientific) and 7.5 μg of total protein per sample was electrophoresed on SDS-PAGE gels. Nascent protein amounts were analyzed by western blotting using anti-TAMRA antibodies (Novus Biologicals).

To investigate the presence of ^35^S-methionine-labeled proteins in insoluble and soluble cellular fractions, bacteria were grown in 2 ml low (10 μM) Mg^2+^ medium for 5 h and labeled with ^35^S-methionine for 1 h, and 1 ml aliquots of each culture were added to microcentrifuge tubes pre-aliquoted with 200 μl ice-cold 50% TCA. Following 10 min of incubation at 4°C, the samples were centrifuged at low speed (1,000×g, 4°C) to separate insoluble inclusion bodies. The supernatant was transferred to a fresh microcentrifuge tube, further acidified by addition of 200 μl of ice-cold 50% TCA, and incubated overnight at 4°C. The insoluble pellets were washed 1 time with 100 μl of PBS with 0.01% Triton to remove membrane proteins and resuspended in 750 μl PBS prior to loading onto filters. Proteins were collected on filters and radioactivity was quantified by scintillation counting as described above.

To investigate global protein stability, bacteria were grown for 5 h in low (10 μM) Mg^2+^ medium and labeled with ^35^S-methionine for 1 h. A 0.5 ml sample (denoted as pre-tetracycline) was collected, combined with 100 μl of ice-cold 50% TCA, and incubated overnight 4°C. ^35^S-methionine signal of the precipitated protein in this sample represents the starting pool of newly synthesized protein to be examined in the stability experiment. Tetracycline was added to the remaining culture at a final concentration of 62.5 μg/ml to halt translation and the fate of the protein pool was monitored; if proteins degrade, loss of ^35^S-methionine signal in future precipitated samples is expected. Following 18 h, a 0.5 ml post-tetracycline was collected as described above. Proteins were collected on filters and radioactivity was quantified by scintillation counting as described above. Protein amounts remaining following tetracycline treatment were calculated as the amount of ^35^S-methionine signal in the post-tetracycline sample divided by the ^35^S-methionine signal of the pre-tetracycline sample.

### Western blot analysis

Samples in Laemmli sample buffer were boiled at 95°C for 10 min. The indicated amount of each mixture was electrophoresed on NuPAGE 4% to 12% Bis-Tris gels (Thermo Fisher Scientific) and transferred to nitrocellulose membranes using the iBlot 2 transfer device (Thermo Fisher Scientific). Following transfer, membranes were blocked in 3% skim milk at room temperature for 2 h, incubated with the indicated primary antibodies for 1 h, washed 3 times, incubated with secondary antibodies (GE Healthcare and Promega) for 1 h, and washed 3 times. Rabbit anti-FLAG (Thermo Fisher Scientific) and rabbit anti-DnaK (Thermo Fisher Scientific) antibodies were used at 1:5,000 dilution in TBST. Mouse anti-TF (Takara Bio) antibodies were used at 1:50,000 dilution. Rabbit anti-S1 ribosomal protein (Agrisera) antibodies were used at 1:1,000 dilution. Horseradish peroxidase-conjugated anti-rabbit (GE Healthcare) and anti-mouse (Promega) secondary antibodies were used at 1:5,000 dilution. Chemiluminescent signal was developed with SuperSignal West Femto Maximum Sensitivity Substrate (Thermo Fisher Scientific) and captured with an ImageQuant LAS 4000 imager (Fujifilm).

### Protein purification

Overnight cultures of *E*. *coli* BL21(DE3) carrying the plasmids for recombinant protein expression (C-terminal His tags for DnaK, TF, DnaK(T199A), DnaK(1–563), CbpA, GrpE, and HtpG and N-terminal His tag for DnaJ; presented in S1 Table) were diluted 1:100 in 500 ml LB broth containing 100 μg/ml ampicillin. After 3 h of growth at 37°C with 250 rpm shaking, protein production was induced by addition of IPTG (1 mM). The temperature was lowered to 16°C and induction was allowed to proceed overnight with shaking at 150 rpm. The cultures were pelleted by centrifugation for 30 min (8,500 rpm, 4°C) in an Evolution RC Superspeed centrifuge (Sorvall) using an SLC-6000 fixed angle rotor (Sorvall). The pellet was resuspended in 15 ml of Buffer A (50 mM Tris (pH 8.0), 150 mM NaCl, 1 mM MgCl_2_) supplemented with 10 U/ml DNase I (New England Biolabs) and 150 μg/ml lysozyme. Following 30 min of incubation on ice, cells were further lysed by sonication. The lysate was clarified by ultracentrifugation for 30 min at 35,000 rpm in a 90 Ti rotor (Beckman Coulter).

The clarified lysate was applied to a column containing a 1 ml bed volume of Ni-NTA agarose (Qiagen) pre-equilibrated with Buffer B (50 mM Tris (pH 8.0), 150 mM NaCl). The agarose beads were washed 8 times with 8 ml of Buffer B to remove unbound proteins. Captured proteins were eluted 4 times, each with 1 ml Buffer C (50 mM Tris (pH 8.0), 150 mM NaCl, 250 mM imidazole). Following SDS-PAGE and Coomassie blue staining analysis of all fractions obtained throughout the purification process, the eluates were pooled, buffer exchanged with Buffer D (50 mM Tris (pH 8.0), 150 mM NaCl, 10% glycerol), and concentrated using Amicon Ultra-15 Centrifugal Filter Units (Millipore).

Protein concentration was determined using a Nanodrop (Nanodrop Machines) and confirmed using a Micro BCA Protein Assay Kit (Thermo Fisher Scientific). Proteins were stored at −80°C long-term, thawed on ice when ready to use, subsequently stored at 4°C, and used within 2 weeks of thawing.

### Investigating DnaK association with ribosomes in vitro

In vitro protein synthesis reactions using the PURExpress system (New England Biolabs) based on components from *E*. *coli*, a species closely related to *S*. Typhimurium, were set up in 10 μl reaction volumes and supplemented with 1 U SUPERase-In RNase inhibitor (Thermo Fisher Scientific). Purified DnaK or TF was added at a final concentration of 5 μM. A total of 500 ng of plasmid template pDHFR supplied in the PURExpress kit was added as the last component to initiate protein synthesis. The reactions were incubated at 37°C for 1 h to allow protein synthesis to proceed. After the 1 h incubation, the reactions were immediately placed on ice to halt protein synthesis, and 140 μl of TAKM7 buffer (50 mM Tris-HCl (pH 7.5), 70 mM NH_4_Cl, 30 mM KCl) with either 10 mM or no MgCl_2_ was added to bring the volume to 150 μl. A 2.5 μl sample was reserved for analysis of the input sample.

The reaction combined with TAKM7 buffer was layered onto 400 μl of a 10% sucrose cushion made with TAKM7 buffer with either 10 mM or 0 MgCl_2_ in 1 ml open-top thick wall polypropylene tubes (Beckman Coulter), bringing the final sample Mg^2+^ concentration to 10 mM or 0.16 mM, respectively. Samples were ultracentrifuged for 35 min at 70,000 rpm, 4°C in a TLA-120.2 rotor (Beckman Coulter) to separate ribosomes from other components.

Following sedimentation by ultracentrifugation, the supernatant was removed and saved. Ribosome pellets were resuspended in 25 μl TAKM7 buffer. The RNA concentration of the resuspended ribosomes was measured using a Nanodrop instrument (Nanodrop Machines). Non ribosome-bound supernatants (10 μl) and normalized ribosome samples (100 ng/μl RNA = 20 μl sample loaded) were electrophoresed on NuPAGE 4% to 12% Bis-Tris gels (Thermo Fisher Scientific) and transferred to a nitrocellulose membrane as described above. The blot was developed with Revert 520 Total Protein Stain (LI-COR Biosciences), followed by probing with anti-DnaK (Thermo Fisher Scientific) or anti-TF (Takara Bio) antibodies.

To investigate ribosome association of full-length and truncated DnaK, 10 μl PURExpress reactions were set up as described above. Purified DnaK or its truncated form, DnaK(1–563), was added at a final concentration of 5 μM. Following 1 h of protein synthesis at 37°C, 140 μl TAKM7 buffer (50 mM Tris-HCl (pH 7.5), 70 mM NH_4_Cl, 30 mM KCl, 7 mM MgCl_2_) was added to bring sample volumes to 150 μl. Samples were ultracentrifuged, electrophoresed, and transferred to nitrocellulose membranes as described above, followed by probing with anti-DnaK (Thermo Fisher Scientific) antibodies.

Samples lacking purified DnaK or TF were included as negative controls.

### In vitro protein synthesis assay

DNA templates were prepared by PCR amplification of wild-type *S*. Typhimurium genomic DNA using primer pairs presented in S2 Table, which contain the requisite T7 promoter and terminator sequences for use with the PURExpress system.

In vitro protein synthesis using the PURExpress system (New England Biolabs) was carried out following the manufacturer’s instructions. Where indicated, purified proteins were added to the following concentrations: DnaK or DnaK(T199A) (5 μM), HtpG (5 μM), or TF (5 μM). An equivalent volume of empty buffer was added to controls lacking added purified protein. Reactions were initiated by addition of 250 ng DNA template and incubated at 37°C in a thermomixer with 300 rpm agitation. Following 45 min of incubation, 1 μl aliquots were removed and immediately quenched with pre-aliquoted Laemmli sample buffer (Bio-Rad) to a final volume of 10 μl (Bio-Rad). Samples were analyzed by western blotting using anti-FLAG antibodies.

To investigate in vitro protein synthesis supplied with mRNA template, mRNA corresponding to the appropriate gene was transcribed in vitro using T7 RNA polymerase (New England Biolabs) at 37°C for 1 h. The reaction was treated with DNase I digestion (New England Biolabs), purified by ethanol precipitation, and resuspended in nuclease-free water. In vitro protein synthesis was performed as described using 2 μg mRNA template in place of 250 ng DNA template.

To investigate in vitro protein synthesis in the presence of purified DnaK and TF, the reaction was carried out as described above following preincubation of DnaK or TF with the protein synthesis machinery (PURExpress Solutions A and B) for 30 min at room temperature. Following preincubation, the second chaperone was added along with DNA template. The reaction was allowed to proceed for 45 min and reporter protein yield was assessed by western blot, both as described above.

### ATP hydrolysis assay

Release of inorganic phosphate (Pi) from ATP hydrolysis was measured using the EnzChek Phosphate Assay kit (Thermo Fisher Scientific) according to the manufacturer’s instructions, with the exception that reactions were scaled down to 100 μl and performed in clear-bottom 96-well plates. Where indicated, purified proteins were added to the following final concentrations: DnaK (2 μM), DnaJ (0.4 μM), CbpA (0.4 μM), or GrpE (0.2 μM). 70S ribosomes (New England Biolabs) or 30S or 50S ribosomal subunits were added to a concentration of 0.5 μM. Following preincubation at room temperature for 10 min, 4 μl of 10 mM ATP was added to initiate the reaction. Reactions proceeded at 22°C. Absorbance at 360 nm, which reflects the production of 2-amino-6-mercapto-7-methylpurine stimulated by the presence of inorganic Pi [80] (the product of the ATPase reaction), was recorded every 5 min using a Spectramax 340PC-384 plate reader. The A360 value at t = 0 was subtracted from subsequent measurements. Controls reactions performed without the addition of ATP confirmed that no contaminating Pi was present in reagents and labware.

### Insoluble protein isolation and analysis

Bacterial cultures were grown overnight in 10 mM Mg^2+^, washed 3 times in medium lacking Mg^2+^, and subcultured 1:50 in 10 ml of low or high Mg^2+^ medium for the indicated times. Insoluble protein isolation was performed as described [81]. Total, soluble, and insoluble protein samples were electrophoresed on NuPAGE 4% to 12% Bis-Tris gels (Thermo Fisher Scientific) and stained with Coomassie blue. Quantification of protein amounts was performed using the gel analysis feature of ImageJ to measure intensities and abundances of protein bands present in each lane.

### Bacterial survival assay

Bacterial cultures were grown overnight in 10 mM Mg^2+^, washed 3 times in medium lacking Mg^2+^, and subcultured 1:50 in 2 ml N-minimal medium with 10 μM or 10 mM Mg^2+^. For hyperosmotic stress conditions, an overnight culture grown in medium with high (10 mM MgCl_2_) was washed 3 times in medium with 1 mM MgCl_2_ and diluted 1:50 into LB medium with 1 M NaCl and 1 mM MgCl_2._ After 24 h of growth, cells were serially diluted in sterile PBS and plated on LB agar plates. After overnight incubation at 37°C, the number of colony forming units (CFUs) was enumerated and corrected for the dilution factor and plating volume to calculate the number of CFUs in the culture.

### Sublethal chemical translation inhibitor assay

Overnight bacterial cultures grown in high Mg^2+^ were washed 3 times in medium lacking Mg^2+^ as described. Upon subculture into 2 ml low Mg^2+^ medium at a 1:50 dilution, antibiotics were added to the concentrations indicated above. Following 24 h of growth, the cultures were serially diluted in sterile PBS and plated on LB agar plates.

### Luciferase reactivation

Luciferase reactivation was performed as described [82,83] with slight modifications and scaled down to a 40 μl reaction volume. Briefly, 80 nM firefly luciferase (Sigma Aldrich) was denatured at 42°C for 5 min in a buffer of 25 mM HEPES (pH 7.5), 50 mM KCl, 15 mM MgCl_2_, and 2 mM DTT, then placed on ice. BSA (0.05 mg/ml), ATP (1 mM), and an ATP regeneration system (20 mM creatine phosphate and 0.06 mg/ml creatine kinase) were added to the reaction mix, followed by addition of chaperones, cochaperones, and/or ribosomes. DnaK, CbpA, and GrpE were used at concentrations of 2 μM, 0.4 μM, and 0.2 μM, respectively, and 70S ribosomes were used at a concentration of 0.5 μM.

Renaturation of luciferase was allowed to proceed at 30°C. At the indicated times, 5 μl aliquots were removed. Luciferase activity was determined with the addition of 120 μl of substrate containing 200 μM D-luciferin (Sigma Aldrich), 0.5 mM ATP, and 10 mM MgCl_2_. Luminescence was measured in a Spark Multimode plate reader (TECAN) with an integration time of 10,000 msec. The luminescence value at t = 0 was subtracted from subsequent measurements to determine the amount of luminescent activity regained.

### Nuclease activity assay

To rule out the presence of DNases or RNases in our preparations of purified DnaK protein, DNA and mRNA corresponding to *pmrA*-FLAG (the same template used in in vitro protein synthesis assays) were produced by PCR and T7-mediated in vitro transcription, respectively. The resulting DNA and mRNA fragments were purified using a QIAquick PCR purification kit (Qiagen) and ethanol precipitation, respectively. Stability reactions were performed in a buffer of 25 mM Tris (pH 8.0), 100 mM KCl, 12 mM MgCl_2_, 2 mM ATP, 4 mM phosphoenolpyruvate, and 20 μg/ml pyruvate kinase in the presence of either empty protein storage buffer or 5 μM DnaK, and 250 ng DNA substrate or 1 μg RNA substrate was added to a reaction volume of 10 μl. Samples were incubated at 37°C. At the indicated times, 4 μl aliquots (for DNA) or 3.5 μl aliquots (RNA) were removed and frozen in dry ice. Aliquots were combined with 6X DNA loading dye (Thermo Fisher Scientific) and electrophoresed on 1% Tris-acetate-EDTA agarose gels, then stained with SYBR Gold nucleic acid gel stain (Thermo Fisher Scientific) and visualized in an Amersham ImageQuant 800 imager (Cytiva).

### Raw data accessibility

Images of uncropped gels, strain verification, additional replicates, quantifications, and all other raw data can be found in: DOI: 10.17632/m7788yc9sf.1.

## Supporting information

S1 Fig DnaK associates with intact ribosomes and not with dismantled ribosomes. **(A, B)** Polysome profile analysis of wild-type (14028s) *S*. Typhimurium following 5 h of growth in low (10 μM) Mg^2+^ or 4.5 h of growth in high (10 mM) Mg^2+^ and western blot analysis of the corresponding fractions. Blot was developed using antibodies recognizing DnaK and TF. **(C)** Western blot analysis of clarified cell lysates (left) and ultracentrifuged fractions (right) of wild-type (14028s) *S*. Typhimurium following 5 h of growth in low (10 μM) Mg^2+^ using a sucrose cushion treated with EDTA. Blot was developed using antibodies recognizing DnaK or the ribosomal protein control S1. **(D)** Polysome profile analysis of wild-type *S*. Typhimurium lysate treated with EDTA and RNase A to fully dismantle ribosomes. Shown in **(A and B)** and **(C and D)** are the representatives of 3 and 2 independent biological replicates, respectively.
(TIF)

S2 Fig *dnak14* mutant harbors a transposon insertion in the *dnaK* coding region and specifies a truncated DnaK protein. **(A)** Western blot of whole cell extract from wild-type (14028s) and *dnaK14* (CC186) *S*. Typhimurium following 5 h of growth in low (10 μM) Mg^2+^. Blot was developed with polyclonal antibodies directed to DnaK. **(B)** Schematic of *dnaK* gene in mutant *dnaK14*. Transposon Tn10*d*Cm inserted immediately after nucleotide 1691 in the *dnaK* coding region. In blue are nucleotides corresponding to the *dnaK* coding region near the site of Tn*10*dCm insertion. In red are nucleotides corresponding to transposon Tn*10*dCm, including the inverted repeat originating from transposon Tn*10*. In capital letters is the TGA stop codon in frame with the *dnaK* coding region. **(C)** Schematic of the domain architecture of wild-type DnaK protein. Transposon Tn*10*dCm in mutant *dnaK14* provides an early stop codon in frame that results in the production of a truncated DnaK protein. **(D)** ATP hydrolysis in the presence or absence of purified full-length or truncated DnaK (2 μM) alone or in combination with cochaperone DnaJ (0.4 μM) and nucleotide exchange factor GrpE (0.2 μM). Shown in **(A)** is the representative of 4 independent biological replicates. Data in **(D)** represent mean ± SD of 3 independent assays performed in buffer containing 20 mM Mg^2+^. The numerical values underlying this figure can be found in S1 Data.
(TIF)

S3 Fig Preparations of DnaK protein are pure. **(A)** Coomassie blue staining of DnaK protein following recombinant expression in *E*. *coli* BL21(DE3) and purification. **(B, C)** Stability of *pmrA-*FLAG DNA in the presence of purified DnaK (5 μM). **(D, E)** Stability of *pmrA-*FLAG mRNA in the presence of purified DnaK (5 μM). **(F)** Quantification of in vitro synthesized PmrA-FLAG protein in the presence of varying concentrations of purified DnaK. Data represent mean ± SD of 3 independent assays performed in buffer containing 9 mM Mg^2+^. The numerical values underlying this figure can be found in S1 Data.
(TIF)

S4 Fig DnaK does not refold heat-denatured luciferase when incubated in the presence of ribosomes. **(A)** Reactivation of heat-denatured luciferase in the presence or absence of purified DnaK (2 μM) alone or in combination with cochaperones (CbpA [0.4 μM] and GrpE [0.2 μM]) or 70S ribosomes (0.5 μM). Data represent mean ± SD of 3 independent assays. The numerical values underlying this figure can be found in S1 Data.
(TIF)

S5 Fig The *dnaK14*, *dnaJ*, and *tig* mutants have similar viability during growth in high Mg^2+^. **(A)** Survival of wild-type (14028s) and *dnaK14* (CC186) *S*. Typhimurium harboring the plasmid vector (pUHE-21-2-*lacI*^*q*^) or *dnaK-*expressing plasmid (pDnaK) following 24 h in high (10 mM) Mg^2+^. **(B)** Survival of wild-type (14028s), *dnaK14* (CC186), *dnaJ* (EG16309), and *tig* (CC361) *S*. Typhimurium following 24 h in high (10 mM) Mg^2+^. Data represent mean ± SD of 2 independent biological replicates in **(A)** and 3 independent biological replicates in **(B)**. Statistical analysis was performed using two-tailed Student’s *t* test comparing the indicated sample group to the wild-type sample group (ns = not significant). The numerical values underlying this figure can be found in S1 Data.
(TIF)

S6 Fig Transcription of a plasmid-borne copy of the *mgtC* gene from a heterologous promoter reduces protein synthesis. **(A)** ^35^S-methionine labeling of wild-type (14028s) and *dnaK14* (CC186) *S*. Typhimurium harboring the plasmid vector (pUHE-21-2-*lacI*^*q*^) or *mgtC*-expressing plasmid (pMgtC) following 5 h of growth in low (10 μM) Mg^2+^. Data represent mean ± SD of 3 independent biological replicates. The numerical values underlying this figure can be found in S1 Data.
(TIF)

S1 Table Strains and plasmids used in this study. (DOCX)

S2 Table Oligonucleotides used in this study. (DOCX)

S1 Data Individual numerical values corresponding to data presented in figures. (XLSX)

S2 Data Strain verification and raw images underlying data presented in the text. (PDF)

## Abbreviations

ADP: adenosine diphosphate
ATP: adenosine triphosphate
CFU: colony forming unit
NBD: nucleotide-binding domain
PBS: phosphate-buffered saline
RAC: ribosome-associated complex
rRNA: ribosomal RNA
SBD: substrate-binding domain
SDS: sodium dodecyl sulfate
TCA: trichloroacetic acid
TF: trigger factor

## References

[1] Agashe VR, Guha S, Chang H-C, Genevaux P, Hayer-Hartl M, Stemp M, et al. Function of Trigger Factor and DnaK in multidomain protein folding: increase in yield at the expense of folding speed. Cell. 2004;117(2):199–209. doi: 10.1016/s0092-8674(04)00299-5 . 15084258

[2] Kampinga H. Chaperones in preventing protein denaturation in living cells and protecting against cellular stress. Handb Exp Pharmacol. 2006;(172). doi: 10.1007/3-540-29717-0_1 . 16610353

[3] Wild J, Altman E, Yura T, Gross C. DnaK and DnaJ heat shock proteins participate in protein export in *Escherichia coli*. Genes Dev. 1992;6(7). doi: 10.1101/gad.6.7.1165 . 1628824

[4] Imamoglu R, Balchin D, Hayer-Hartl M, Hartl FU. Bacterial Hsp70 resolves misfolded states and accelerates productive folding of a multi-domain protein. Nat Commun. 2020;11(1):1–13. doi: 10.1038/s41467-019-14245-4 31953415 PMC6969021

[5] MYu S, Goldberg A. Involvement of the chaperonin dnaK in the rapid degradation of a mutant protein in Escherichia coli. EMBO J. 1992;11(1). doi: 10.1002/j.1460-2075.1992.tb05029.x . 1740117 PMC556427

[6] Hartl FU. Molecular chaperones in cellular protein folding. Nature. 1996;381(6583):571–580. doi: 10.1038/381571a0 8637592

[7] Laufen T, Mayer M, Beisel C, Klostermeier D, Mogk A, Reinstein J, et al. Mechanism of regulation of Hsp70 chaperones by DnaJ cochaperones. Proc Natl Acad Sci U S A. 1999;96(10):5452–5457. doi: 10.1073/pnas.96.10.5452 . 10318904 PMC21880

[8] Liberek K, Marszalek J, Ang D, Georgopoulos C, Zylicz M. *Escherichia coli* DnaJ and GrpE heat shock proteins jointly stimulate ATPase activity of DnaK. Proc Natl Acad Sci U S A. 1991;88(7). doi: 10.1073/pnas.88.7.2874 . 1826368 PMC51342

[9] Turturici G, Sconzo G, Geraci F. Hsp70 and its molecular role in nervous system diseases. Biochem Res Int. 2011;2011. doi: 10.1155/2011/618127 . 21403864 PMC3049350

[10] Murphy M. The HSP70 family and cancer. Carcinogenesis. 2013;34(6). doi: 10.1093/carcin/bgt111 . 23563090 PMC3670260

[11] Snoeckx L, Cornelussen R, Van Nieuwenhoven F, Reneman R, Van Der Vusse G. Heat shock proteins and cardiovascular pathophysiology. Physiol Rev. 2001;81(4). doi: 10.1152/physrev.2001.81.4.1461 . 11581494

[12] Bukau B, Walker G. Cellular defects caused by deletion of the *Escherichia coli dnaK* gene indicate roles for heat shock protein in normal metabolism. J Bacteriol. 1989;171(5). doi: 10.1128/jb.171.5.2337–2346.1989 . 2651398 PMC209906

[13] Takaya A, Tomoyasu T, Matsui H, Yamamoto T. The DnaK/DnaJ chaperone machinery of *Salmonella enterica* serovar Typhimurium is essential for invasion of epithelial cells and survival within macrophages, leading to systemic infection. Infect Immun. 2004;72(3). doi: 10.1128/IAI.72.3.1364–1373.2004 . 14977940 PMC356005

[14] Liberek K, Galitski T, Zylicz M, Georgopoulos C. The DnaK chaperone modulates the heat shock response of *Escherichia coli* by binding to the sigma 32 transcription factor. Proc Natl Acad Sci U S A. 1992;89(8). doi: 10.1073/pnas.89.8.3516 . 1565647 PMC48899

[15] Calloni G, Chen T, Schermann SM, Chang HC, Genevaux P, Agostini F, et al. DnaK functions as a central hub in the E. coli chaperone network. Cell Rep. 2012;1(3):251–264. doi: 10.1016/j.celrep.2011.12.007 . 22832197

[16] Cunrath O, Bumann D. Host resistance factor SLC11A1 restricts *Salmonella* growth through magnesium deprivation. Science. 2019;366(6468):995–9. Epub 2019/11/23. doi: 10.1126/science.aax7898 . 31753999

[17] Hoffmann A, Bukau B, Kramer G. Structure and function of the molecular chaperone Trigger Factor. Biochim Biophys Acta. 2010;1803(6):650–661. doi: 10.1016/j.bbamcr.2010.01.017 . 20132842

[18] Ferbitz L, Maier T, Patzelt H, Bukau B, Deuerling E, Ban N. Trigger factor in complex with the ribosome forms a molecular cradle for nascent proteins. Nature. 2004;431(7008):590–596. doi: 10.1038/nature02899 15334087

[19] Vabulas RM, Raychaudhuri S, Hayer-Hartl M, Hartl FU. Protein folding in the cytoplasm and the heat shock response. Cold Spring Harb Perspect Biol. 2010;2(12). doi: 10.1101/cshperspect.a004390 . 21123396 PMC2982175

[20] Bhandari V, Houry WA. Substrate interaction networks of the *Escherichia coli* chaperones: Trigger Factor, DnaK and GroEL. Adv Exp Med Biol. 2015;883:271–294. doi: 10.1007/978-3-319-23603-2_15 . 26621473

[21] Deuerling E, Schulze-Specking A, Tomoyasu T, Mogk A, Bukau B. Trigger factor and DnaK cooperate in folding of newly synthesized proteins. Nature. 1999;400(6745):693–696. doi: 10.1038/23301 10458167

[22] Teter S, Houry W, Ang D, Tradler T, Rockabrand D, Fischer G, et al. Polypeptide flux through bacterial Hsp70: DnaK cooperates with trigger factor in chaperoning nascent chains. Cell. 1999;97(6). doi: 10.1016/s0092-8674(00)80787-4 . 10380927

[23] Deuerling E, Patzelt H, Vorderwülbecke S, Rauch T, Kramer G, Schaffitzel E, et al. Trigger Factor and DnaK possess overlapping substrate pools and binding specificities. Mol Microbiol. 2003;47(5). doi: 10.1046/j.1365-2958.2003.03370.x . 12603737

[24] Buchmeier NA, Heffron F. Induction of *Salmonella* stress proteins upon infection of macrophages. Science. 1990;248(4956):730–732. 1970672 10.1126/science.1970672

[25] Yeom J, Groisman EA. Reduced ATP-dependent proteolysis of functional proteins during nutrient limitation speeds the return of microbes to a growth state. Sci Signal. 2021;14:2021. doi: 10.1126/scisignal.abc4235 33500334 PMC8378506

[26] Goloubinoff P, Sassi A, Fauvet B, Barducci A, De Los RP. Chaperones convert the energy from ATP into the nonequilibrium stabilization of native proteins. Nat Chem Biol. 2018;14(4):388–395. doi: 10.1038/s41589-018-0013-8 . 29507388

[27] Pu Y, Li Y, Jin X, Tian T, Ma Q, Zhao Z, et al. ATP-dependent dynamic protein aggregation regulates bacterial dormancy depth critical for antibiotic tolerance. Mol Cell. 2019;73(1):143–156. doi: 10.1016/j.molcel.2018.10.022 . 30472191

[28] Paek KH, Walker GC. Escherichia coli dnaK null mutants are inviable at high temperature. J Bacteriol. 1987;169(1):283–290. doi: 10.1128/jb.169.1.283-290.1987 3025174 PMC211765

[29] Wolska KI, Bugajska E, Jurkiewicz D, Kuć M, Jóźwik A. Antibiotic Susceptibility of *Escherichia coli dnaK* and *dnaJ* Mutants. Microb Drug Resist. 2000;6(2):119–126. doi: 10.1089/107662900419429 10990266

[30] Groisman EA, Cromie MJ, Shi Y, Latifi T. A Mg2+-responding RNA that controls the expression of a Mg2+ transporter. Cold Spring Harb Symp Quant Biol. 2006;71:251–258. doi: 10.1101/sqb.2006.71.005 . 17381304

[31] Lee EJ, Pontes MH, Groisman EA. A bacterial virulence protein promotes pathogenicity by inhibiting the bacterium’s own F1Fo ATP synthase. Cell. 2013;154(1):146–156. doi: 10.1016/j.cell.2013.06.004 ; PubMed Central PMCID: PMC3736803. 23827679 PMC3736803

[32] Shimizu Y, Inoue A, Tomari Y, Suzuki T, Yokogawa T, Nishikawa K, et al. Cell-free translation reconstituted with purified components. Nat Biotechnol. 2001;19(8):751–755. doi: 10.1038/90802 . 11479568

[33] Kang P, Craig E. Identification and characterization of a new Escherichia coli gene that is a dosage-dependent suppressor of a dnaK deletion mutation. J Bacteriol. 1990;172(4). doi: 10.1128/jb.172.4.2055-2064.1990 . 2180916 PMC208704

[34] Clerico E, Meng W, Pozhidaeva A, Bhasne K, Petridis C, Gierasch L. Hsp70 molecular chaperones: multifunctional allosteric holding and unfolding machines. Biochem J. 2019;476(11). doi: 10.1042/BCJ20170380 . 31201219 PMC7219557

[35] Saito H, Uchida H. Organization and expression of the *dnaJ* and *dnaK* genes of *Escherichia coli* K12. Mol Gen Genet. 1978;164(1):1–8. doi: 10.1007/BF00267592 360036

[36] Bardwell JC, Tilly K, Craig E, King J, Zylicz M, Georgopoulos C. The nucleotide sequence of the *Escherichia coli* K12 *dnaJ*+ gene. A gene that encodes a heat shock protein. J Biol Chem. 1986;261(4):1782–1785. doi: 10.1016/S0021-9258(17)36008-8 . 3003085

[37] Gur E, Biran D, Shechter N, Genevaux P, Georgopoulos C, Ron E. The Escherichia coli DjlA and CbpA proteins can substitute for DnaJ in DnaK-mediated protein disaggregation. J Bacteriol. 2004;186(21). doi: 10.1128/JB.186.21.7236-7242.2004 . 15489435 PMC523209

[38] Adell M, Calisto BM, Fita I, Martinelli L. The nucleotide-bound/substrate-bound conformation of the Mycoplasma genitalium DnaK chaperone. Protein Sci. 2018:27(5). doi: 10.1002/pro.3401 . 29520883 PMC5916121

[39] Bijlsma JJ, Groisman EA. The PhoP/PhoQ system controls the intramacrophage type three secretion system of *Salmonella enterica*. Mol Microbiol. 2005;57(1):85–96. Epub 2005/06/14. MMI4668 [pii] doi: 10.1111/j.1365-2958.2005.04668.x . 15948951

[40] Soncini FC, García Véscovi E, Solomon F, Groisman EA. Molecular basis of the magnesium deprivation response in *Salmonella typhimurium*: identification of PhoP-regulated genes. J Bacteriol. 1996;178(17):5092–5099. 8752324 10.1128/jb.178.17.5092-5099.1996 PMC178303

[41] Wickner S, Nguyen T, Genest O. The bacterial Hsp90 chaperone: cellular functions and mechanism of action. Ann Rev Microbiol. 2021;75:719–739. doi: 10.1146/annurev-micro-032421-035644 . 34375543

[42] Suh W, Burkholder W, Lu C, Zhao X, Gottesman M, Gross C. Interaction of the Hsp70 molecular chaperone, DnaK, with its cochaperone DnaJ. Proc Natl Acad Sci U S A. 1998;95(26). doi: 10.1073/pnas.95.26.15223 . 9860950 PMC28024

[43] Mayer M, Bukau B. Hsp70 chaperones: cellular functions and molecular mechanism. Cell Mol Life Sci. 2005;62(6):670–684. doi: 10.1007/s00018-004-4464-6 . 15770419 PMC2773841

[44] Bukau B, Horwich AL. The Hsp70 and Hsp60 chaperone machines. Cell. 1998;92(3):351–366. doi: 10.1016/s0092-8674(00)80928-9 9476895

[45] McCarty J, Walker G. DnaK as a thermometer: threonine-199 is site of autophosphorylation and is critical for ATPase activity. Proc Natl Acad Sci U S A. 1991;88(21):9513–9517. doi: 10.1073/pnas.88.21.9513 . 1835085 PMC52748

[46] Barthel T, Zhang J, Walker G. ATPase-defective derivatives of *Escherichia coli* DnaK that behave differently with respect to ATP-induced conformational change and peptide release. J Bacteriol. 2001;183(19). doi: 10.1128/JB.183.19.5482–5490.2001 . 11544208 PMC95437

[47] Buchberger A, Theyssen H, Schröder H, McCarty J, Virgallita G, Milkereit P, et al. Nucleotide-induced conformational changes in the ATPase and substrate binding domains of the DnaK chaperone provide evidence for interdomain communication. J Biol Chem. 1995:270(28). doi: 10.1074/jbc.270.28.16903 . 7622507

[48] Liu B, Han Y, Qian S. Cotranslational response to proteotoxic stress by elongation pausing of ribosomes. Mol Cell. 2013;49(3). doi: 10.1016/j.molcel.2012.12.001 . 23290916 PMC3570626

[49] Rodríguez-Galán O, García-Gómez J, Rosado I, Wei W, Méndez-Godoy A, Pillet B, et al. A functional connection between translation elongation and protein folding at the ribosome exit tunnel in Saccharomyces cerevisiae. Nucleic Acids Res. 2021;49(1). doi: 10.1093/nar/gkaa1200 . 33330942 PMC7797049

[50] Shalgi R, Hurt J, Krykbaeva I, Taipale M, Lindquist S, Burge C. Widespread regulation of translation by elongation pausing in heat shock. Mol Cell. 2013;49(3). doi: 10.1016/j.molcel.2012.11.028 . 23290915 PMC3570722

[51] Pontes MH, Yeom J, Groisman EA. Reducing ribosome biosynthesis promotes translation during low Mg2+ stress. Mol Cell. 2016;64(3):480–492. doi: 10.1016/j.molcel.2016.05.008 . 27746019 PMC5500012

[52] Groisman EA, Chan C. Cellular Adaptations to Cytoplasmic Mg^2+^ Limitation. Ann Rev Microbiol. 2021;75:649–672. doi: 10.1146/annurev-micro-020518-115606 34623895

[53] Wendel B, Pi H, Krüger L, Herzberg C, Stülke J, Helmann J. A central role for magnesium homeostasis during adaptation to osmotic stress. MBio. 2022;13(1). doi: 10.1128/mbio.00092-22 . 35164567 PMC8844918

[54] Epstein W. Osmoregulation by potassium transport in *Escherichia coli*. FEMS Microbiol Lett. 1986;39:73–78.

[55] Groisman EA, Hollands K, Kriner MA, Lee EJ, Park SY, Pontes MH. Bacterial Mg^2+^ homeostasis, transport, and virulence. Annu Rev Genet. 2013;47:625–646. doi: 10.1146/annurev-genet-051313-051025 ; PubMed Central PMCID: PMC4059682. 24079267 PMC4059682

[56] Daugaard M, Rohde M, Jäättelä M. The heat shock protein 70 family: Highly homologous proteins with overlapping and distinct functions. FEBS Lett. 2007;581(19). doi: 10.1016/j.febslet.2007.05.039 . 17544402

[57] Deuerling E, Gamerdinger M, Kreft SG. Chaperone interactions at the ribosome. Cold Spring Harb Perspect Biol. 2019. doi: 10.1101/cshperspect.a033977 30833456 PMC6824243

[58] Siller E, DeZwaan D, Anderson J, Freeman B, Barral J. Slowing bacterial translation speed enhances eukaryotic protein folding efficiency. J Mol Biol. 2010;396(5). doi: 10.1016/j.jmb.2009.12.042 . 20043920

[59] Hsu S-TD, Fucini P, Cabrita LD, Launay H, Dobson CM, Christodoulou J. Structure and dynamics of a ribosome-bound nascent chain by NMR spectroscopy. Proc Natl Acad Sci U S A. 2007. doi: 10.1073/pnas.0704664104 17940046 PMC2034214

[60] Fauvet B, Rebeaud M, Tiwari S, De Los RP, Goloubinoff P. Repair or degrade: the thermodynamic dilemma of cellular protein quality-control. Front Mol Biosci. 2021:8. doi: 10.3389/fmolb.2021.768888 . 34778379 PMC8578701

[61] Stouthamer AH. A theoretical study on the amount of ATP required for synthesis of microbial cell material. Antonie Van Leeuwenhoek. 1973;39(3):545–65. Epub 1973/01/01. doi: 10.1007/BF02578899 . 4148026

[62] Smock RG, Blackburn ME, Gierasch LM. Conserved, disordered C terminus of DnaK enhances cellular survival upon stress and DnaK in vitro chaperone activity. J Biol Chem. 2011;286(36). doi: 10.1074/jbc.M111.265835 . 21768118 PMC3173061

[63] Mogk A, Tomoyasu T, Goloubinoff P, Rüdiger S, Röder D, Langen H, et al. Identification of thermolabile *Escherichia coli* proteins: prevention and reversion of aggregation by DnaK and ClpB. EMBO J. 1999;18(24). doi: 10.1093/emboj/18.24.6934 . 10601016 PMC1171757

[64] Mogk A, Bukau B, Kampinga H. Cellular handling of protein aggregates by disaggregation machines. Mol Cell. 2018;69(2). doi: 10.1016/j.molcel.2018.01.004 . 29351843

[65] Hanebuth MA, Kityk R, Fries SJ, Jain A, Kriel A, Albanese V, et al. Multivalent contacts of the Hsp70 Ssb contribute to its architecture on ribosomes and nascent chain interaction. Nat Commun. 2016;7(1):1–13. doi: 10.1038/ncomms13695 27917864 PMC5150220

[66] Zhang Y, Valentín Gesé G, Conz C, Lapouge K, Kopp J, Wölfle T, et al. The ribosome-associated complex RAC serves in a relay that directs nascent chains to Ssb. Nat Commun. 2020;11(1):1504. doi: 10.1038/s41467-020-15313-w 32198371 PMC7083937

[67] Rakwalska M, Rospert S. The ribosome-bound chaperones RAC and Ssb1/2p are required for accurate translation in *Saccharomyces cerevisiae*. Mol Cell Biol. 2004;24(20):9186–9197. doi: 10.1128/MCB.24.20.9186–9197.2004 . 15456889 PMC517888

[68] Muldoon-Jacobs K, Dinman J. Specific effects of ribosome-tethered molecular chaperones on programmed -1 ribosomal frameshifting. Eukaryot Cell. 2006;5(4):762–770. doi: 10.1128/EC.5.4.762-770.2006 . 16607023 PMC1459665

[69] Gautschi M, Lilie H, Fünfschilling U, Mun A, Ross S, Lithgow T, et al. RAC, a stable ribosome-associated complex in yeast formed by the DnaK-DnaJ homologs Ssz1p and zuotin. Proc Natl Acad Sci U S A. 2001;98(7):3762–3767. doi: 10.1073/pnas.071057198 . 11274393 PMC31126

[70] Jaiswal H, Conz C, Otto H, Wölfle T, Fitzke E, Mayer M, et al. The chaperone network connected to human ribosome-associated complex. Mol Cell Biol. 2011;31(6):1160–1173. doi: 10.1128/MCB.00986-10 . 21245388 PMC3067906

[71] Kaiser C, Goldman D, Chodera J, Tinoco I, Bustamante C. The ribosome modulates nascent protein folding. Science. 2011;334(6063). doi: 10.1126/science.1209740 . 22194581 PMC4172366

[72] Samelson AJ, Jensen MK, Soto RA, Cate JH, Marqusee S. Quantitative determination of ribosome nascent chain stability. Proc Natl Acad Sci U S A. 2016;113(47):13402–7. Epub 20161107. doi: 10.1073/pnas.1610272113 ; PubMed Central PMCID: PMC5127326. 27821780 PMC5127326

[73] Deckert A, Waudby CA, Wlodarski T, Wentink AS, Wang X, Kirkpatrick JP, et al. Structural characterization of the interaction of α-synuclein nascent chains with the ribosomal surface and trigger factor. Proc Natl Acad Sci U S A. 2016;113(18):5012–7. Epub 20160418. doi: 10.1073/pnas.1519124113 ; PubMed Central PMCID: PMC4983817. 27092002 PMC4983817

[74] Klein DJ, Moore PB, Steitz TA. The contribution of metal ions to the structural stability of the large ribosomal subunit. RNA. 2004;10(9):1366–1379. doi: 10.1261/rna.7390804 15317974 PMC1370624

[75] Deckert A, Cassaignau AME, Wang X, Włodarski T, Chan SHS, Waudby CA, et al. Common sequence motifs of nascent chains engage the ribosome surface and trigger factor. Proc Natl Acad Sci U S A. 2021;118(52). doi: 10.1073/pnas.2103015118 ; PubMed Central PMCID: PMC8719866. 34930833 PMC8719866

[76] Lee EJ, Choi J, Groisman EA. Control of a Salmonella virulence operon by proline-charged tRNA(Pro). Proc Natl Acad Sci U S A. 2014;111(8):3140–3145. doi: 10.1073/pnas.1316209111 ; PubMed Central PMCID: PMC3939920. 24516160 PMC3939920

[77] Datsenko KA, Wanner BL. One-step inactivation of chromosomal genes in *Escherichia coli* K-12 using PCR products. Proc Natl Acad Sci U S A. 2000;97(12):6640–6645. doi: 10.1073/pnas.120163297 . 10829079 PMC18686

[78] Wahl A, Huptas C, Neuhaus K. Comparison of rRNA depletion methods for efficient bacterial mRNA sequencing. Sci Rep. 2022;12(1):5765. doi: 10.1038/s41598-022-09710-y 35388078 PMC8986838

[79] Bogdanov AA. Ribosomal RNA. In: Spirin AS, editor. Ribosomes. Boston, MA: Springer US; 1999. p. 75–95.

[80] Webb M. A continuous spectrophotometric assay for inorganic phosphate and for measuring phosphate release kinetics in biological systems. Proc Natl Acad Sci U S A. 1992;89(11). doi: 10.1073/pnas.89.11.4884 . 1534409 PMC49192

[81] Tomoyasu T, Mogk A, Langen H, Goloubinoff P, Bukau B. Genetic dissection of the roles of chaperones and proteases in protein folding and degradation in the *Escherichia coli* cytosol. Mol Microbiol. 2001;40(2):397–413. doi: 10.1046/j.1365-2958.2001.02383.x . 11309122

[82] Doyle S, Hoskins J, Wickner S. Collaboration between the ClpB AAA+ remodeling protein and the DnaK chaperone system. Proc Natl Acad Sci U S A. 2007;104(27). doi: 10.1073/pnas.0703980104 . 17545305 PMC2040865

[83] Genest O, Hoskins J, Camberg J, Doyle S, Wickner S. Heat shock protein 90 from *Escherichia coli* collaborates with the DnaK chaperone system in client protein remodeling. Proc Natl Acad Sci U S A. 2011;108(20):8206–8211. doi: 10.1073/pnas.1104703108 . 21525416 PMC3100916

