# Supplementary figures and images for: Chaperone Hsp70 helps Salmonella survive infection-relevant stress by reducing protein synthesis
Source: PLoS Biol. 2024 Apr 4;22(4):e3002560. doi: 10.1371/journal.pbio.3002560 (PMC10994381; doi:10.1371/journal.pbio.3002560)

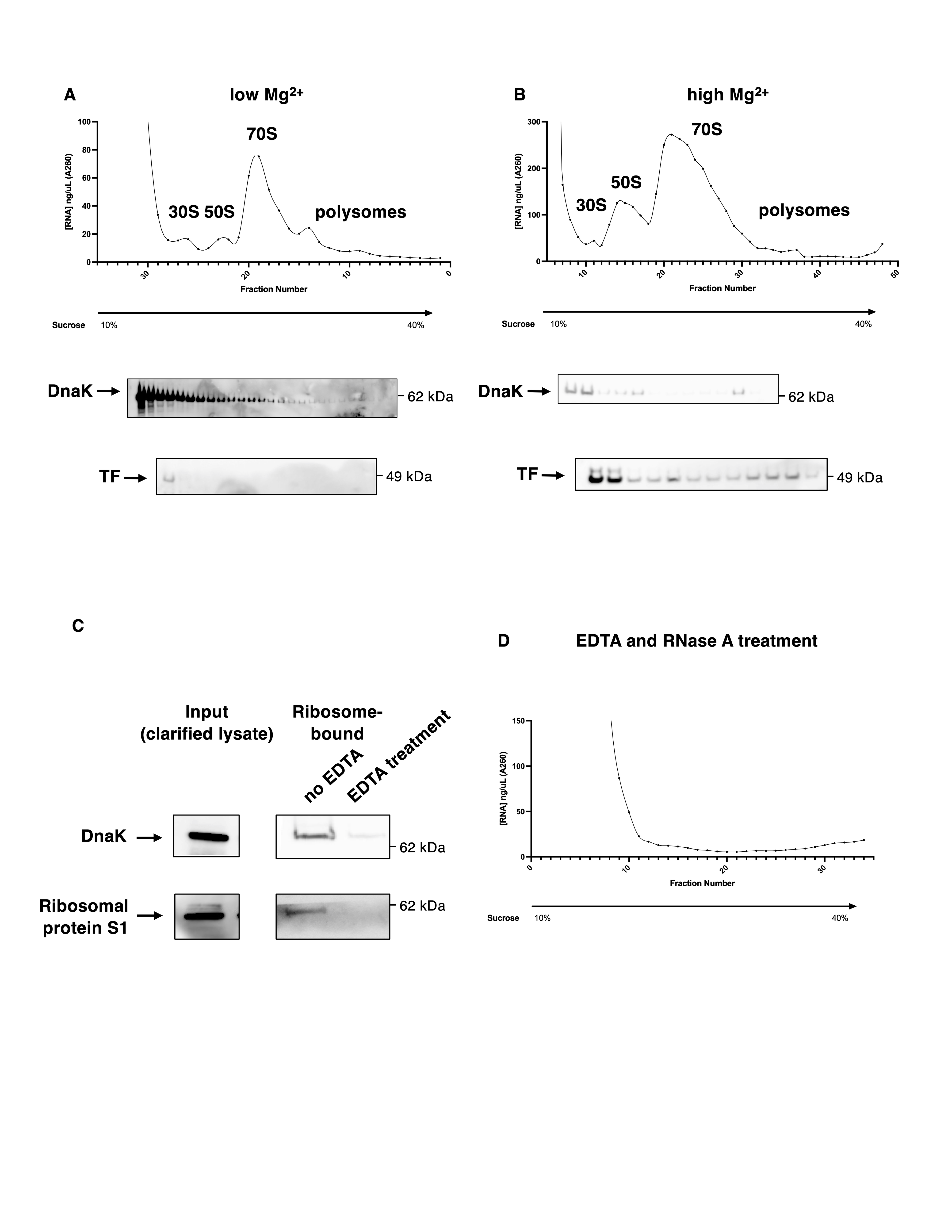

Supplement: S1 Fig — (A, B) Polysome profile analysis of wild-type (14028s) S. Typhimurium following 5 h of growth in low (10 μM) Mg2+ or 4.5 h of growth in high (10 mM) Mg2+ and western blot analysis of the corresponding fractions. Blot was developed using antibodies recognizing DnaK and TF. (C) Western blot analysis of clarified cell lysates (left) and ultracentrifuged fractions (right) of wild-type (14028s) S. Typhimurium following 5 h of growth in low (10 μM) Mg2+ using a sucrose cushion treated with EDTA. Blot was developed using antibodies recognizing DnaK or the ribosomal protein control S1. (D) Polysome profile analysis of wild-type S. Typhimurium lysate treated with EDTA and RNase A to fully dismantle ribosomes. Shown in (A and B) and (C and D) are the representatives of 3 and 2 independent biological replicates, respectively. (TIF) [file pbio.3002560.s001.tif]

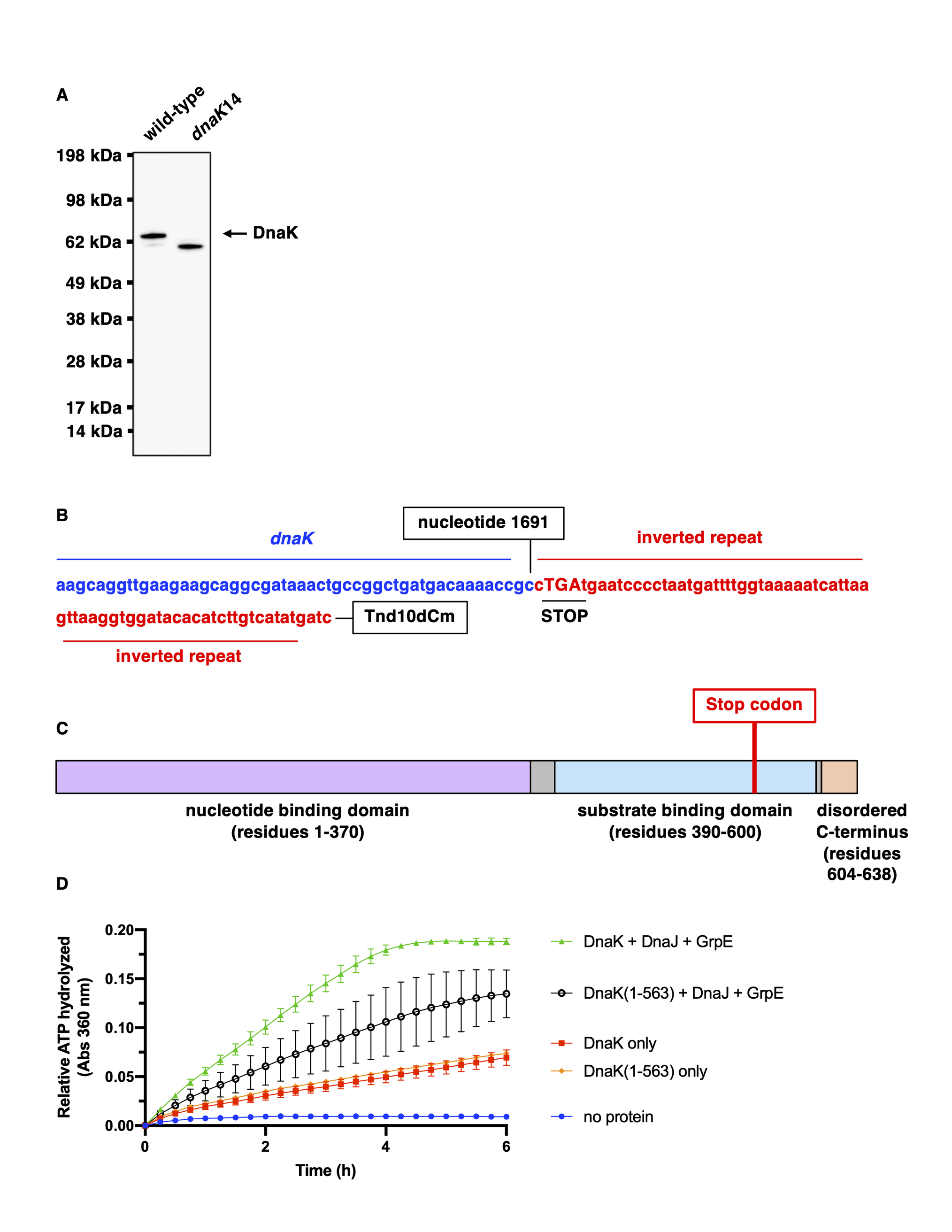

Supplement: S2 Fig — (A) Western blot of whole cell extract from wild-type (14028s) and dnaK14 (CC186) S. Typhimurium following 5 h of growth in low (10 μM) Mg2+. Blot was developed with polyclonal antibodies directed to DnaK. (B) Schematic of dnaK gene in mutant dnaK14. Transposon Tn10dCm inserted immediately after nucleotide 1691 in the dnaK coding region. In blue are nucleotides corresponding to the dnaK coding region near the site of Tn10dCm insertion. In red are nucleotides corresponding to transposon Tn10dCm, including the inverted repeat originating from transposon Tn10. In capital letters is the TGA stop codon in frame with the dnaK coding region. (C) Schematic of the domain architecture of wild-type DnaK protein. Transposon Tn10dCm in mutant dnaK14 provides an early stop codon in frame that results in the production of a truncated DnaK protein. (D) ATP hydrolysis in the presence or absence of purified full-length or truncated DnaK (2 μM) alone or in combination with cochaperone DnaJ (0.4 μM) and nucleotide exchange factor GrpE (0.2 μM). Shown in (A) is the representative of 4 independent biological replicates. Data in (D) represent mean ± SD of 3 independent assays performed in buffer containing 20 mM Mg2+. The numerical values underlying this figure can be found in S1 Data. (TIF) [file pbio.3002560.s002.tif]

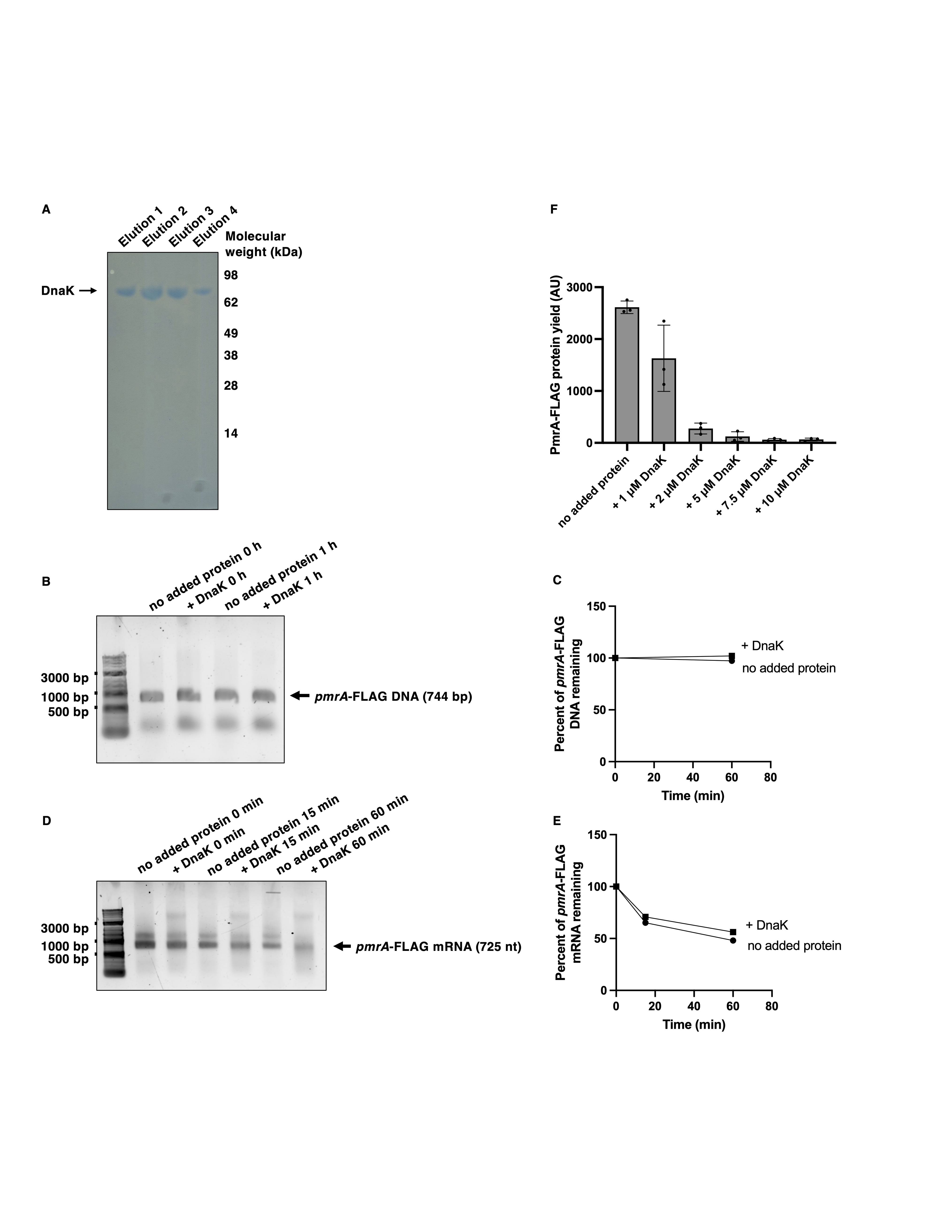

Supplement: S3 Fig — (A) Coomassie blue staining of DnaK protein following recombinant expression in E. coli BL21(DE3) and purification. (B, C) Stability of pmrA-FLAG DNA in the presence of purified DnaK (5 μM). (D, E) Stability of pmrA-FLAG mRNA in the presence of purified DnaK (5 μM). (F) Quantification of in vitro synthesized PmrA-FLAG protein in the presence of varying concentrations of purified DnaK. Data represent mean ± SD of 3 independent assays performed in buffer containing 9 mM Mg2+. The numerical values underlying this figure can be found in S1 Data. (TIF) [file pbio.3002560.s003.tif]

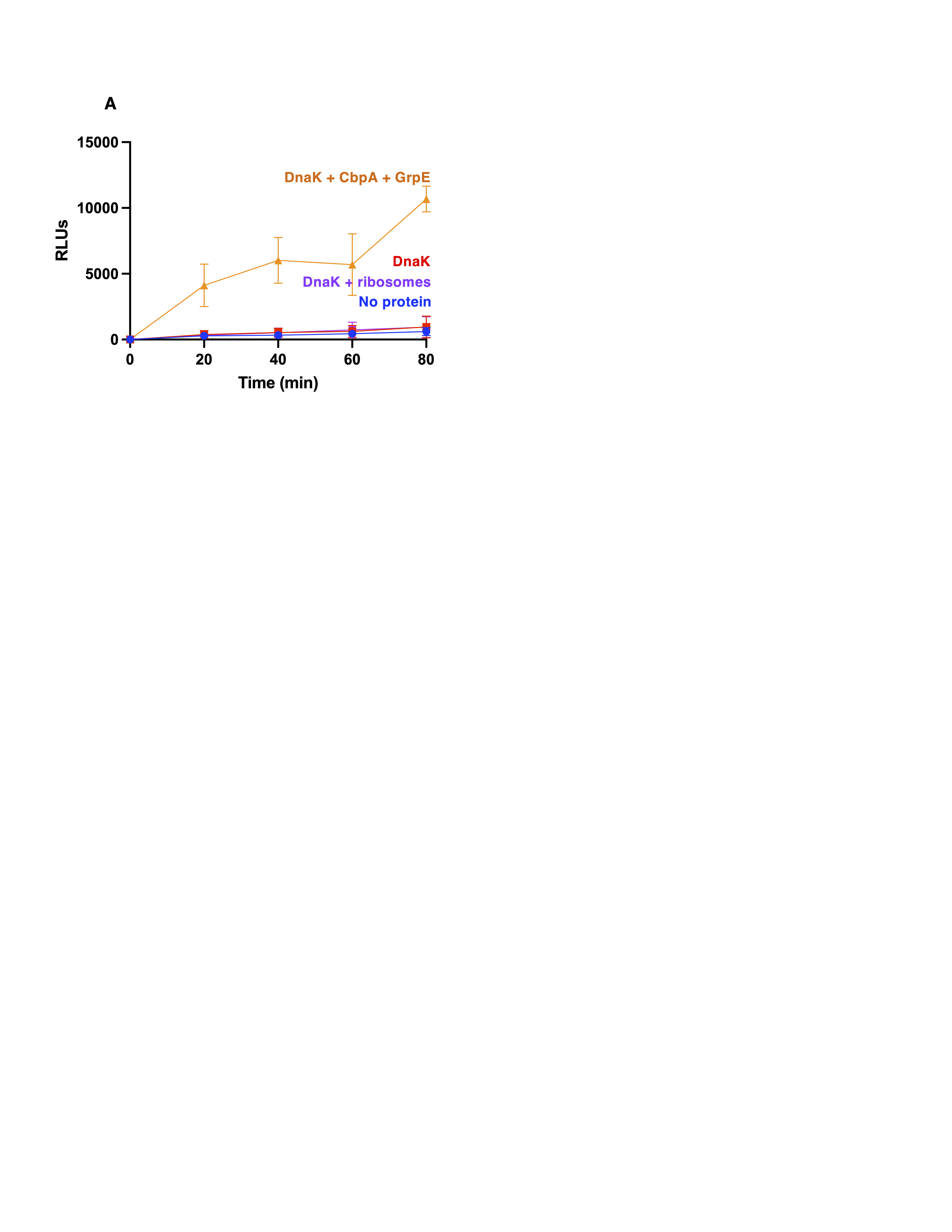

Supplement: S4 Fig — (A) Reactivation of heat-denatured luciferase in the presence or absence of purified DnaK (2 μM) alone or in combination with cochaperones (CbpA [0.4 μM] and GrpE [0.2 μM]) or 70S ribosomes (0.5 μM). Data represent mean ± SD of 3 independent assays. The numerical values underlying this figure can be found in S1 Data. (TIF) [file pbio.3002560.s004.tif]

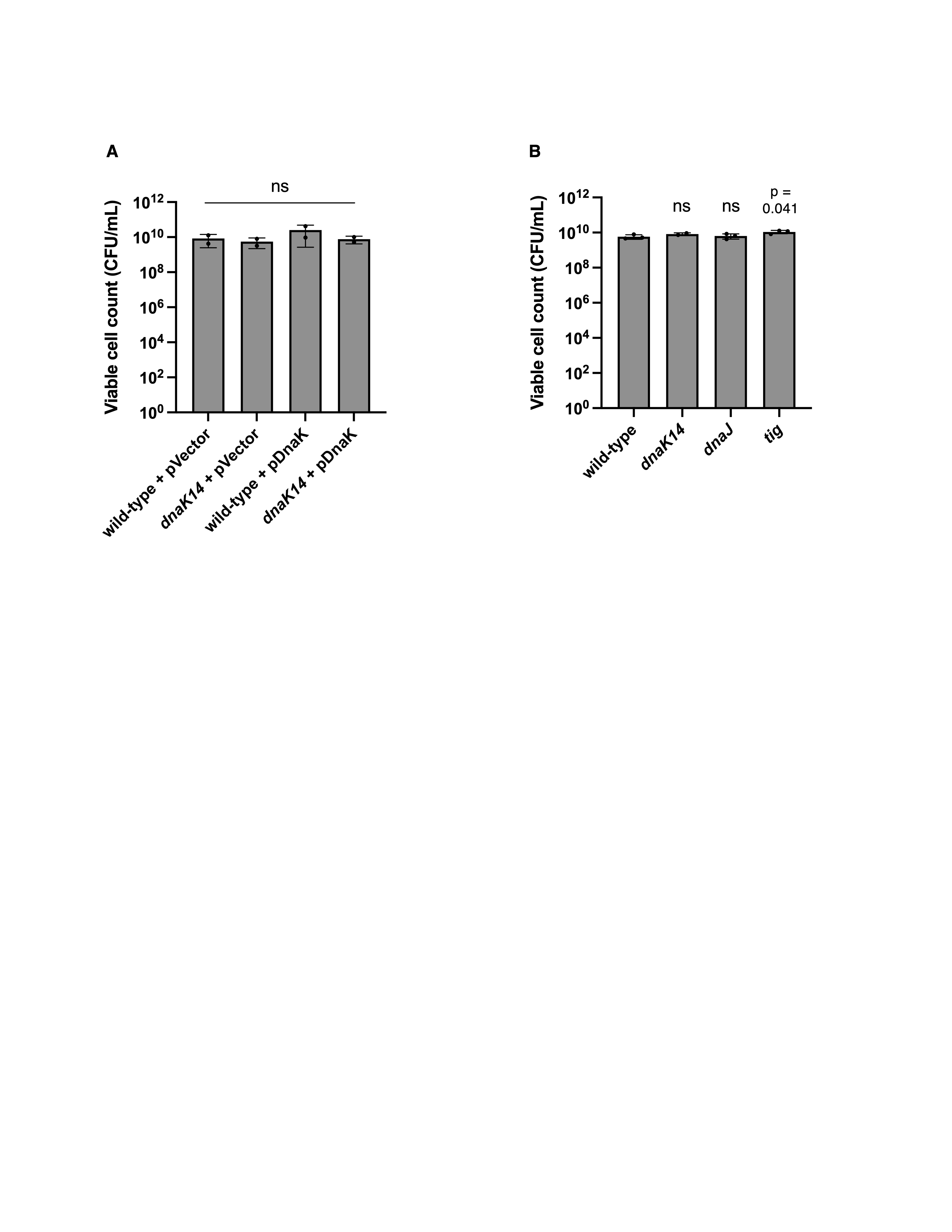

Supplement: S5 Fig — (A) Survival of wild-type (14028s) and dnaK14 (CC186) S. Typhimurium harboring the plasmid vector (pUHE-21-2-lacIq) or dnaK-expressing plasmid (pDnaK) following 24 h in high (10 mM) Mg2+. (B) Survival of wild-type (14028s), dnaK14 (CC186), dnaJ (EG16309), and tig (CC361) S. Typhimurium following 24 h in high (10 mM) Mg2+. Data represent mean ± SD of 2 independent biological replicates in (A) and 3 independent biological replicates in (B). Statistical analysis was performed using two-tailed Student’s t test comparing the indicated sample group to the wild-type sample group (ns = not significant). The numerical values underlying this figure can be found in S1 Data. (TIF) [file pbio.3002560.s005.tif]

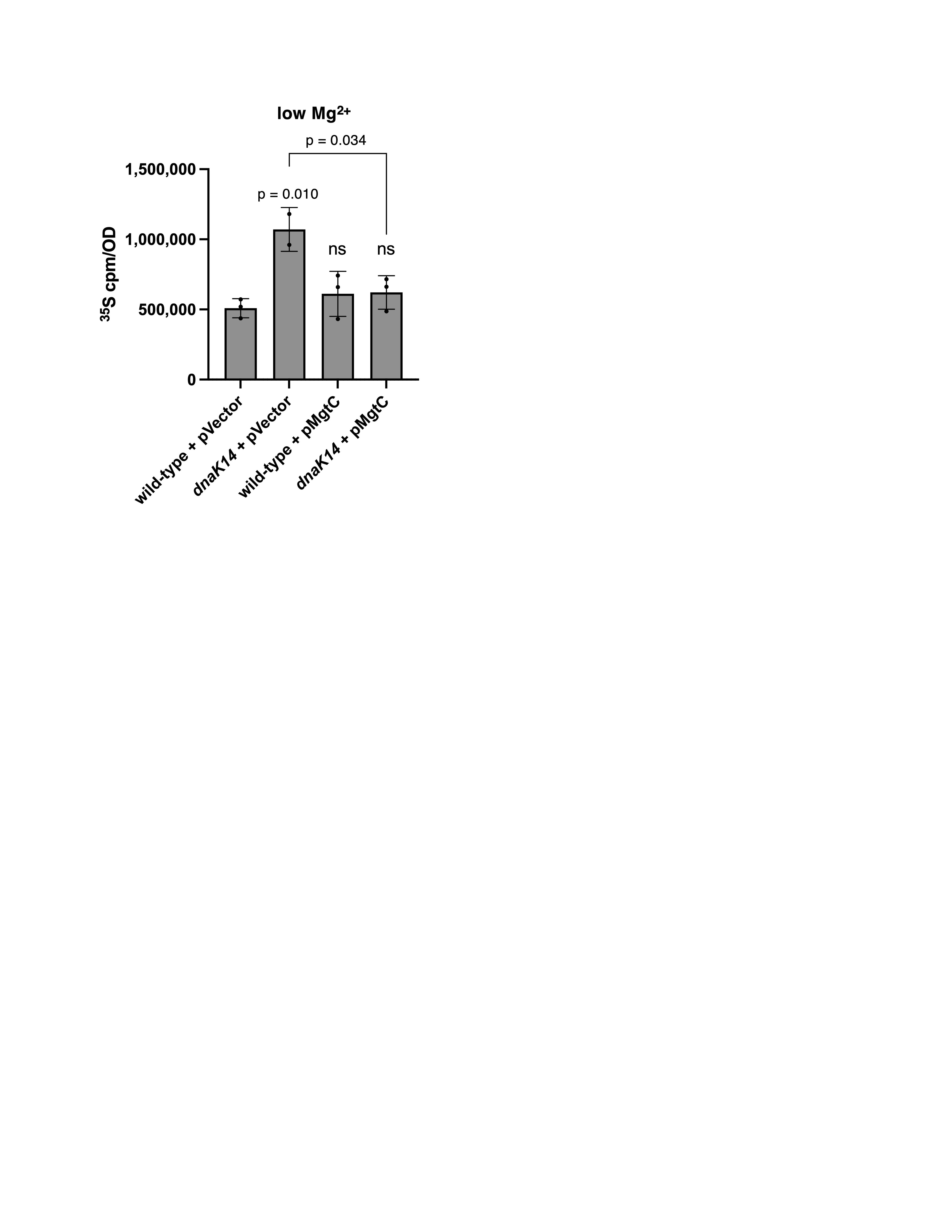

Supplement: S6 Fig — (A) 35S-methionine labeling of wild-type (14028s) and dnaK14 (CC186) S. Typhimurium harboring the plasmid vector (pUHE-21-2-lacIq) or mgtC-expressing plasmid (pMgtC) following 5 h of growth in low (10 μM) Mg2+. Data represent mean ± SD of 3 independent biological replicates. The numerical values underlying this figure can be found in S1 Data. (TIF) [file pbio.3002560.s006.tif]
