# Supplementary material for: Chaperone Hsp70 helps Salmonella survive infection-relevant stress by reducing protein synthesis
Source: PLoS Biol. 2024 Apr 4;22(4):e3002560. doi: 10.1371/journal.pbio.3002560 (PMC10994381; doi:10.1371/journal.pbio.3002560)
Supplement: S1 Table — (DOCX) [file pbio.3002560.s007.docx]

**Table S1. Strains and plasmids used in this study**

| **Strains** | **Relevant characteristics** | **Source** |
| --- | --- | --- |
| ***Salmonella enterica* serovar Typhimurium** |  |  |
| 14028s | wild-type | (1) |
| EG11507 | *dnaK*14::Tn10dCm (insertion at nucleotide position 1691) |  |
| CC186 | *dnaK*14::Tn10dCm (fresh transductions into 14028s) | This work |
| EG16309 | *dnaJ*::cm | This work |
| CC656 | *dnaJ, cbpA, djlA*::kan | This work |
| CC361 | *tig*::kan | This work |
| CC362 | *dnaK14*, *tig*::kan | This work |
| EL1 | *mgtC*::kan | (2) |
| CC241 | *dnaK14*, *mgtC*::kan | This work |
|  |  |  |
| ***Escherichia coli*** |  |  |
| DH5α | Host strain used for generation and propagation of plasmid constructs | (3) |
| BL21(DE3) | Host strain used for expression of recombinant proteins | (4) |
|  |  |  |
| **Plasmids** |  |  |
| pCP20 | rep_pSC101_^ts^ l cI857 FLP Amp^R^ Cm^R^ | (5) |
| pKD3 | rep_R6Kg_ Amp^R^ FRT Cm^R^ FRT | (5) |
| pKD4 | rep_R6Kg_ Amp^R^ FRT Km^R^ FRT | (5) |
| pKD46 | rep_pSC101_^ts^ Amp^R^ P_araBAD_-gbexo | (5) |
| pUHE-21-2-lacI^q^ | rep_pMB1_ lacI^q^ Amp^R^ vector control | (6) |
| pDnaK | pUHE-21-2-lacI^q^ -*dnaK* | This work |
| pDnaK(T199A) | pUHE-21-2-lacI^q^ -*dnaK*(a595g) | This work |
| pDnaK(1-563) | pUHE-21-2-lacI^q^ -*dnaK*(1-1689) | This work |
| pMgtC | pUHE-21-2-lacI^q^ -*mgtC* | (7) |
| pET-22b(+) | Vector for expression of recombinant proteins | Novagen |
| pDnaK-His6 | pET-22b(+)-*dnaK*-His6 | This work |
| pHtpG-His6 | pET-22b(+)-*htpG*-His6 | This work |
| pHis6-DnaJ | pUHE-21-2-lacI^q^ -His6-*dnaJ* | This work |
| pGrpE-His6 | pET-22b(+)-*grpE*-His6 | This work |
| pCbpA-His6 | pET-22b(+)-*cbpA*-His6 | This work |
| pDnaK(T199A)-His6 | pET-22b(+)-*dnaK*(a595g)-His6 | This work |
| pDnaK(1-563)-His6 | pET-22b(+)-*dnaK*(1-1689)-His6 | This work |
| pTF-His6 | pET-22b(+)-*tig*-His6 | This work |

References

1. Fields PI, Swanson RV, Haidaris CG, Heffron F. Mutants of *Salmonella typhimurium* that cannot survive within the macrophage are avirulent. Proc Natl Acad Sci USA. 1986;83(July 1986):5189-93.

2. Lee EJ, Pontes MH, Groisman EA. A bacterial virulence protein promotes pathogenicity by inhibiting the bacterium's own F1Fo ATP synthase. Cell. 2013;154(1):146-56.

3. Hanahan D, Glover D. DNA cloning: a practical approach. DNA cloning: a practical approach. 1985;1:109-35.

4. Studier FW, Moffatt BA. Use of bacteriophage T7 RNA polymerase to direct selective high-level expression of cloned genes. J Mol Biol. 1986;189(1):113-30.

5. Datsenko KA, Wanner BL. One-step inactivation of chromosomal genes in *Escherichia coli* K-12 using PCR products. Proc Natl Acad Sci USA. 2000;97(12):6640-5.

6. Soncini FC, Vescovi EG, Groisman EA. Transcriptional autoregulation of the *Salmonella* Typhimurium *phoPQ* operon. J Bacteriol. 1995;177(15):4364-71.

7. Chamnongpol S, Groisman EA. Mg2+ homeostasis and avoidance of metal toxicity. Mol Microbiol. 2002;44(2):561-71.
