# Supplementary material for: Chaperone Hsp70 helps Salmonella survive infection-relevant stress by reducing protein synthesis
Source: PLoS Biol. 2024 Apr 4;22(4):e3002560. doi: 10.1371/journal.pbio.3002560 (PMC10994381; doi:10.1371/journal.pbio.3002560)
Supplement: S2 Table — (DOCX) [file pbio.3002560.s008.docx]

Table S2. Oligonucleotides used in this study

| **Oligo name** | **Sequence (5’ to 3’)** | **Purpose** | **Source** |
| --- | --- | --- | --- |
| 18672 | GGAGAAATTAACTATGAGAGGATCC atgggtaaaattattggtatcgacctg | Cloning of *dnaK* into pUHE-21-2-lacI^q^ | This work |
| 18611 | GAGTCCAAGCTCAGCTAATTAAGCTTttattttttatcttttacttcttcaaactcagcg | Cloning of *dnaK* into pUHE-21-2-lacI^q^ | This work |
| 5776 | agaggtttcctctccgcccgtgtatgcatgttaagggcagataaaaagaggtgtaggctggagctgcttc | *dnaJ* inactivation | This work |
| 5779 | aaaaagcccggatatacacccgggctgaagaaaaatacaacgggaaaagacatatgaatatcctcctta | *dnaJ* inactivation | This work |
| 18714 | GAAACCTTTTGGGGTCCCTTCTGTATGTATTGATTTAGCGAGATGATGCT GTGTAGGCTGGAGCTGCTTC | *cbpA* inactivation | This work |
| 18715 | GGTGTGCAAACAAAATTCGGTGATGGTAAAGGTGACAGTGATGTTAGCCA CATATGAATATCCTCCTTAGT | *cbpA* inactivation | This work |
| 19353 | TTATTTTGTGGGAGATAGCCTCACCGATAGCGTAACGTTTTGGGGAGTCT GTGTAGGCTGGAGCTGCTTC | *djlA* inactivation | This work |
| 19354 | CATTCATTAACGTAGGCCGGATAAGGCGCTAGCCGCATCCGGCAATTCAA CATATGAATATCCTCCTTAGT | *djlA* inactivation | This work |
| con dnaJ-F | GTAATTACTGGCACGGGCGAAG | *dnaJ* inactivation verification | This work |
| con dnaJ-R | CAACAAATGAGAGGATACGATGCG | *dnaJ* inactivation verification | This work |
| con cbpA-F | CAGGTTTACCCTACACTTAGTGGAG | *cbpA* inactivation verification | This work |
| con cbpA-R | CTCCTCTTCCGTCACGCC | *cbpA* inactivation verification | This work |
| con djlA-F | GCCTTCATTTGCAGAAAAGCACC | *djlA* inactivation verification | This work |
| con djlA-R | CCCGGCCTACATTCATTAACGTAG | *djlA* inactivation verification | This work |
| 18625 | gtttaactttaagaaggagatatacatatgatgggtaaaattattggtatcgacctg | Cloning of *dnaK*-His6 into pET-22b(+) | This work |
| 18626 | cagtggtggtggtggtggtgctcgagttttttatcttttacttcttcaaactcagcg | Cloning of *dnaK*-His6 into pET-22b(+) | This work |
| 18762 | aactttaagaaggagatatacatatgATGGCATTGTTGAGGTCTATCCAC | Cloning of *htpG*-His6 into pET-22b(+) | This work |
| 18763 | cagtggtggtggtggtggtgctcgagGGACACCAGCAACTGGTTC | Cloning of *htpG*-His6 into pET-22b(+) | This work |
| 18881 | gaggagaaattaactatgagaggatccatgcaccaccaccaccaccacgcgaaaagagattactacgagattttag | Cloning of His6-*dnaJ* into pUHE-21-2-lacI^q^ | This work |
| 18659 | GAGTCCAAGCTCAGCTAATTAAGCTTttagcgagtcaaatcgtcaaag | Cloning of His6-*dnaJ* into pUHE-21-2-lacI^q^ | This work |
| 18649 | gtttaactttaagaaggagatatacatatgATGAGTAGTAAAGAACAGAAAACGCCTG | Cloning of *grpE*-His6 into pET-22b(+) | This work |
| 18650 | cagtggtggtggtggtggtgctcgagCTTCGCCTTCGCTACAGTCAC | Cloning of *grpE*-His6 into pET-22b(+) | This work |
| 18716 | aactttaagaaggagatatacatatgATGGAACTTAAGGATTATTACGCCATTATG | Cloning of *cbpA*-His6 into pET-22b(+) | This work |
| 18717 | cagtggtggtggtggtggtgctcgagTGCTTTCCCCCATTGCTG | Cloning of *cbpA*-His6 into pET-22b(+) | This work |
| 18554 | GCGAATTAATACGACTCACTATAGGGCTTAAGTATAAGGAGGAAAAAAT atgaaagaatataagatcttattagtagacgatcatgaaatcatcattaacggc | Generation of *ssrB*-FLAG DNA template for *in vitro* protein synthesis | This work |
| 18555 | AAACCCCTCCGTTTAGAGAGGGGTTATGCTAG ttaCTTGTCGTCATCGTCTTTGTAGTCatactctattaacctcattcttcgggcacagttaagtaac | Generation of *ssrB*-FLAG DNA template for *in vitro* protein synthesis | This work |
| 18558 | GCGAATTAATACGACTCACTATAGGGCTTAAGTATAAGGAGGAAAAAAT atgaagatactgattgttgaagacgacacgctattattacagggg | Generation of *pmrA*-FLAG DNA template for *in vitro* protein synthesis | This work |
| 18559 | AAACCCCTCCGTTTAGAGAGGGGTTATGCTAG ttaCTTGTCGTCATCGTCTTTGTAGTCgctttcctcagtggcaaccagcatgtagc | Generation of *pmrA*-FLAG DNA template for *in vitro* protein synthesis | This work |
| 18710 | GTGCTTGCGGAGTAGAGTTGACCGAGCACTGTGATTTTTTGAGGTAACAAG GTGTAGGCTGGAGCTGCTTC | *tig* inactivation | This work |
| 18711 | CTGAAAGGTGACGGGTTTTTGTGCAAATTTCGTGCTTTTAACGCGAAAAA CATATGAATATCCTCCTTAGT | *tig* inactivation | This work |
| con tig-F | CAAAATACGTGACAAAAAAAACCCGTC | *tig* inactivation verification | This work |
| con tig-R | CAAAATACGTGACAAAAAAAACCCGTC | *tig* inactivation verification | This work |
| 18725 | aactttaagaaggagatatacatatgATGCAAGTTTCAGTTGAAACCACTC | Cloning of *tig*-His6 into pET-22b(+) | This work |
| 18726 | cagtggtggtggtggtggtgctcgagCGCCTGCTGGTTCATCAG | Cloning of *tig*-His6 into pET-22b(+) | This work |
| 18764 | cgacctcggtggtggtgctttcgatatctctatta | Site-directed mutagenesis for pET-22b(+)-DnaK(T199A)-His6 | This work |
| 18765 | taatagagatatcgaaagcaccaccaccgaggtcg | Site-directed mutagenesis for pET-22b(+)-DnaK(T199A)-His6 | This work |
| 18734 | tttacgacctcggtggtggtGCTttcgatatctctattatcg | Site-directed mutagenesis for pUHE-21-2-lacI^q^-DnaK(T199A) | This work |
| 18735 | cgataatagagatatcgaaagcaccaccaccgaggtcgtaaa | Site-directed mutagenesis for pUHE-21-2-lacI^q^-DnaK(T199A) | This work |
| 18883 | caagctcagctaattaagcttttaggttttgtcatcagccggc | Cloning of DnaK(1-563) into pUHE-21-2-lacI^q^ | This work |
| 18891 | cagtggtggtggtggtggtgctcgagggttttgtcatcagccggc | Cloning of DnaK(1-563)-His6 into pET-22b(+) | This work |
