# Supplementary material for: Chaperone Hsp70 helps Salmonella survive infection-relevant stress by reducing protein synthesis
Source: PLoS Biol. 2024 Apr 4;22(4):e3002560. doi: 10.1371/journal.pbio.3002560 (PMC10994381; doi:10.1371/journal.pbio.3002560)

Strain construction verification

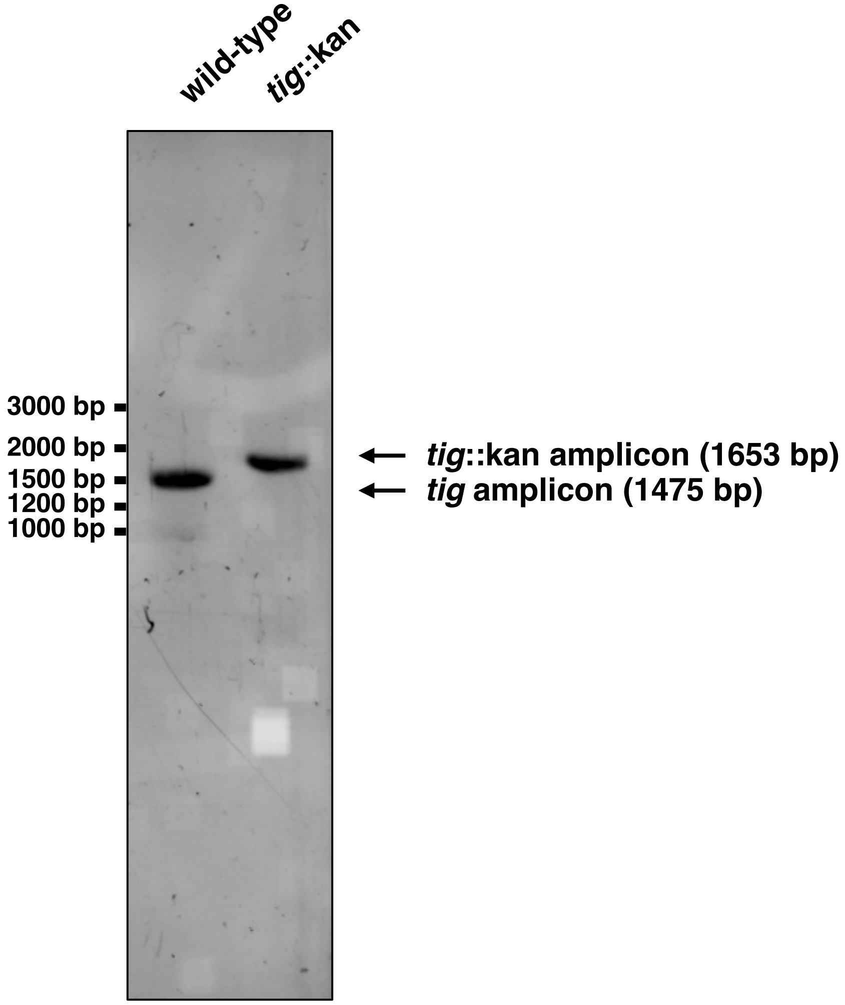

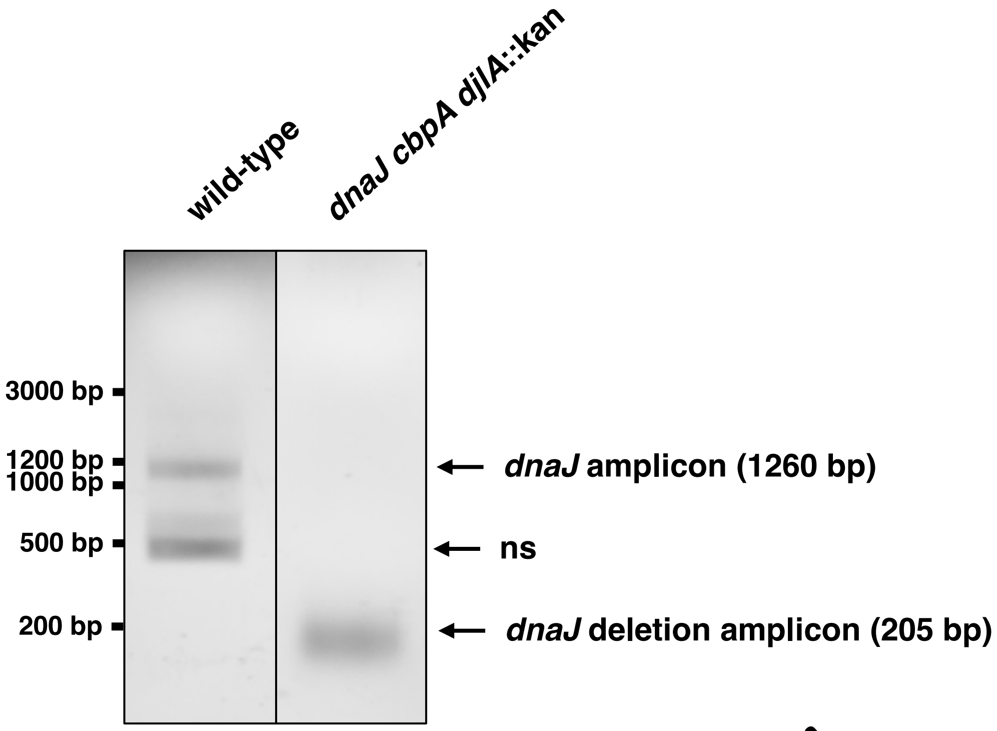

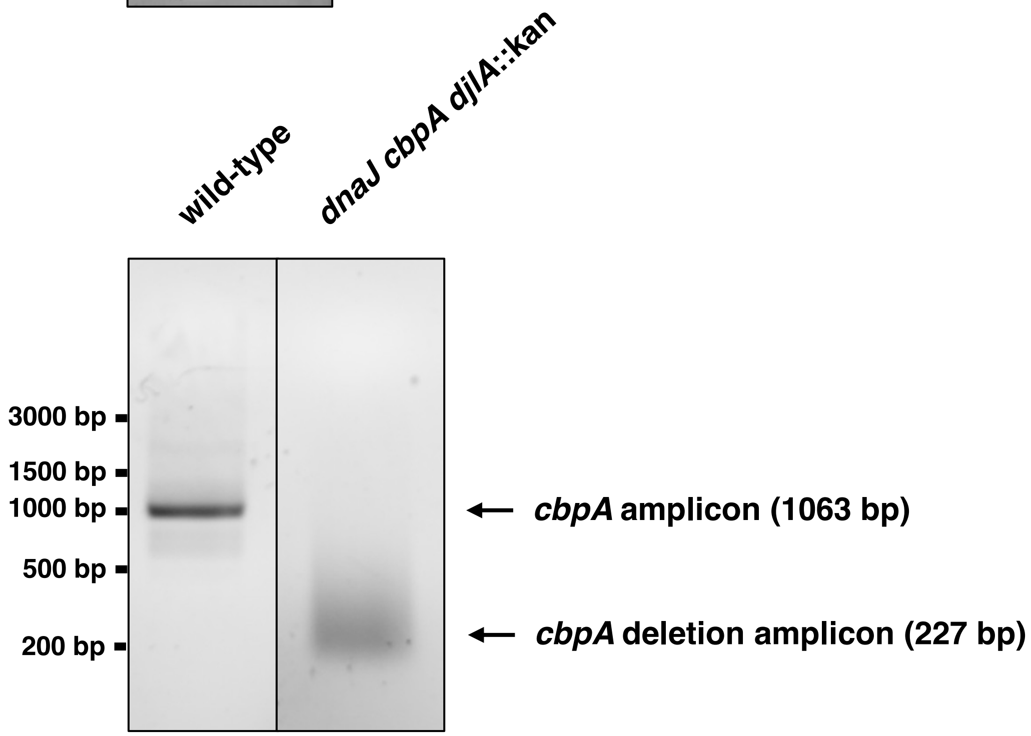

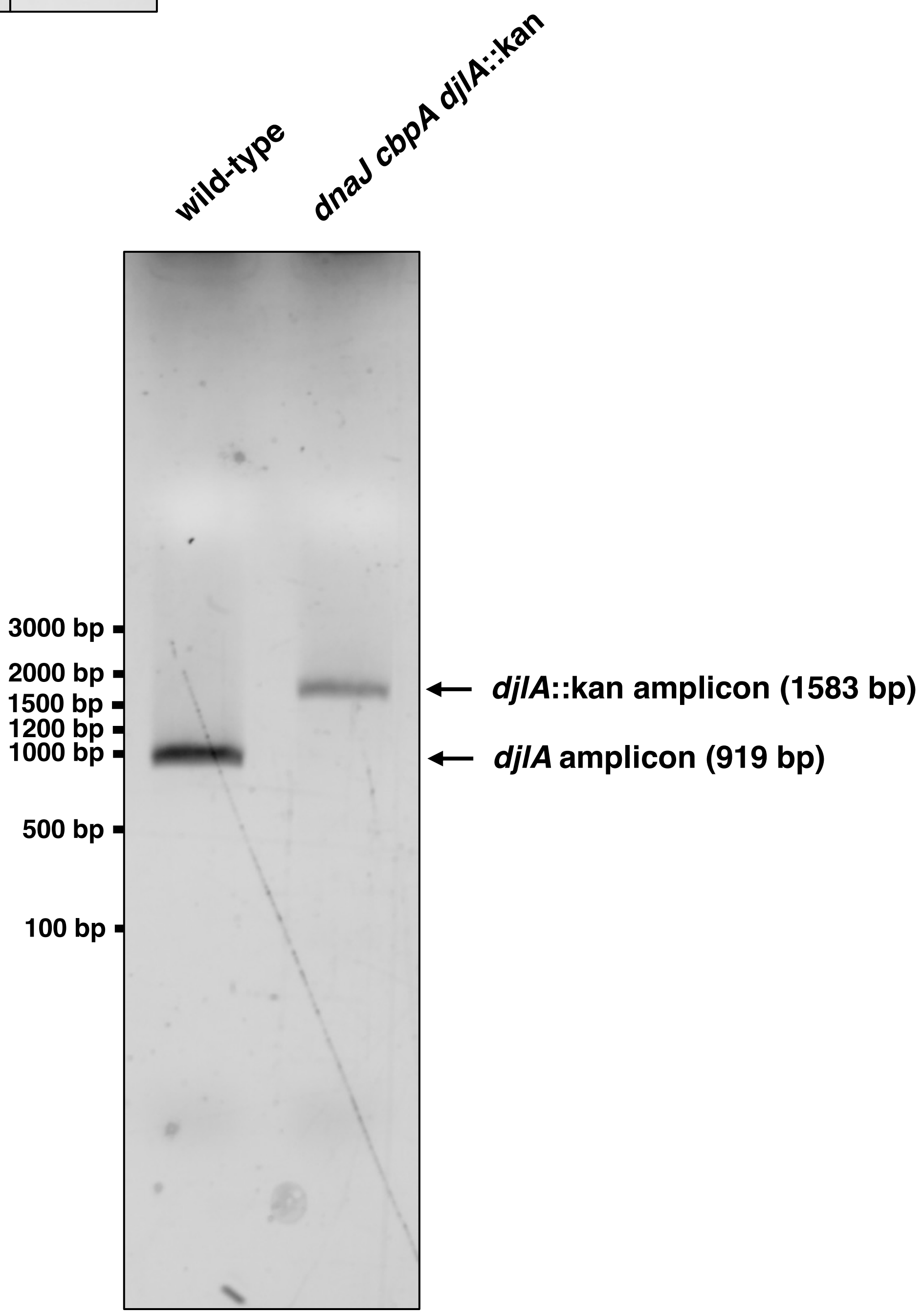

Corresponding to Figure 1B

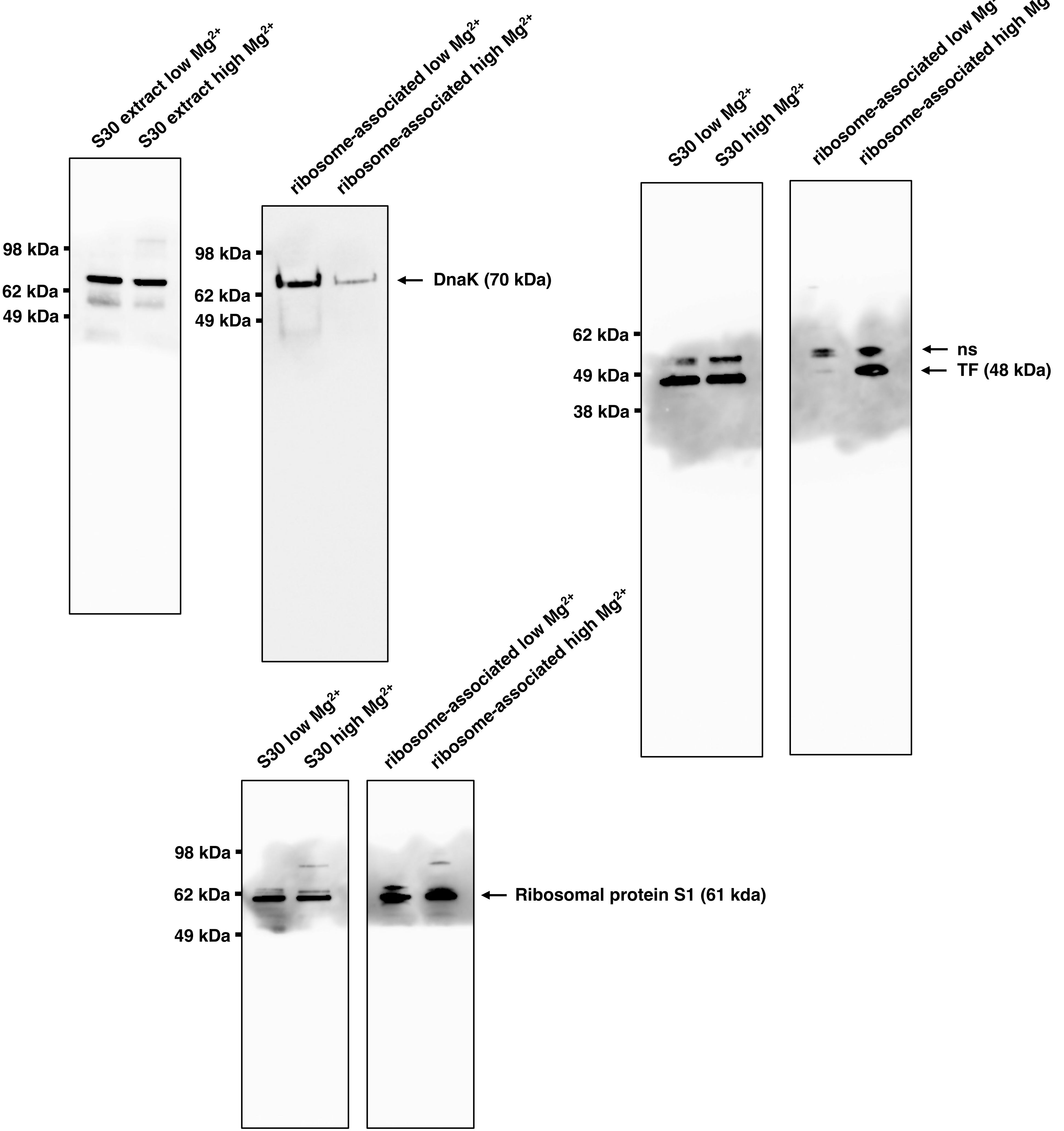

Corresponding to Figure 1B

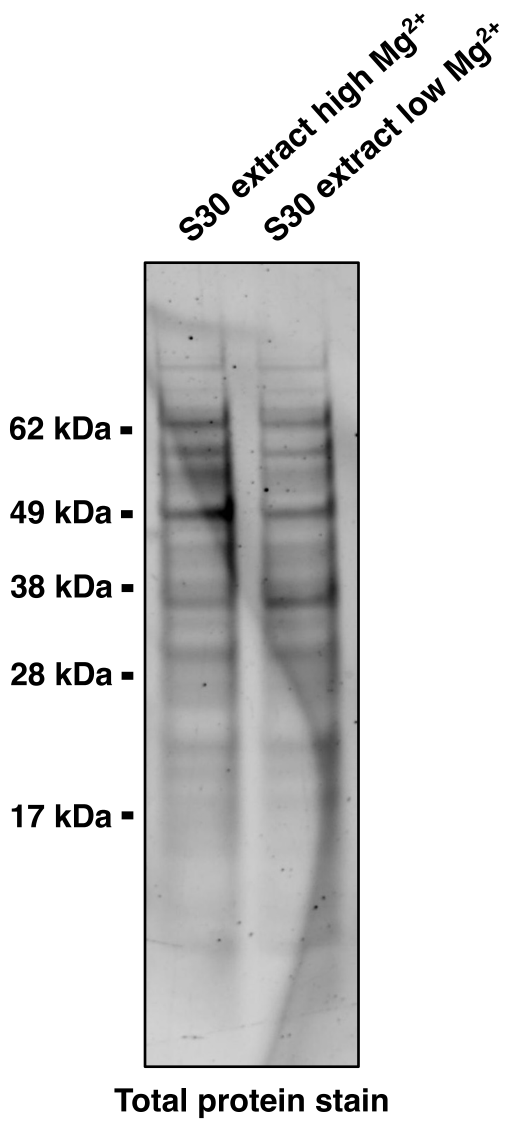

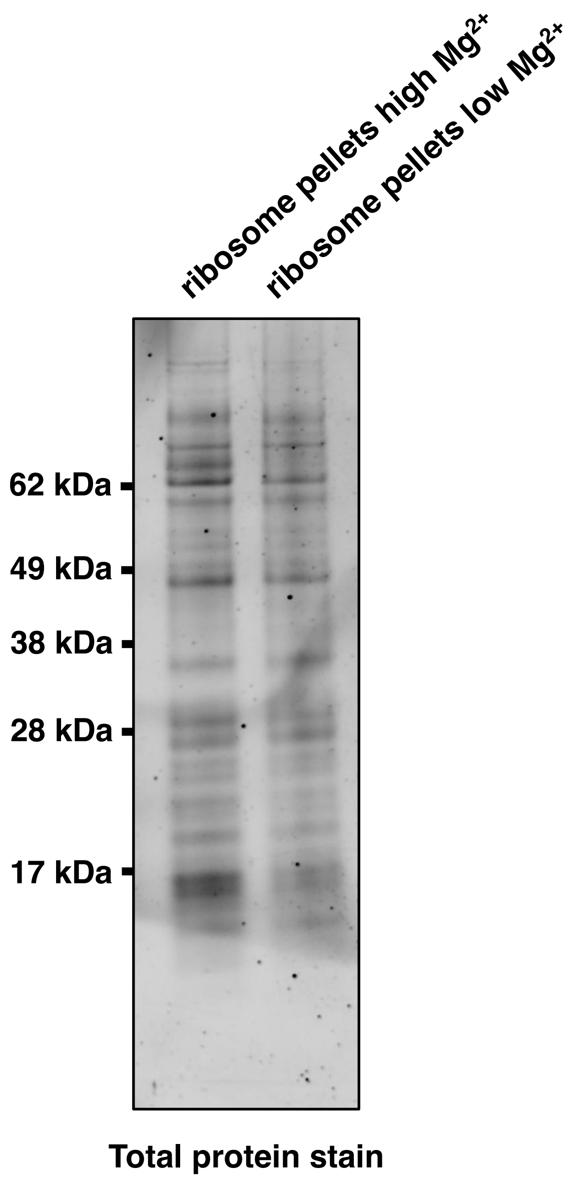

Corresponding to Figure 1C

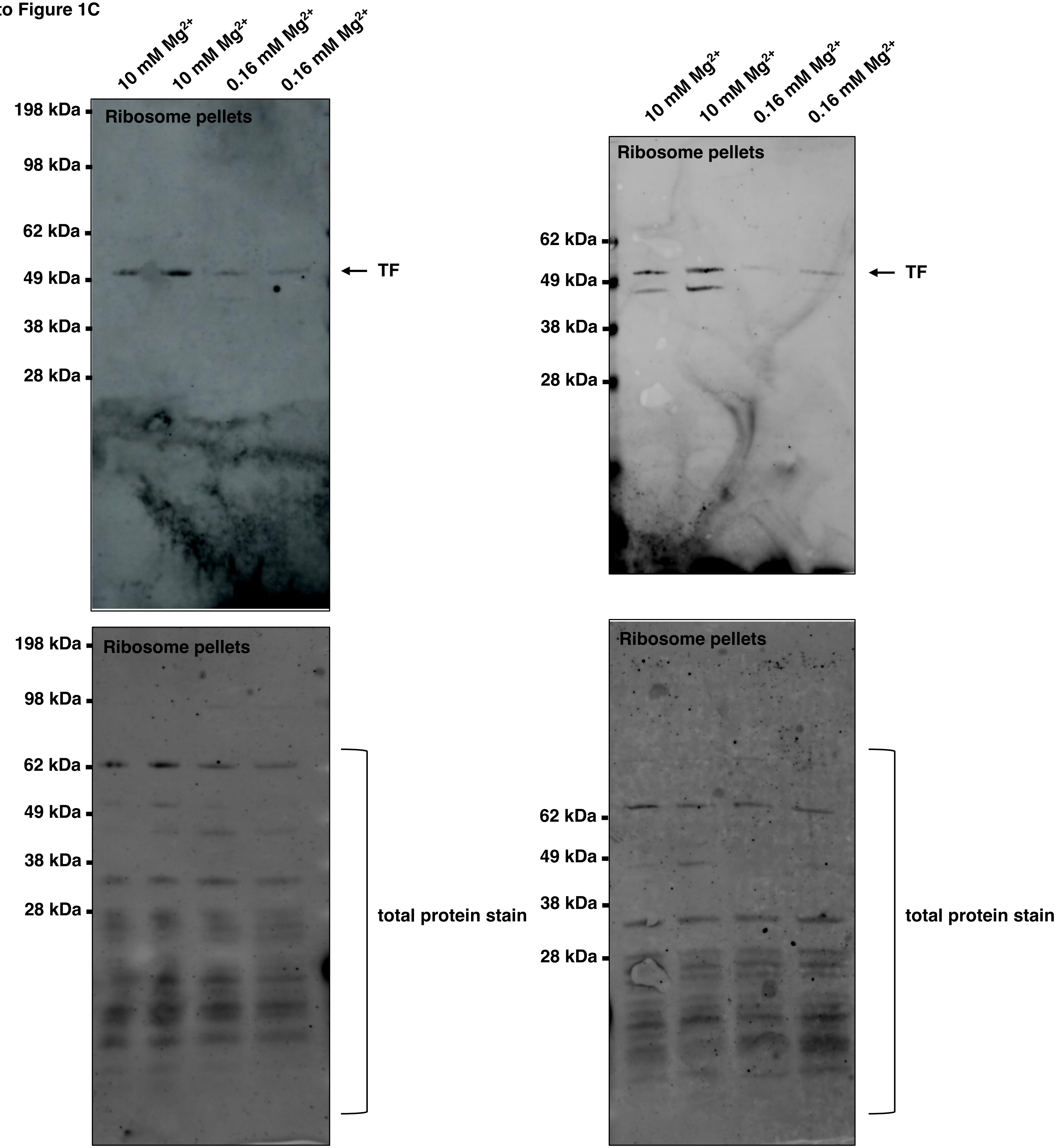

Corresponding to Figure 1D

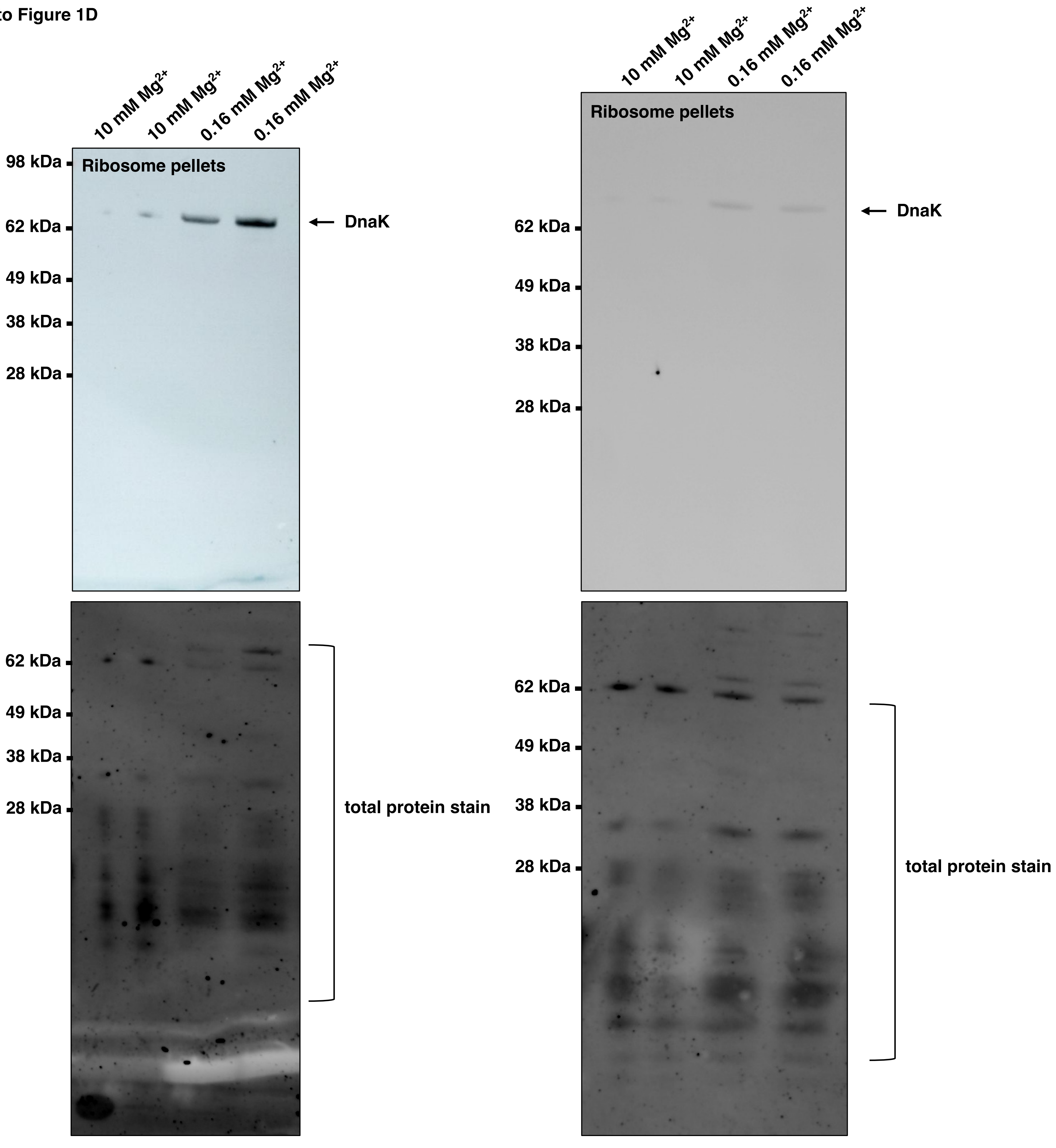

Corresponding to Figure S1C

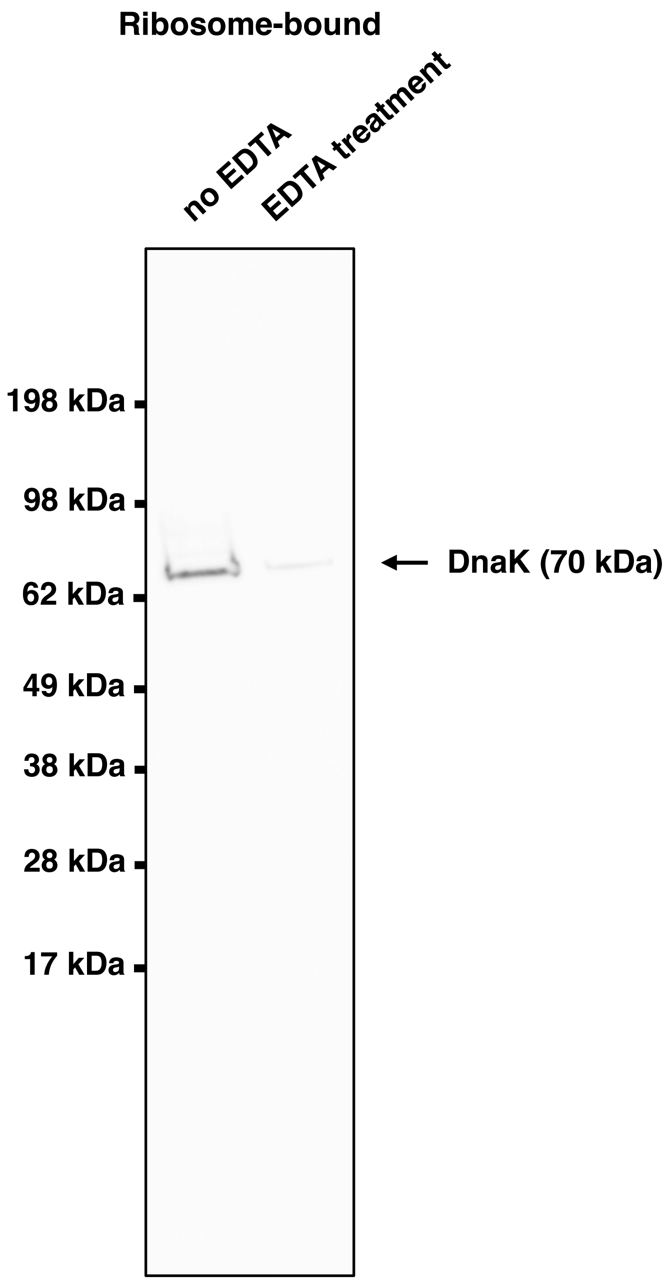

Corresponding to Figures S1A-B

low Mg^{2+} polysome profiling fractions

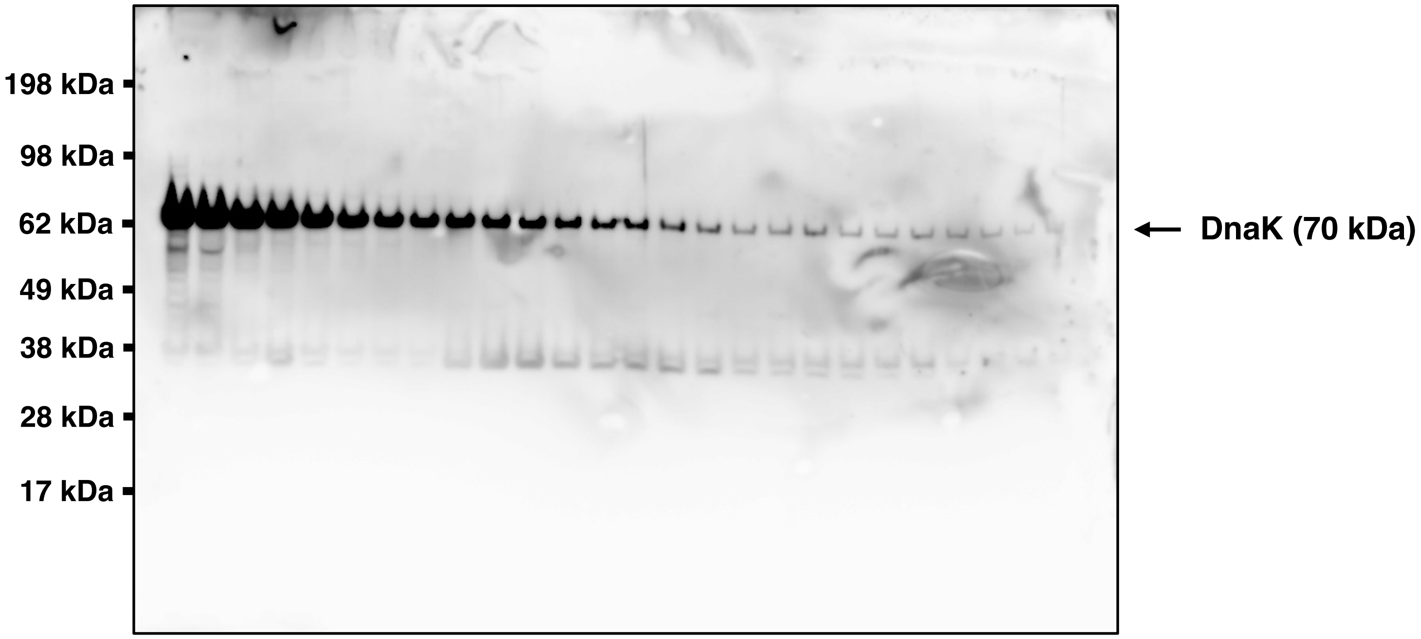

high Mg^{2+} polysome profiling fractions

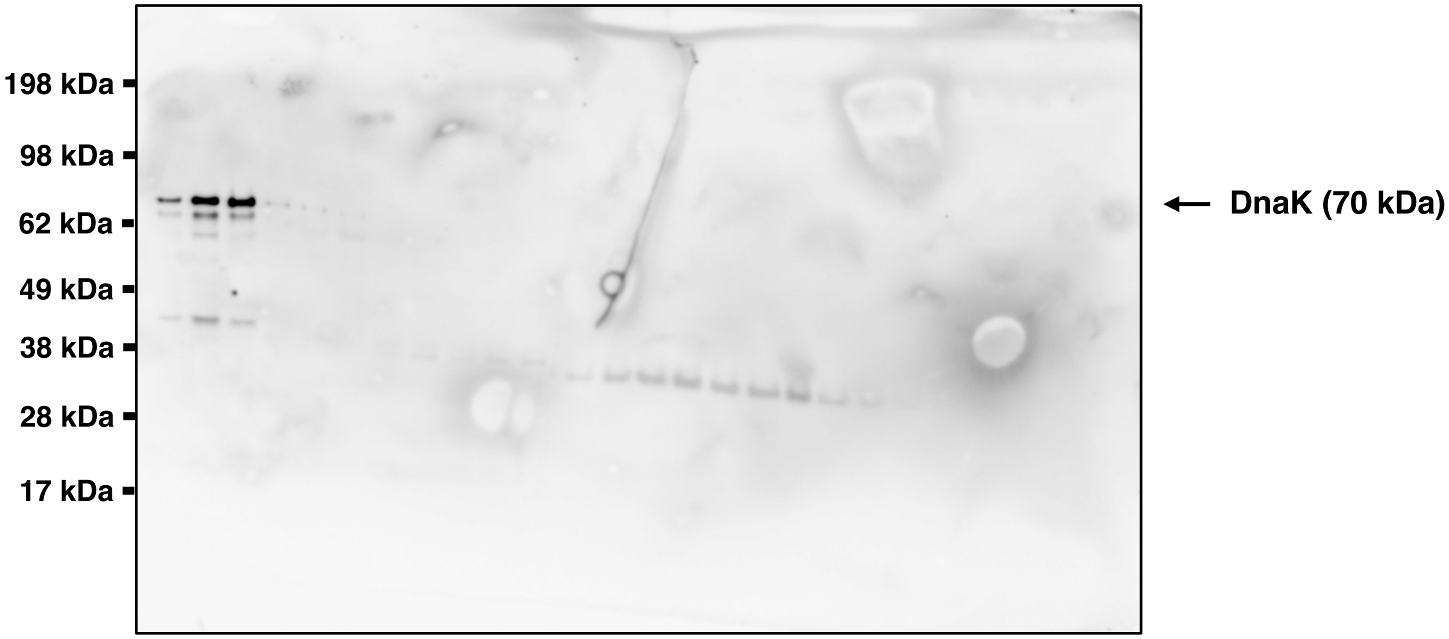

Corresponding to Figures S1A-B

low Mg^{2+} polysome profiling fractions

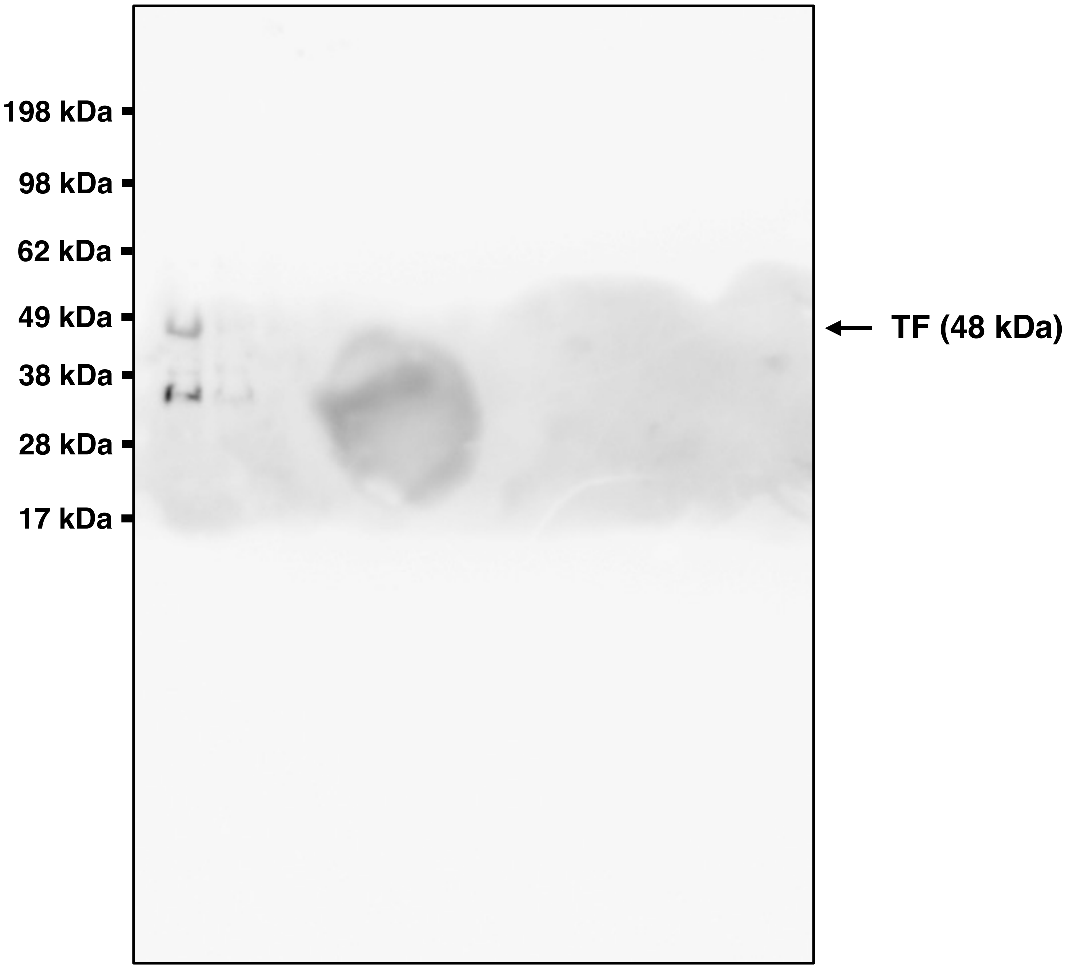

high Mg^{2+} polysome profiling fractions

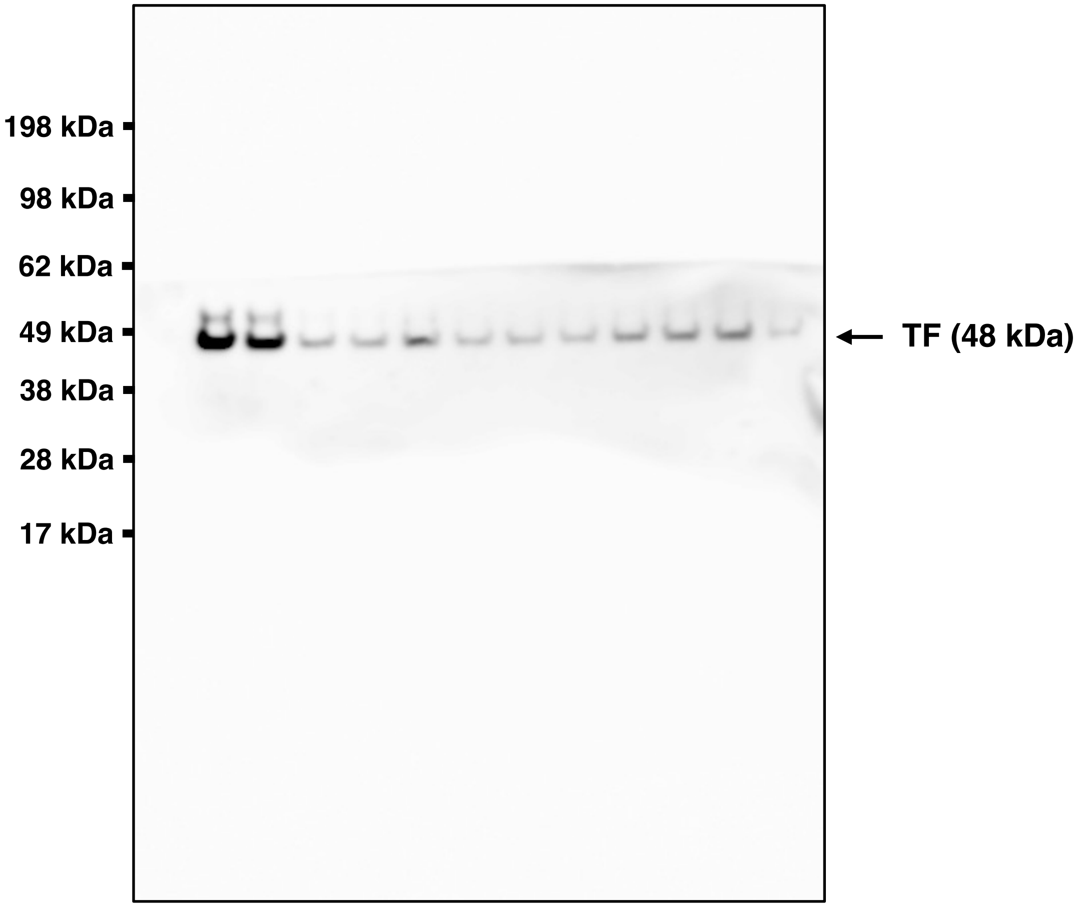

Corresponding to Figures 2A-B, 2I

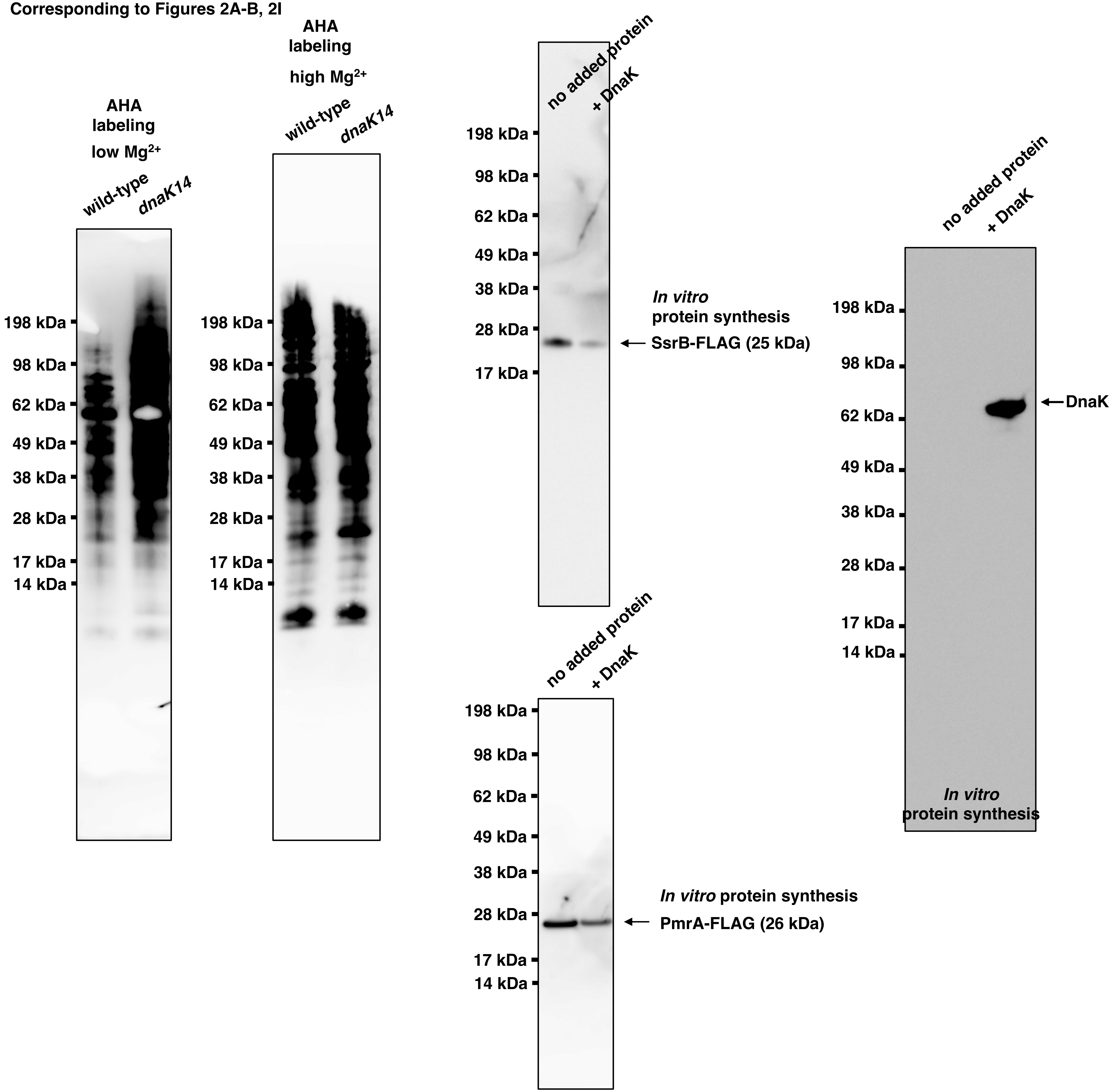

Corresponding to Figures 2J-K

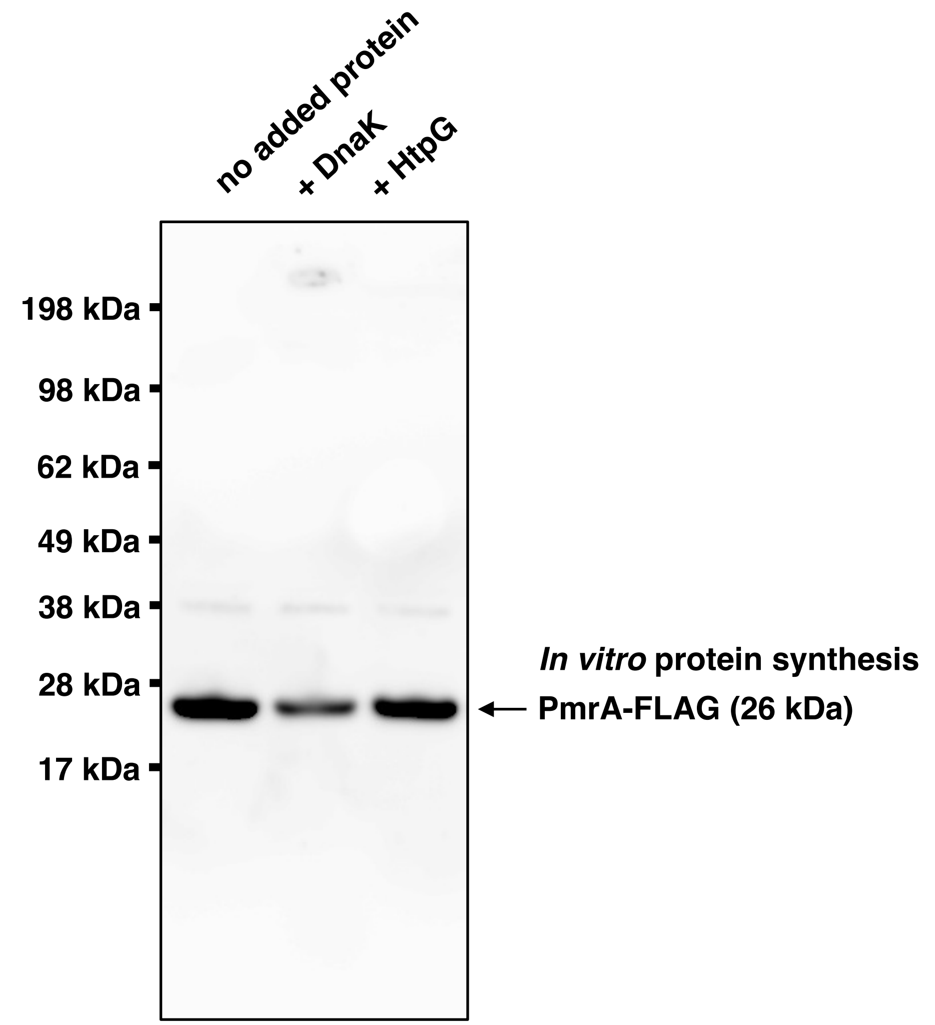

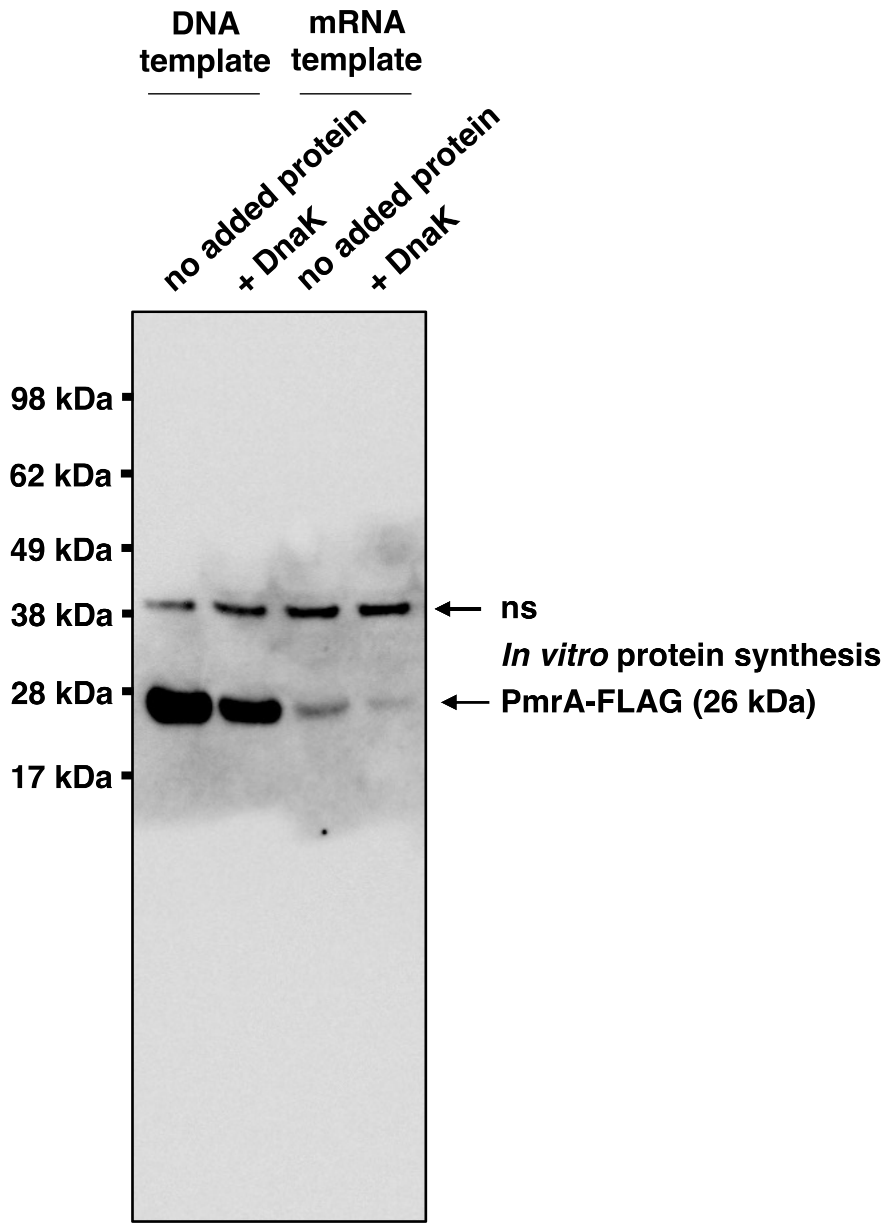

Quantifications corresponding to Figures 2I-K

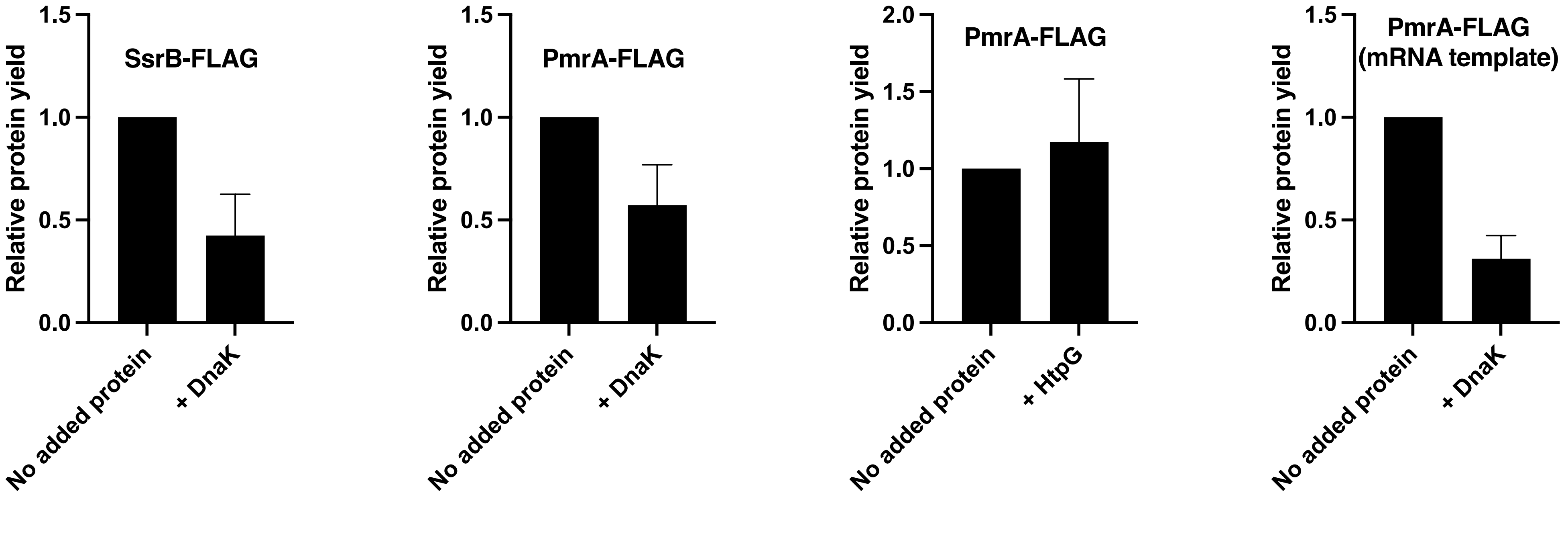

Corresponding to Figure 2G

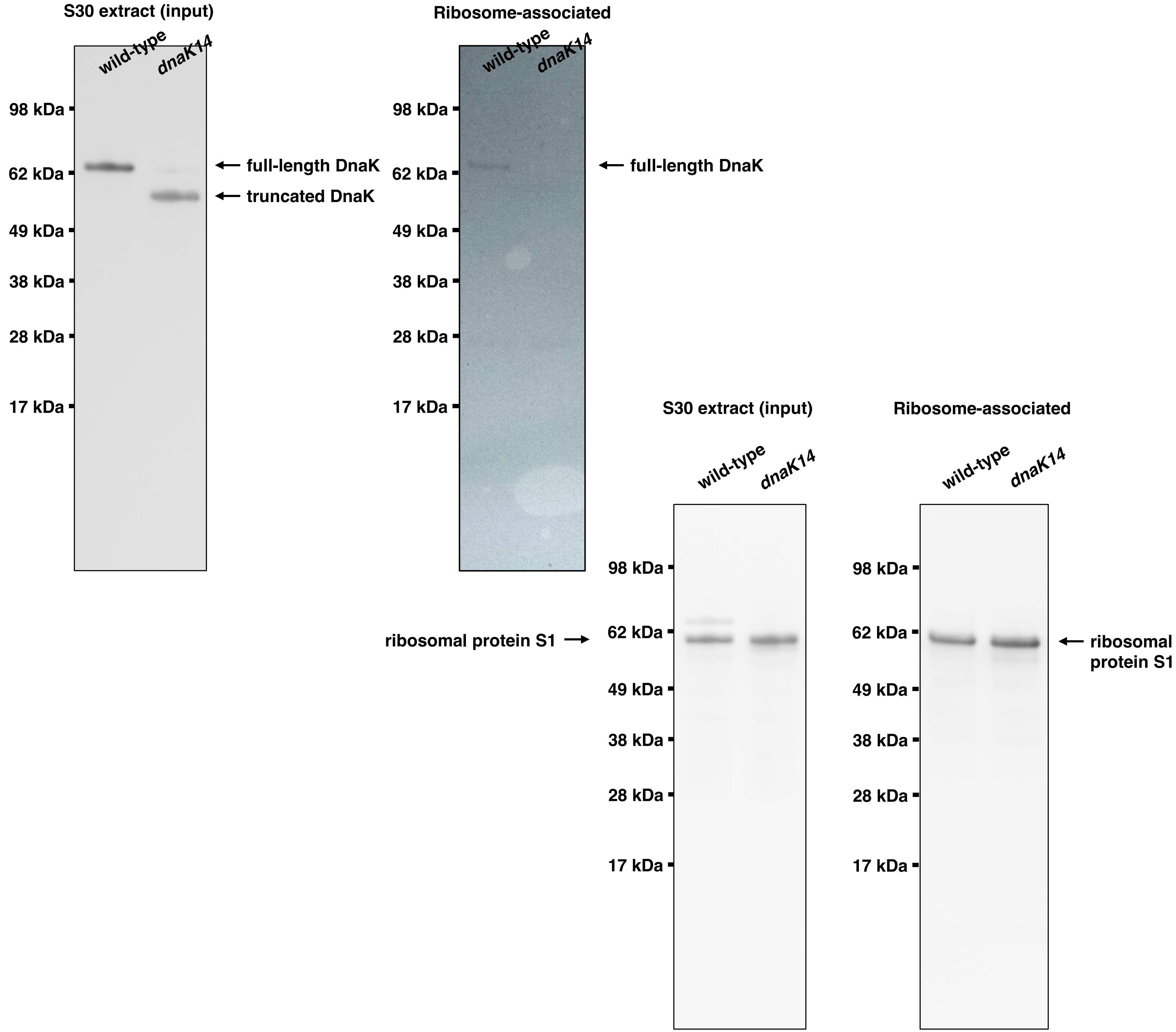

Corresponding to Figure 2G

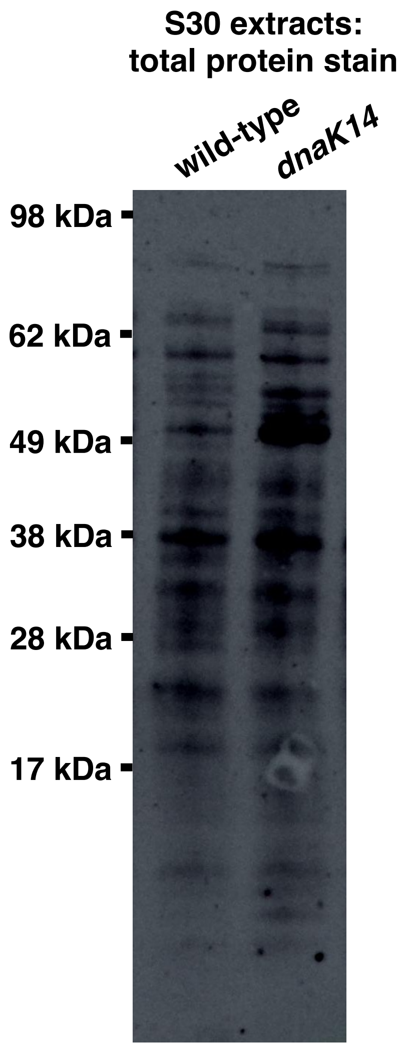

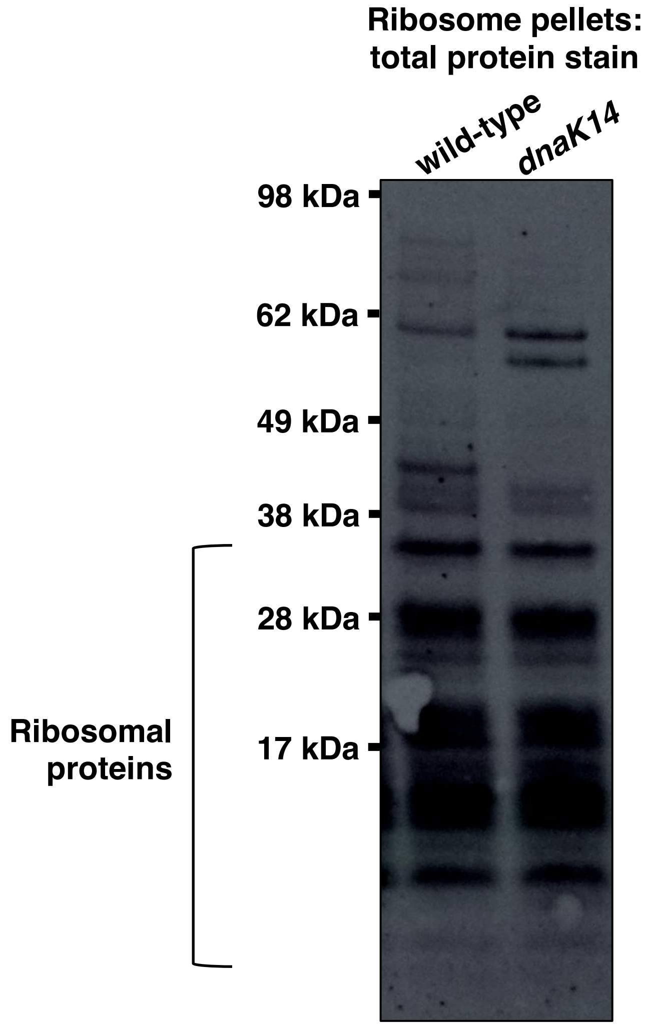

Corresponding to Figure 2H

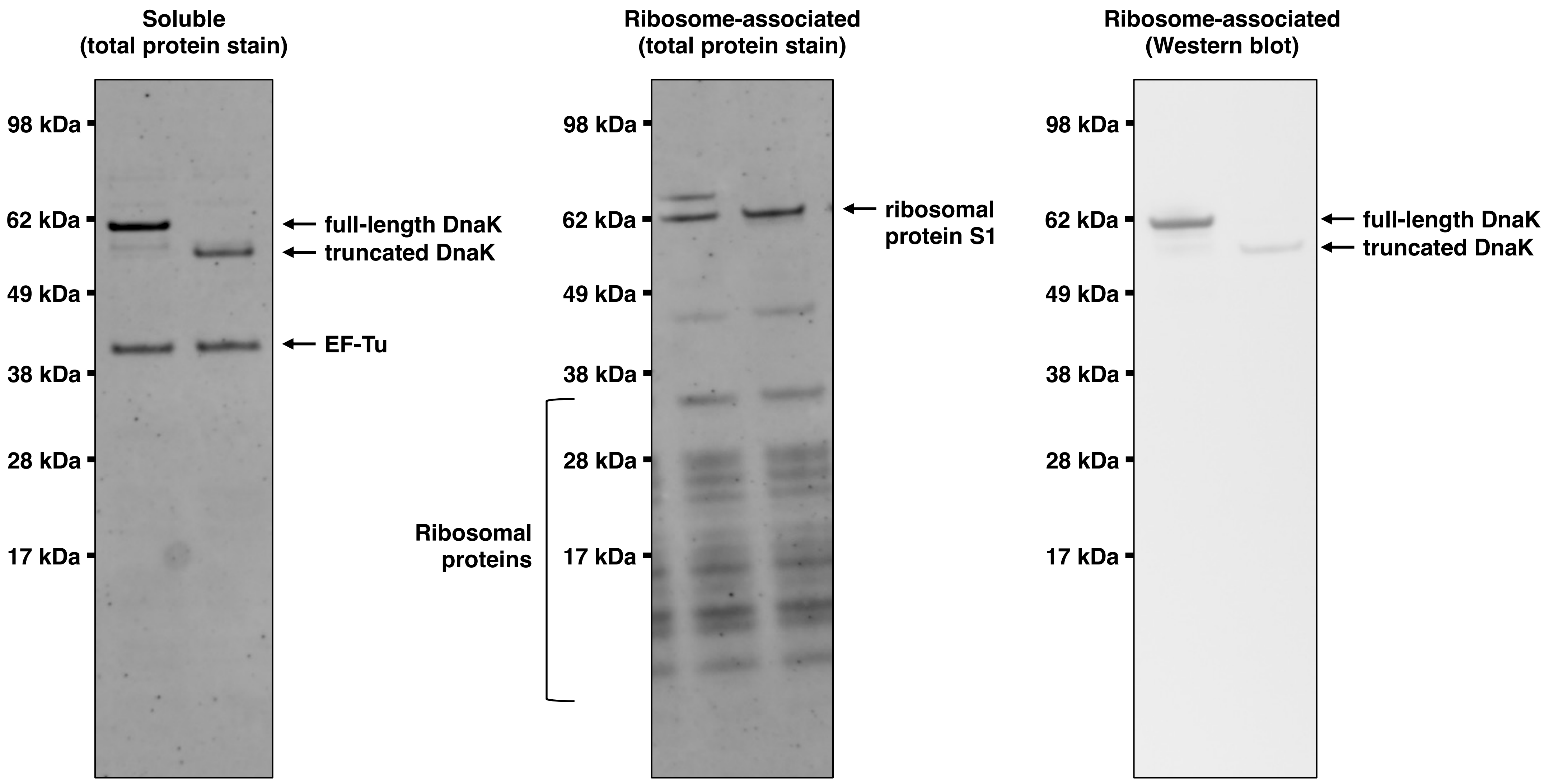

Images and quantifications corresponding to Figures 3A-B

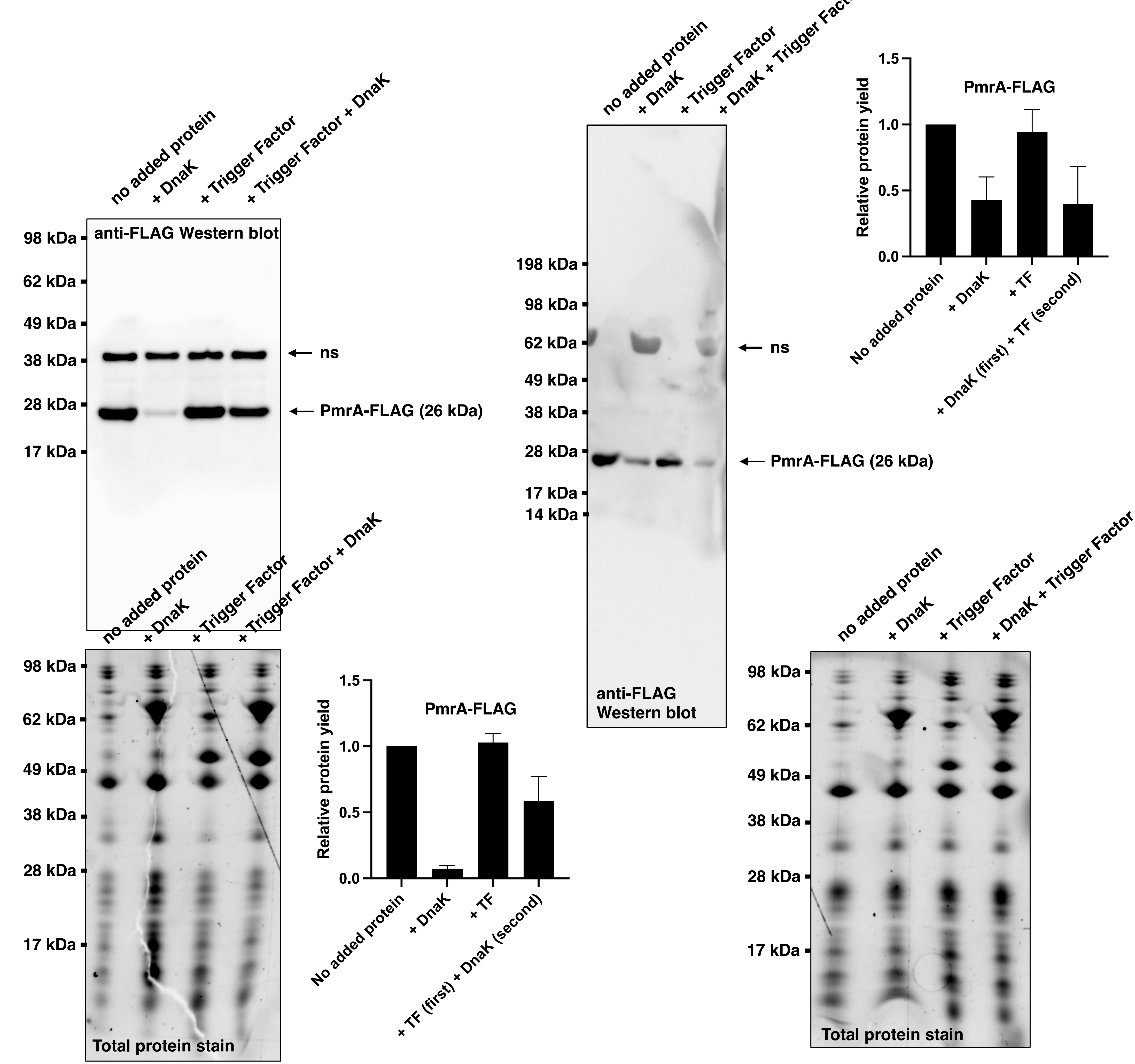

Images and quantifications corresponding to Figure 4A

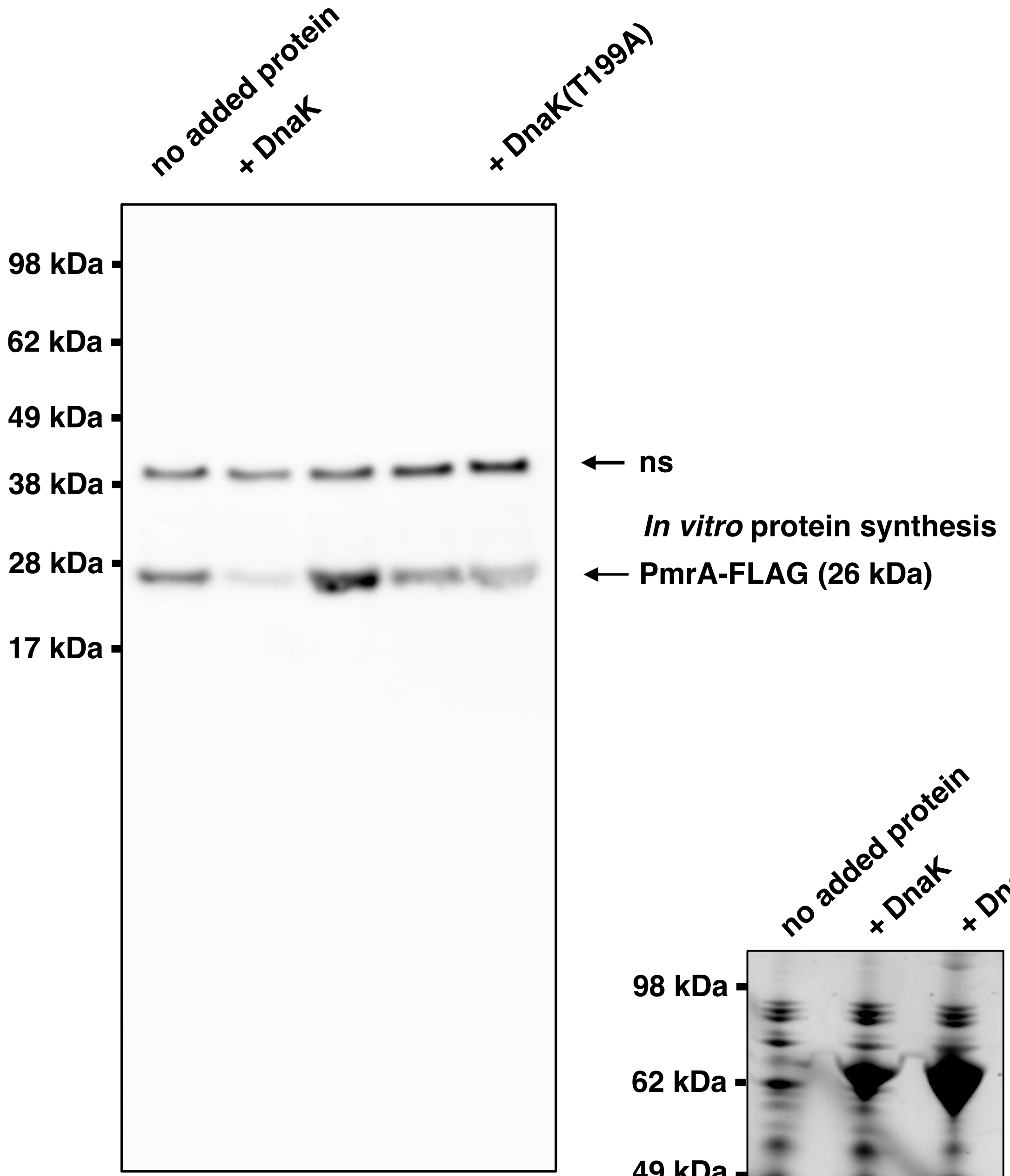

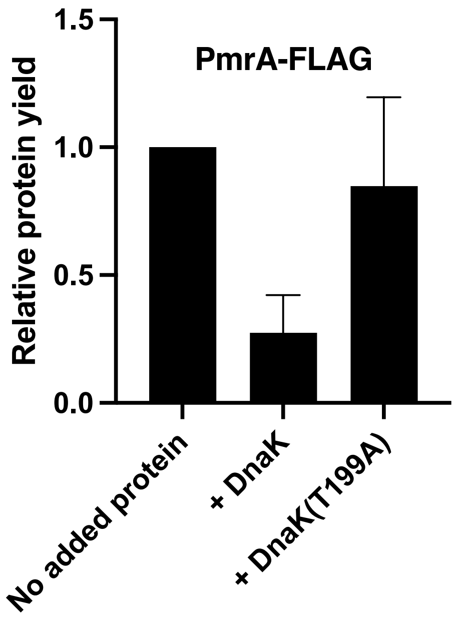

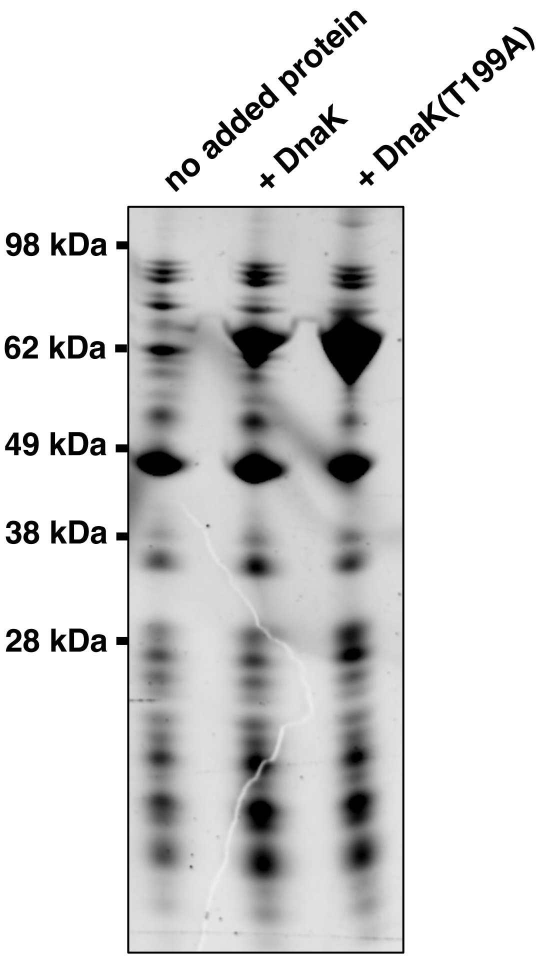

Total protein stain

Images corresponding to Figure 5A

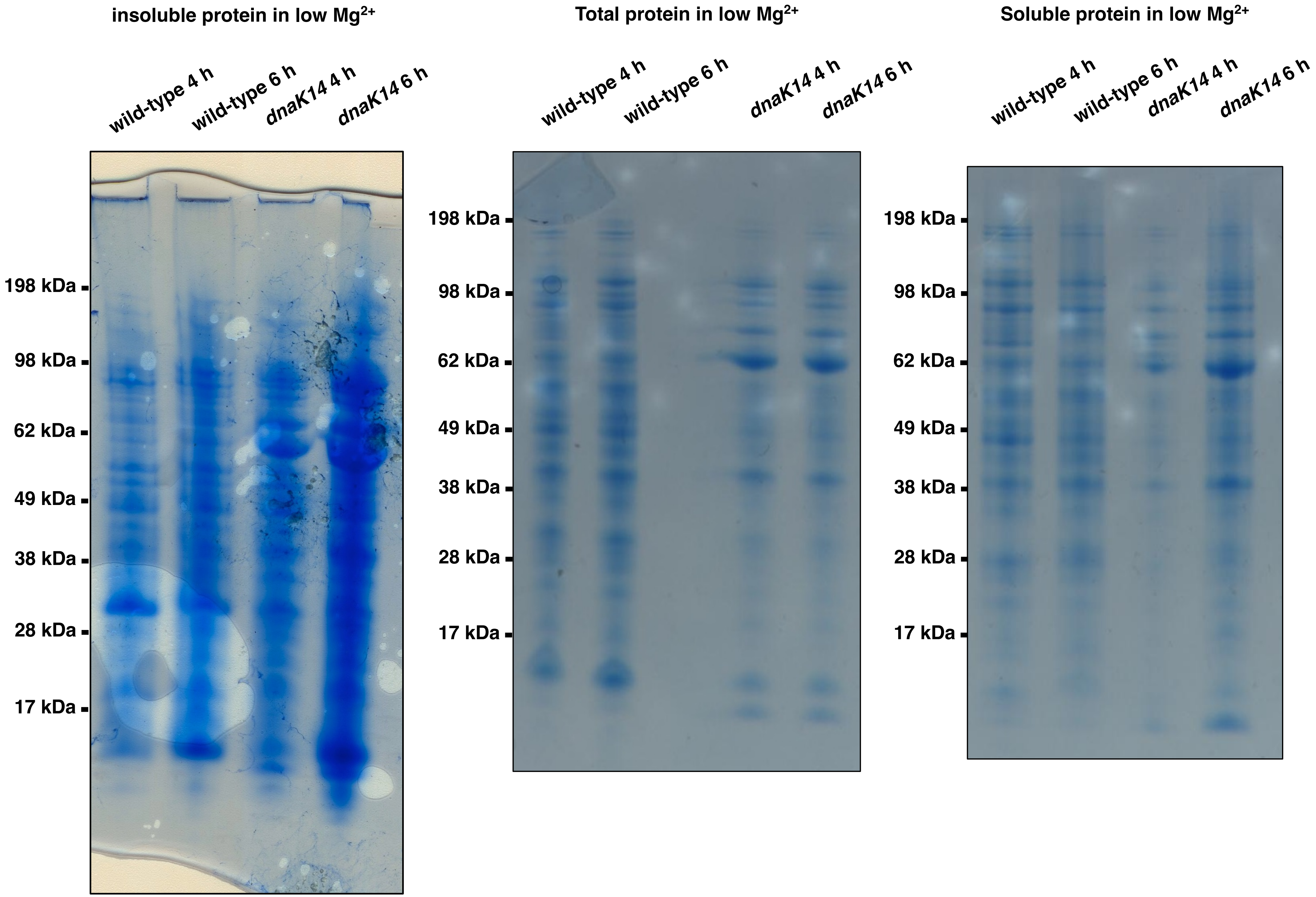

Images corresponding to Figure 5A

insoluble protein in low Mg^{2+}

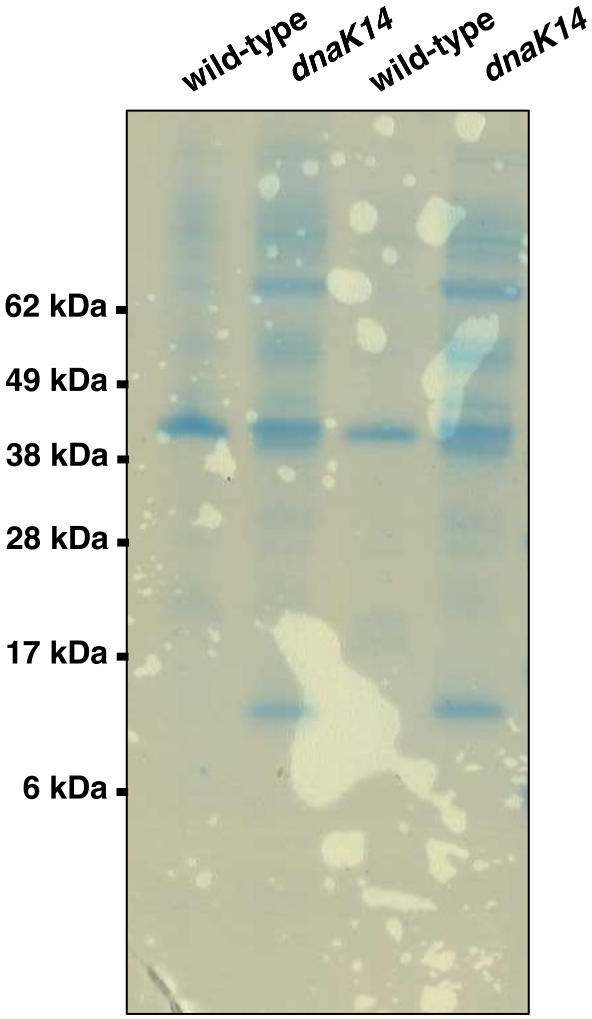

Images corresponding to Figure 5B

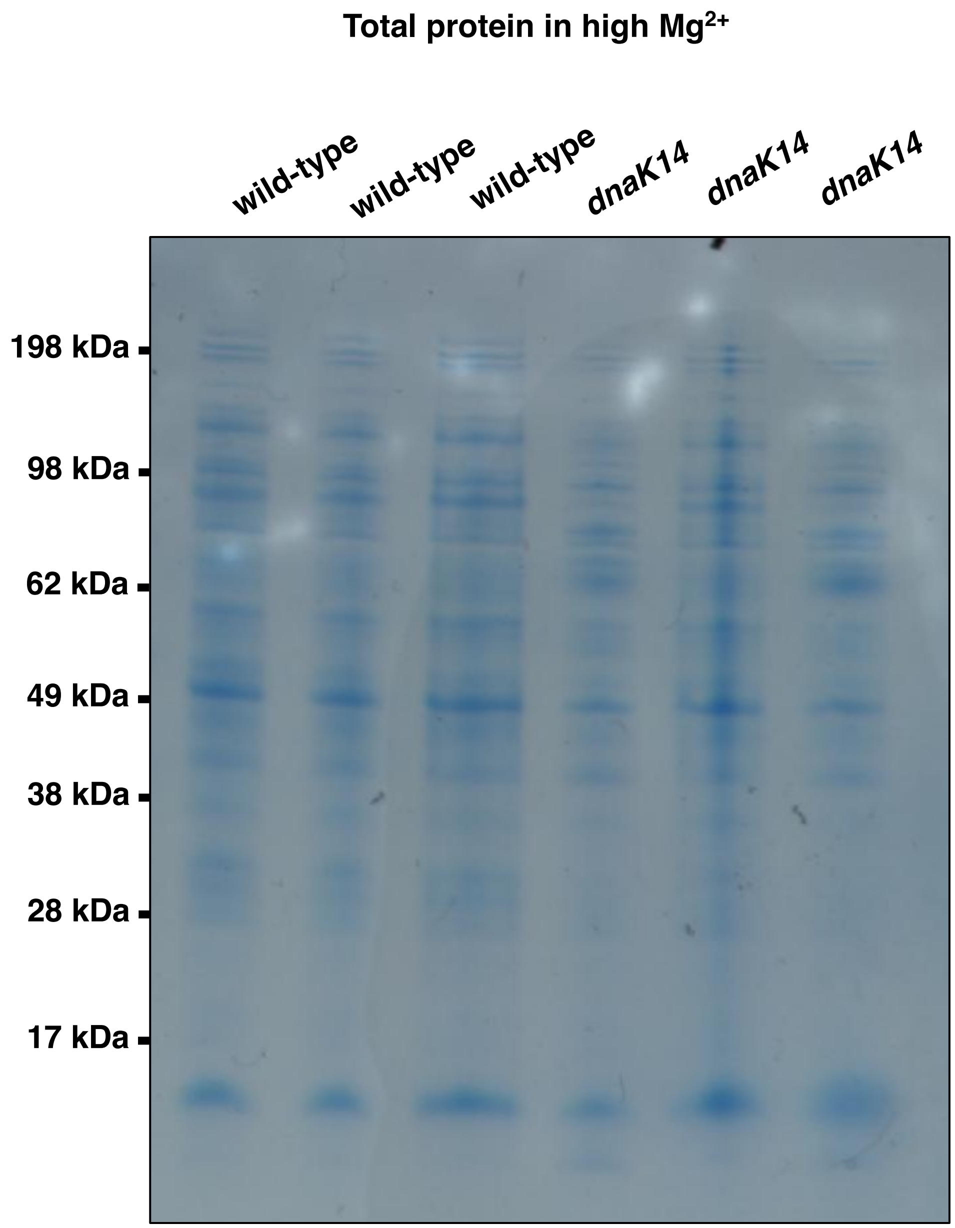

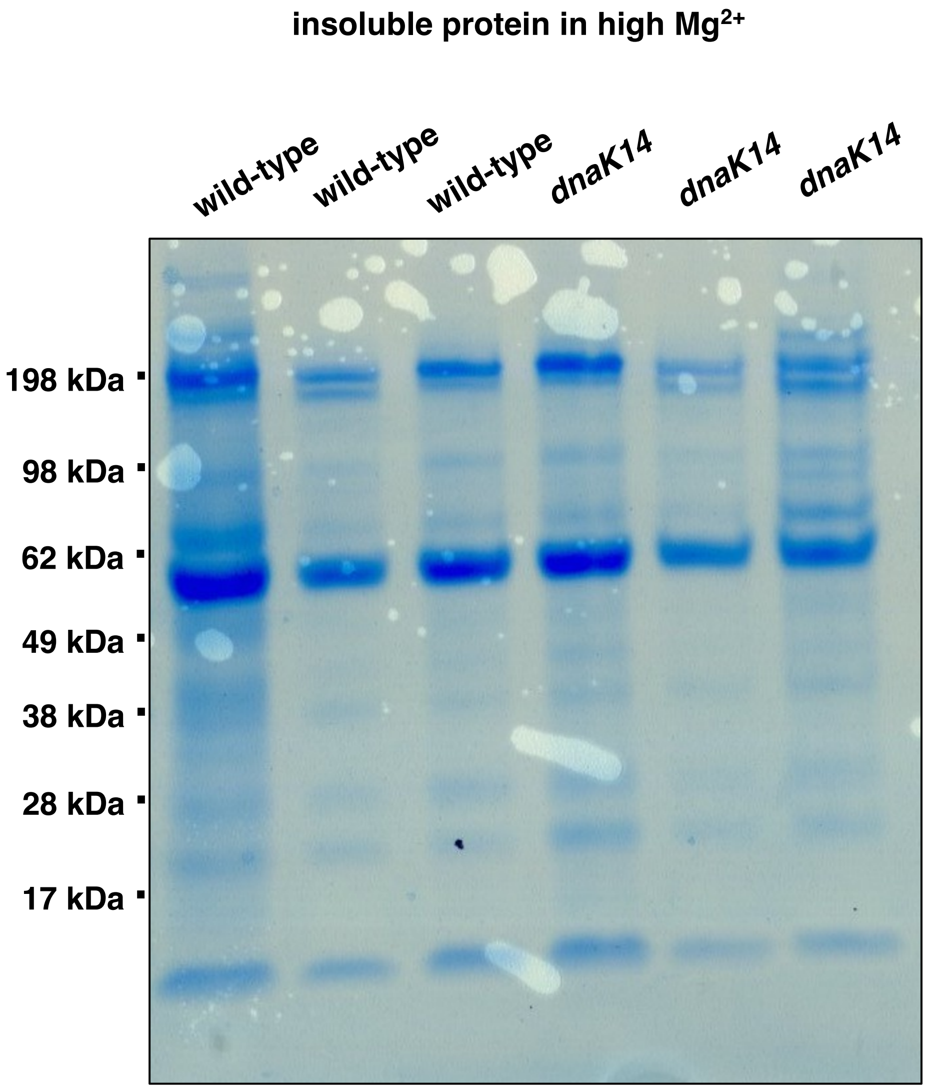

Images corresponding to Figure 5B

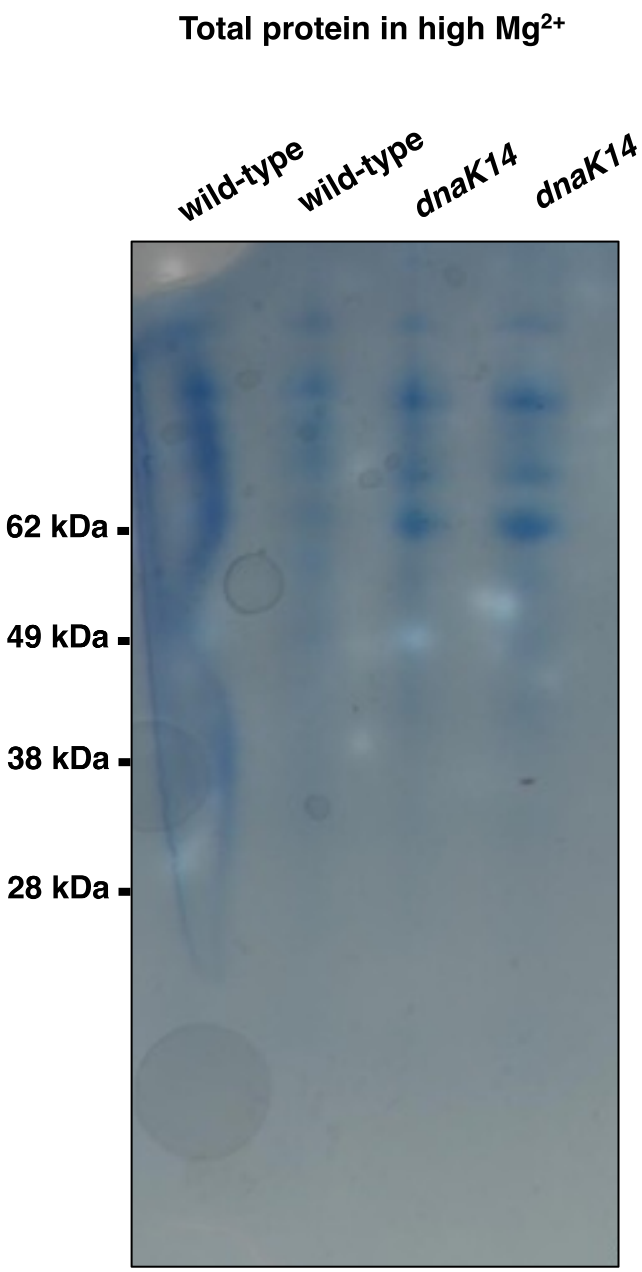

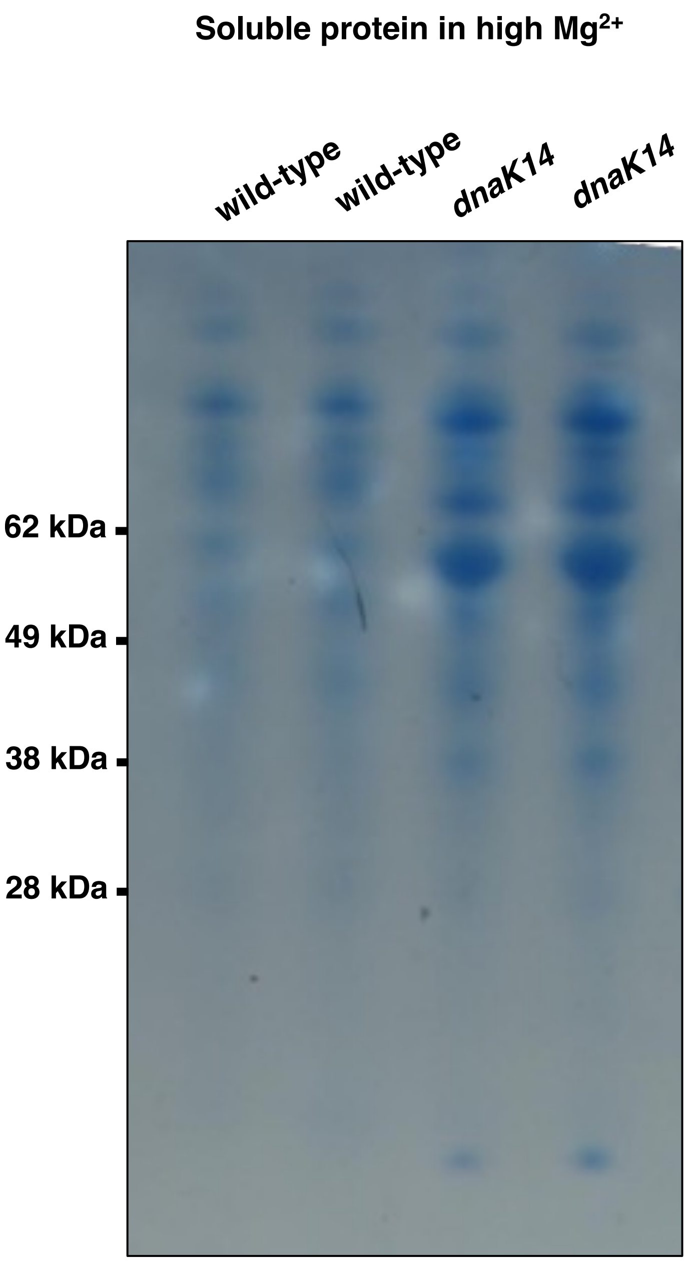

Supplement: S2 Data — (PDF) [file pbio.3002560.s010.pdf]
